# Nickel-Catalyzed Suzuki–Miyaura Coupling in Water for the Synthesis of 2-Aryl Allyl Phosphonates and Sulfones

**DOI:** 10.1021/acs.joc.3c02455

**Published:** 2024-01-26

**Authors:** Yu-Chen Yu, Yun-Chiao Sung, Jun-Hao Fu, Wen-Sheng Peng, Yu-Chia Yu, Juyun Li, Yi-Tsu Chan, Fu-Yu Tsai

**Affiliations:** † Department of Molecular Science and Engineering, National Taipei University of Technology, Taipei 10608, Taiwan; ‡ Institute of Organic and Polymeric Materials, National Taipei University of Technology, Taipei 10608, Taiwan; § Department of Chemistry, National Taiwan University, Taipei 10617, Taiwan

## Abstract

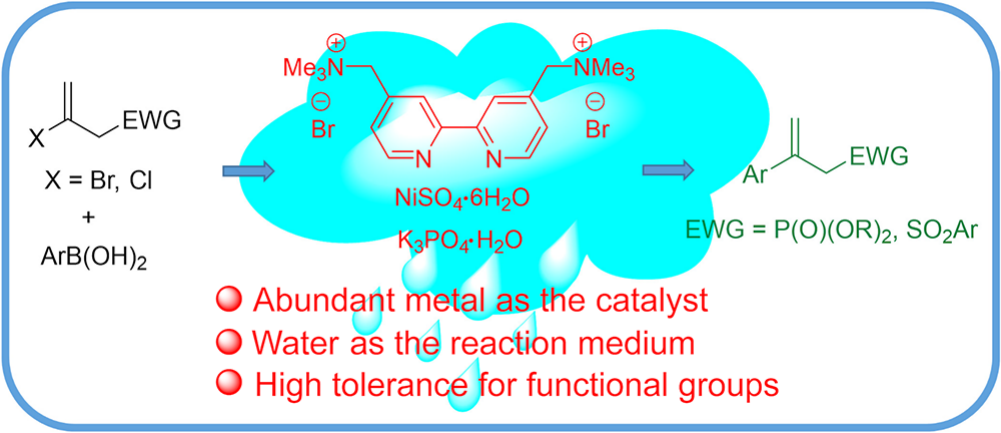

An operationally simple and green protocol using a NiSO_4_·6H_2_O/cationic 2,2′-bipyridyl ligand system as a water-soluble catalyst for the coupling of arylboronic acids with (2-haloallyl)phosphonates and (2-haloallyl)sulfones in water under air was developed. The reaction was performed at 120 °C with arylboronic acids (2 mmol) and (2-haloallyl)phosphonates or sulfones (1 mmol) in the presence of 5 mol % of the Ni catalytic system in a basic aqueous solution for 1 h, giving the corresponding 2-aryl allyl phosphonates or sulfones in good to excellent yields. This reaction features the use of an abundant transition metal as a catalyst in water and exhibits high functional group tolerance, rendering it an eco-friendly procedure.

## Introduction

Allyl phosphonates and sulfones are important structural motifs in pharmaceuticals^1^ and polymeric compounds^2^ and useful starting materials for asymmetric synthesis,^3^*o*-nitration,^4^ desulfonylative electrocarboxylation^5^ and allylation,^6^ cycloaddition,^7^ and allylation onto various substrates.^8^ The classical protocol for the preparation of 2-aryl allyl phosphonates is the reaction of α-bromomethylstyrenes with trialkyl phosphites under reflux conditions. In general, these reactions require multistep synthesis, starting from the coupling of propargylic alcohol and Grignard reagents followed by bromination to obtain various substituted α-bromomethylstyrenes.^3b,3c^ Therefore, the Grignard reagent-sensitive functional group on the aromatic ring should be avoided. Alternatively, photorearrangement of allyl phosphites,^9^ Cu-catalyzed decarboxylative coupling of conjugated β,γ-unsaturated carboxylic acids and diethyl phosphite,^10^ Pd-catalyzed hydroarylation of diphenylphosphorylallenes with arylboronic acids^11^ and olefination of *N*-tosylhydrazones with aryl halide^12^ have also been reported to prepare 2-phenyl allyl phosphonates.

The straightforward method for the preparation of 2-aryl allyl sulfones involves the oxidation of allylic sulfides^13^ and the reaction of α-bromomethylstyrenes with arylsulfinates.^14^ Sulfonyl radical generated from sulfinic acids,^15^ sulfonyl hydrazides catalyzed by Cu under an O_2_ atmosphere,^16^ FeCl_3_,^17^ or tetrabutylammonium iodide (TBAI),^18^ and sodium arylsulfinates catalyzed by I_2_^19^ in the presence of stoichiometric amounts of peroxide have been effectively developed. *p*-Toluenesulfonylmethyl isocyanide (TosMIC) has been used as a sulfonylating agent in the presence of catalytic amounts of I_2_,^20^ Cu,^21^ or Co(salen)_2_^22^ for the formation of 2-aryl allyl sulfones. Additionally, KI-catalyzed electrochemical sulfonylation reactions^23^ and visible light-induced^24^ or Cu(II)-catalyzed three-component reactions^25^ are also applicable. Other efficient methods of light-induced reactions, including surfactant-promoted sulfonylation of α-methylstyrenes with sulfonyl chlorides,^26^ Co(dmgH)_2_pyCl-catalyzed oxidative cross-coupling of α-methylstyrene derivatives with sulfinic acids^27^ or sulfonyl hydrazides,^28^ and Ru(bpy)_3_Cl_2_-catalyzed aryl radical in situ SO_2_-capture reaction^29^ have been reported recently. In addition, I_2_,^30^ ZnI_2_,^4^ and Mg^31^ under sonication are also known to promote sulfonylation of α-methylstyrenes and α-(chloromethyl)styrenes, resulting in the formation of 2-aryl allyl sulfones.

Other transition-metal-catalyzed reactions, including Cu-catalyzed reactions of styrene or α-methylstyrene derivatives with aromatic sulfonyl chlorides,^32^ decarboxylative sulfonylation of conjugated β,γ-unsaturated carboxylic acids,^10^ and oxidative sulfonations of alkenes^33^ have been achieved. On the other hand, cross-coupling reactions such as Ni-catalyzed coupling of β-phenylselanyl allyl sulfone and phenylmagnesium bromide,^34^ allene carbopalladation with aryl halide in the presence of sodium benzenesulfinate or sulfonyl hydrazide,^35^ Pd-catalyzed Suzuki–Miyaura coupling of 2-borylated allylic sulfones with aryl iodides,^36^ and coupling of sulfonyl allylic acetate with B_2_(pin)_2_^37^ have also been developed for the synthesis of 2-aryl allyl sulfones.

**Scheme 1:**
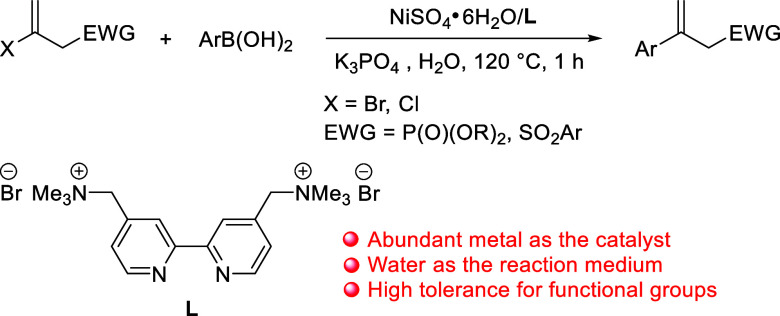
Ni-Catalyzed Coupling of (2-Haloallyl)phosphonates or Sulfones with Arylboronic Acids in Water

Ni-catalyzed Suzuki–Miyaura couplings have attracted much attention recently as they are more economical and earth-abundant than Pd.^38^ To achieve the goal of green chemistry, eco-friendly media are widely employed in Suzuki–Miyaura coupling^39^ and Ni-catalyzed reactions.^40^ One of the best solvents for environmentally friendly chemistry is water, which has been employed in a variety of catalytic reactions to eliminate the use of harmful organic media.^41^ Several reports have described that an excess amount of water is deleterious to Ni-catalyzed Suzuki–Miyaura coupling;^42^ hence, only a few reports have used water as the sole reaction medium, which includes the employment of Ni nanoparticles^43^ and Ni(cod)_2_^44^ as catalysts. The Ni-Sanp complex is also able to catalyze the Suzuki–Miyaura reaction in water; however, the solvent systems of ethanol or ethanol–water (1:1) give higher yields than neat water.^45^ Compared to Ni(0) catalysts, most Ni(II) salts are readily available and exhibit moisture, air, and thermal stability, making them potential precatalysts for conducting cross-coupling reactions in water without the need for prior treatment. In this study, we developed a straightforward and efficient protocol utilizing a NiSO_4_·6H_2_O/cationic 2,2′-bipydyl system to catalyze the cross-coupling of 2-haloallyl phosphonates and sulfones with a broad scope of commercially available arylboronic acids in neat water, giving the corresponding 2-aryl allyl phosphonates and sulfones under environmentally friendly conditions (Scheme 1).

## Results and Discussion

Our studies began with the coupling reaction of diethyl 2-bromoallylphosphonate **1a** (1 mmol) and phenylboronic acid **2a** (1.5 mmol) at 120 °C in the presence of K_2_CO_3_ as the base in water under air. The results are summarized in Table 1. The association of commercially available Ni salts with a cationic 2,2′-bipyridyl ligand, **L**, as the catalytic system was investigated (Entries 1–6). NiSO_4_·6H_2_O was the best catalyst for this coupling, which afforded **3aa** in a 67% yield (Entry 1). Increasing the amount of **2a** to 2 mmol helped to enhance the yield of **3aa** to 93% (Entry 7). Among the choices between bases (Entries 7–15), K_3_PO_4_ was the most efficient for this coupling reaction, and as a result, **3aa** was generated in a nearly quantitative yield (Entry 8). Notably, when a stronger base, such as KOH and NaOH, was used, the formation of α,β-unsaturated isomers was observed (Entries 14 and 15), presumably due to the acidic allylic hydrogen adjacent to the phosphonate group. Further research into the reaction duration revealed that 1 h was adequate to provide **3aa** in a yield of 99% (Entry 16). However, only 68 and 55% of the desired product was produced when the catalyst quantity or reaction temperature was decreased, respectively (Entries 17 and 18). The presence of the water-soluble ligand **L** is crucial for achieving a high yield in this cross-coupling reaction. In the absence of **L** or when it was replaced with a neutral ligand such as 2,2′-bipyridine, no **3aa** was produced and unreacted **1a** was observed (Entries 19 and 20). Even with a phase transfer agent, tetrabutylammonium bromide, the reaction under the conditions of Entry 20 still could not generate **3aa** (Entry 21). To investigate the inductive effect of phosphonates, diethyl (3-bromobut-3-en-1-yl)phosphonate was subjected to the reaction conditions of Entry 16 instead of **1a**. However, no desired product was formed, and diethyl (3-bromobut-3-en-1-yl)phosphonate remained unchanged. This underscores the essential role of (2-bromoallyl)phosphonates in this catalytic reaction.

**Table 1:**
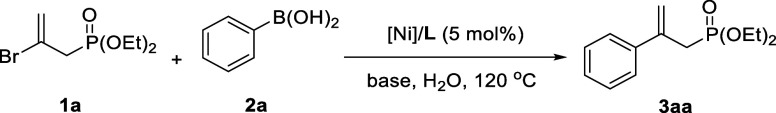
Optimization of the Nickel-Catalyzed Coupling of **1a** and **2a** in Water^a^

entry	[Ni] (mol %)	**2a** (equiv)	base	yield (%)^b^
1	NiSO_4_·6H_2_O	1.5	K_2_CO_3_	67
2	NiCl_2_·6H_2_O	1.5	K_2_CO_3_	37
3	Ni(OH)_2_	1.5	K_2_CO_3_	43
4	NiBr_2_	1.5	K_2_CO_3_	54
5	Ni(OAc)_2_·4H_2_O	1.5	K_2_CO_3_	46
6	Ni(NO_3_)_2_·6H_2_O	1.5	K_2_CO_3_	38
7	NiSO_4_·6H_2_O	2.0	K_2_CO_3_	93
8	NiSO_4_·6H_2_O	2.0	K_3_PO_4_	99
9	NiSO_4_·6H_2_O	2.0	KOAc	69
10	NiSO_4_·6H_2_O	2.0	KF	24
11	NiSO_4_·6H_2_O	2.0	Na_2_CO_3_	47
12	NiSO_4_·6H_2_O	2.0	Cs_2_CO_3_	57
13	NiSO_4_·6H_2_O	2.0	Bu_3_N	29
14	NiSO_4_·6H_2_O	2.0	KOH	46^c^
15	NiSO_4_·6H_2_O	2.0	NaOH	51^d^
16^e^	NiSO_4_·6H_2_O	2.0	K_3_PO_4_	99
17^e^^,^^f^	NiSO_4_·6H_2_O	2.0	K_3_PO_4_	68
18^e^^,^^g^	NiSO_4_·6H_2_O	2.0	K_3_PO_4_	55
19^e^^,^^h^	NiSO_4_·6H_2_O	2.0	K_3_PO_4_	0
20^e^^,^^i^	NiSO_4_·6H_2_O	2.0	K_3_PO_4_	0
21^e^^,^^i^^,^^j^	NiSO_4_·6H_2_O	2.0	K_3_PO_4_	0

a Reaction conditions: **1a** (1.0 mmol), **2a**, [Ni]/**L** (5 mol %), base (1.5 mmol), and H_2_O (4 mL) at 120 °C for 3 h.

b Isolated yield.

c Combined yield of **3aa** and the isomer with a molar ratio of 16:1 determined by ^1^H NMR.

d Combined yield of **3aa** and the isomer with a molar ratio of 26:1 determined by ^1^H NMR.

e Reaction time was 1 h.

f At 100 °C.

g NiSO_4_·6H_2_O/**L** (2.5 mol %).

h In the absence of **L**.

i Neutral 2,2′-bipyridine was used instead of **L**.

j 20 mol % of tetrabutylammonium bromide was added.

With the optimized cross-coupling conditions (Table 1, Entry 16), the scope of arylboronic acids **2** in this reaction (Table 2) was examined. When arylboronic acids possessing activating and weakly deactivating groups at the *para*-position were applied, this Ni-catalyzed Suzuki–Miyaura coupling proceeded smoothly, giving the corresponding products, **3ab**–**3af**, in yields between 65 and 92%. Notably, the reaction is applicable to Grignard reagent-sensitive functional groups such as ketone, cyanide, nitro, ester, and amino substituents on arylboronic acids, resulting in corresponding products **3ag**–**3ak** in high yields. Thus, this reaction provides a convenient and simple procedure to prepare a great substrate scope of 2-aryl allyl phosphonates containing organometallic reagent-sensitive functional groups.^3b,3c^ The *meta*-substituted, disubstituted aryl, and 2-naphthyl boronic acids were also suitable for this reaction, which gave **3al**–**3ao** in yields between 60 and 95%. The sterically congested *ortho*-substituted arylboronic acids made the reaction slower, so **3ap** and **3aq** were both obtained in only 59% yields. The introduction of more sterically hindered 2,6-dimethoxyphenylboronic acid resulted in a complete blockage of the coupling reaction (**3as**).

**Table 2:**
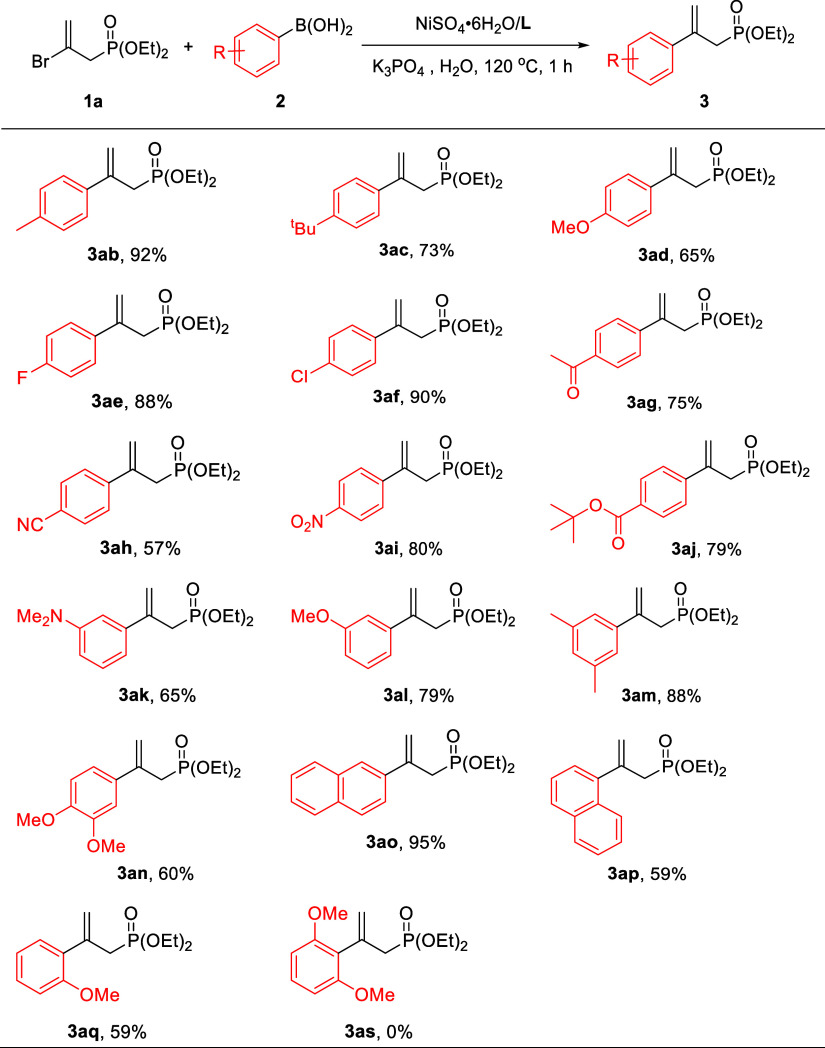
Synthesis of Diethyl (2-Arylallyl)phosphonates (**3**)^a^

a Reaction conditions: **1a** (1.0 mmol), **2** (2.0 mmol), NiSO_4_·6H_2_O/**L** (5 mol %), K_3_PO_4_ (1.5 mmol), and H_2_O (4 mL) at 120 °C for 1 h; isolated yields for **3**.

To further explore the scope of this protocol, the cross-coupling of 2-bromoallyl sulfone **4a** and arylboronic acids **2** (Table 3) was investigated. The Suzuki–Miyaura coupling of **4a** and **2a** took place very efficiently, furnishing **5aa** in a near quantitative yield under the conditions of Table 1, Entry 16. In comparison with the published Pd- and Ni-catalyzed cross-coupling reactions for the synthesis of 2-aryl allyl sulfones,^34−37^ phosphine or arsine ligands were not required in our system. Furthermore, electron-rich, electron-deficient, *meta*-substituted, disubstituted aryl, and 2-naphthyl boronic acids were coupled with **4a** smoothly, leading to the desired products in high yields (**5ab**–**5ao**). Similar to the coupling of **1a**, the reaction is influenced by the substituted position on arylboronic acids. Consequently, when sterically hindered arylboronic acids were employed as the coupling partner, the desired 2-aryl allyl sulfones were obtained in approximately 50% yield (as seen with **5ap** and **5ar**), but **5as** was not found when 2,6-dimethoxyphenylboronic acid was employed.

**Table 3:**
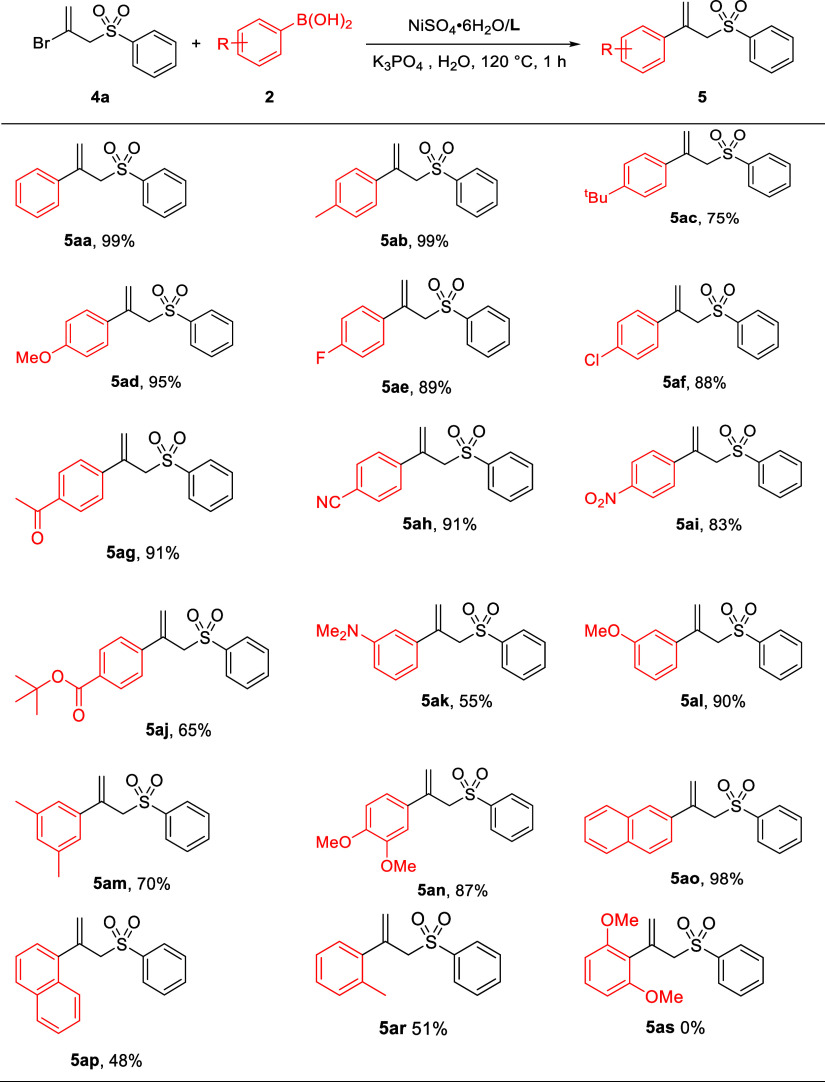
Synthesis of ((2-Arylallyl)sulfonyl)benzenes (**5**)^a^

a Reaction conditions: **4a** (1.0 mmol), **2** (2.0 mmol), NiSO_4_·6H_2_O/**L** (5 mol %), K_3_PO_4_ (1.5 mmol), and H_2_O (4 mL) at 120 °C for 1 h; isolated yields for **5**.

Finally, (2-chloroallyl)phosphonate **6a** and sulfone **7a** were also found to be viable substrates for coupling with arylboronic acids. Table 4 illustrates that the chloride reactants participated in the reaction with slightly lower efficacy than their bromide counterparts (Tables 2 and 3). This suggests that oxidative addition might be the rate-determining step.

**Table 4:**
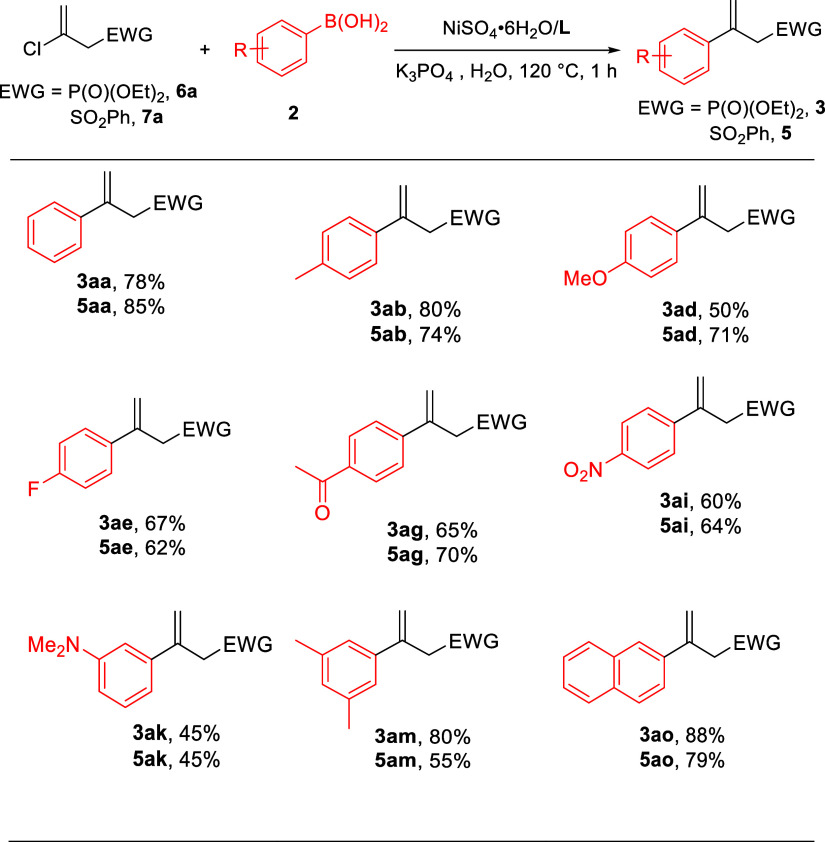
Nickel-Catalyzed Coupling of **6a** and **7a** with **2** in Water^a^

a Reaction conditions: **6a** or **7a** (1.0 mmol), **2** (2.0 mmol), NiSO_4_·6H_2_O/**L** (5 mol %), K_3_PO_4_ (1.5 mmol), and H_2_O (4 mL) at 120 °C for 1 h; isolated yields for **3** or **5**.

**Scheme 2:**
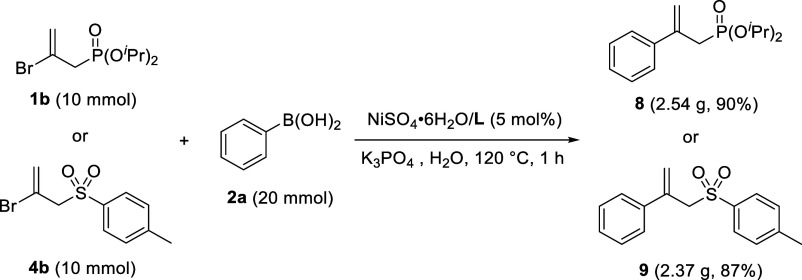
Gram-Scale Reactions

To assess the potential for future practical applications, the reaction was successfully scaled up from a small scale (1 mmol) to a gram scale (10 mmol). As representatives, diisopropyl (2-bromoallyl)phosphonate **1b** and β-bromoallyl *p*-tolyl sulfone **4b** (10 mmol) were chosen to be coupled with **2a** (20 mmol). These large-scale reactions were done in the presence of NiSO_4_·6H_2_O/**L** (5 mol %) and K_3_PO_4_ (15 mmol) in H_2_O (40 mL) at 120 °C for 1 h, yielding **8** and **9** in 90 and 87% yields, respectively (Scheme 2).

**Scheme 3:**
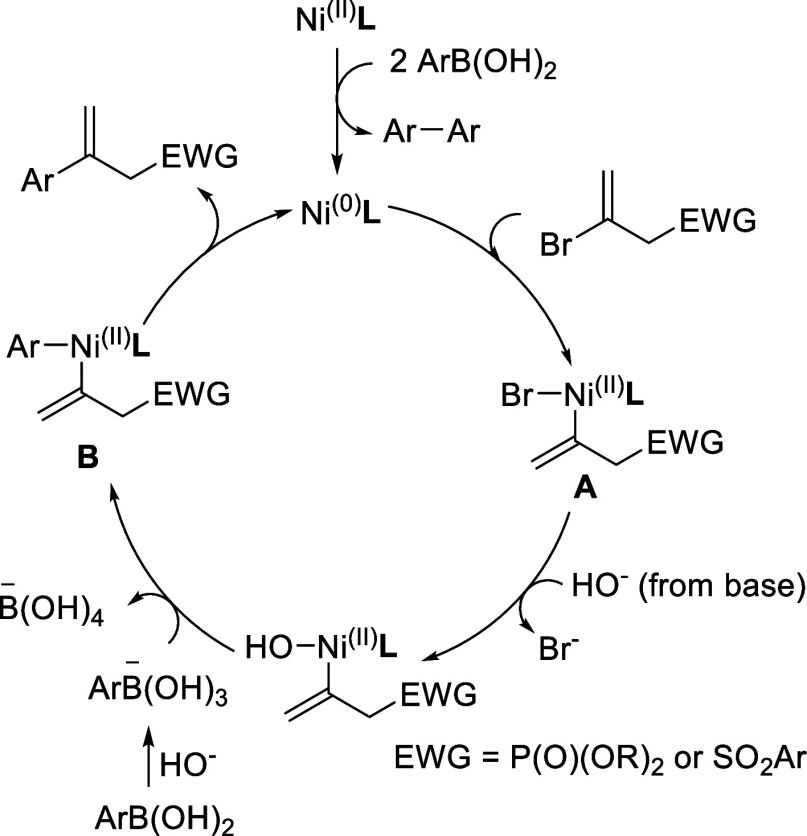
Proposed Mechanism

The coupling reactions of **1a** or **4a** with **2a** were not suppressed in the presence of 1 equiv of 2,2,6,6-tetramethylpiperidin-1-yl)oxyl (TEMPO) under the conditions shown in Table 1, Entry 16, implying that the Ni(I)/Ni(III) process may be unlikely. Although the radical process cannot be completely excluded, a mechanism via Ni(0)/Ni(II) oxidation states similar to the typical Pd-catalyzed mechanism was proposed (Scheme 3).^46^ The process begins with the double transmetalation of arylboronic acid to Ni(II), followed by reductive elimination, resulting in the formation of Ni(0). The oxidative addition of compounds **1** or **4** to Ni(0) leads to the creation of intermediate **A**. The replacement of bromide by hydroxide is followed by the transmetalation of arylboronic acid, facilitating the formation of **B**. Subsequently, a reductive elimination step affords the product and regenerates Ni(0) for the next cycle. It is worth noting that it is possible to activate C–P and C–S bonds to yield 2,3-diarylpropenes through diarylation. However, no such byproducts were found in our system.

## Conclusions

In summary, this study introduces a straightforward and environmentally friendly method for the synthesis of 2-aryl allyl phosphonates and sulfones, featuring a wide range of functional group variations. The procedure utilizes readily accessible NiSO_4_·6H_2_O as an economical precatalyst, accommodates a diverse set of arylboronic acids as coupling partners, and relies solely on water as the reaction solvent. Furthermore, the high yields achieved in large-scale reactions indicate the potential for practical applications.

## Experimental Section

### General Information

Chemicals including Ni salts and arylboronic acids **2** were obtained from the chemical suppliers and used as received without further purification. **4a**,^47^**4b**,^8b^**6a**,^48^ and **7a**(8g) were synthesized according to known procedures. The water-soluble cationic 2,2′-bipyridyl ligand, **L**, was synthesized using our published procedure.^49^ The NiSO_4_**·**6H_2_O/**L** aqueous solution (0.05 mmol in 1.5 mL H_2_O) was prepared by stirring equal molar quantities of NiSO_4_**·**6H_2_O and **L** in water for 10 min and used immediately. ^1^H- and ^13^C{^1^H}-NMR were conducted at 25 °C in CDCl_3_ on a Bruker Biospin AG 300 and JEOL JNM-ECZS 400 NMR spectrometers. The chemical shifts (δ in ppm) were established with respect to CHCl_3_, which was employed as a reference (^1^H NMR: CHCl_3_ at 7.24 ppm; ^13^C{^1^H} NMR: CDCl_3_ at 77.0 ppm). ^31^P{^1^H} NMR spectra were recorded in CDCl_3_ at 25 °C on a Varian 400 NMR spectrometer using 85% H_3_PO_4_ as an external standard. The melting points of solid products were recorded on an SRS-MPA120 melting point apparatus. High-resolution mass spectra (HRMS) for unknown products were acquired on a Waters Synapt HDMS G2 instrument with a LockSpray ESI source. Copies of the NMR spectra for compounds **1** and **3**–**9** can be found in the ESI.

#### Preparation of Dialkyl (2-Bromoallyl)phosphonate (**1**)

2,3-Dibromopropene (3.1 mL, 30 mmol, 1.0 equiv) and trialkyl phosphite (4.0 mL for triethyl phosphite and 9.1 mL for triisopropyl phosphite, 33 mmol, 1.1 equiv) were stirred in a Schlenk tube at 120 °C under an N_2_ atmosphere for 12 h. After the reaction, low boiling point starting materials were removed under vacuum, and the desired products were obtained via reduced pressure distillation (90 °C/0.2 Torr for **1a**; 100 °C/0.2 Torr for **1b**).

#### Diethyl (2-Bromoallyl)phosphonate (**1a**)

Colorless oil (6.94 g, 90%); ^1^H NMR (300 MHz, CDCl_3_): δ 5.83–5.80 (m, 1H), 5.61 (dd, *J* = 4.8, 2.0 Hz, 1H), 4.15–4.10 (m, 4H), 3.03 (dd, *J* = 21.4, 1.0 Hz, 2H), 1.31 (t, *J* = 7.1 Hz, 6H); ^13^C{^1^H} NMR (75 MHz, CDCl_3_): δ 121.7 (d, *J*_C–P_ = 10.2 Hz), 121.0 (d, *J*_C–P_ = 11.5 Hz), 62.4 (d, *J*_C–P_ = 6.6 Hz), 39.2 (d, *J*_C–P_ = 139.7 Hz), 16.3 (d, *J*_C–P_ = 6.2 Hz); ^31^P{^1^H} NMR (162 MHz, CDCl_3_): δ 22.76; HRMS (MALDI-TOF) calcd for C_7_H_15_BrO_3_P [M + H]^+^: 256.9942, found 256.9937.

#### Diisopropyl (2-Bromoallyl)phosphonate (**1b**)

Colorless oil (4.86 g, 57%); ^1^H NMR (300 MHz, CDCl_3_): δ 5.85–5.82 (m, 1H), 5.61 (dd, *J* = 4.7, 2.0 Hz, 1H), 4.77–4.66 (m, 2H), 3.00 (dd, *J* = 21.3, 1.1 Hz, 2H), 1.31 (d, *J* = 6.2 Hz, 12H); ^13^C{^1^H} NMR (75 MHz, CDCl_3_): δ 121.4 (d, *J*_C–P_ = 10.2 Hz), 71.0 (d, *J*_C–P_ = 6.8 Hz), 41.1 (d, *J*_C–P_ = 140.8 Hz), 24.0 (d, *J*_C–P_ = 3.9 Hz), 23.8 (d, *J*_C–P_ = 5.1 Hz); ^31^P{^1^H} NMR (162 MHz, CDCl_3_): δ 20.68; HRMS (MALDI-TOF) calcd for C_9_H_19_BrO_3_P [M + H]^+^: 285.0255, found 285.0252.

#### General Procedure for the Synthesis of Diethyl (2-Arylallyl)phosphonates (**3**)

A 20 mL sealable glass reactor equipped with a magnetic stirrer bar was charged with arylboronic acid (2 mmol, 2.0 equiv), K_3_PO_4_ (318 mg, 1.5 mmol, 1.5 equiv), **1a** or **6a** (257 mg for **1a** or 213 mg for **6a**, 1.0 mmol, 1.0 equiv), and 2.5 mL of H_2_O. After adding NiSO_4_**·**6H_2_O/**L** (13/23 mg, 0.05 mmol, 0.05 equiv, in 1.5 mL of H_2_O), the reaction tube was sealed, and the reaction was stirred at 120 °C (oil bath) for 1 h. After cooling the reaction to room temperature, the aqueous solution was extracted with EtOAc (3 × 4 mL). The combined organic solution was dried over MgSO_4_ and the solvent was removed under reduced pressure. Column chromatography on silica gel provided the desired products.

#### Diethyl (2-Phenylallyl)phosphonate (**3aa**)^3b^

Colorless oil (252 mg, 99% from **1a**; 198 mg, 78% from **6a**); purification by silica gel chromatography (eluent: EtOAc, *R*_f_ = 0.36); ^1^H NMR (300 MHz, CDCl_3_): δ 7.47–7.43 (m, 2H), 7.34–7.25 (m, 3H), 5.50 (dd, *J* = 5.6, 1.0 Hz, 1H), 5.33 (dd, *J* = 5.6, 1.0 Hz, 1H), 4.02–3.93 (m, 4H), 3.05 (dd, *J* = 22.2, 1.0 Hz, 2H), 1.17 (t, *J* = 7.1 Hz, 6H); ^13^C{^1^H} NMR (75 MHz, CDCl_3_): δ 140.6 (d, *J*_C–P_ = 4.3 Hz), 138.6 (d, *J*_C–P_ = 10.3 Hz), 128.2, 127.7, 126.2, 117.2 (d, *J*_C–P_ = 11.0 Hz), 62.0 (d, *J*_C–P_ = 6.8 Hz), 32.9 (d, *J*_C–P_ = 137.9 Hz), 16.2 (d, *J*_C–P_ = 6.3 Hz); ^31^P{^1^H} NMR (162 MHz, CDCl_3_): δ 26.37.

#### Diethyl (2-(*p*-Tolyl)allyl)phosphonate (**3ab**)^3b^

Colorless oil (247 mg, 92% from **1a**; 215 mg, 80% from **6a**); purification by silica gel chromatography (eluent: EtOAc, *R*_f_ = 0.38); ^1^H NMR (300 MHz, CDCl_3_): δ 7.34 (d, *J* = 8.3 Hz, 2H) 7.11 (d, *J* = 8.3 Hz, 2H), 5.47 (d, *J* = 5.6 Hz, 1H), 5.28 (d, *J* = 5.6 Hz, 1H), 4.04–3.90 (m, 4H), 3.02 (d, *J* = 22.2 Hz, 2H), 2.31 (s, 3H), 1.18 (t, *J* = 7.1 Hz, 6H); ^13^C{^1^H} NMR (75 MHz, CDCl_3_): δ 138.3 (d, *J*_C–P_ = 10.1 Hz), 137.6 (d, *J*_C–P_ = 4.2 Hz), 137.5, 128.9, 126.1, 116.4 (d, *J*_C–P_ = 10.8 Hz), 62.0 (d, *J*_C–P_ = 6.6 Hz), 32.9 (d, *J*_C–P_ = 137.7 Hz), 21.1, 16.3 (d, *J*_C–P_ = 6.2 Hz); ^31^P{^1^H} NMR (162 MHz, CDCl_3_): δ 26.54.

#### Diethyl (2-(4-(*tert*-Butyl)phenyl)allyl)phosphonate (**3ac**)

Colorless oil (227 mg, 73%); purification by silica gel chromatography (eluent: EtOAc, *R*_f_ = 0.44); ^1^H NMR (300 MHz, CDCl_3_): δ 7.40–7.30 (m, 4H), 5.47 (dd, *J* = 5.5, 1.0 Hz, 1H), 5.28 (dd, *J* = 5.5, 1.0 Hz, 1H), 4.03–3.89 (m, 4H), 3.03 (dd, *J* = 22.1, 1.0 Hz, 2H), 1.28 (s, 9H), 1.16 (t, *J* = 7.1 Hz, 6H); ^13^C{^1^H} NMR (75 MHz, CDCl_3_): δ 150.7, 138.3 (d, *J*_C–P_ = 10.2 Hz), 137.6 (d, *J*_C–P_ = 4.2 Hz), 125.9, 125.1, 116.4 (d, *J*_C–P_ = 10.9 Hz), 61.9 (d, *J*_C–P_ = 6.5 Hz), 34.4, 32.9 (d, *J*_C–P_ = 137.8 Hz), 31.2, 16.2 (d, *J*_C–P_ = 6.3 Hz); ^31^P{^1^H} NMR (162 MHz, CDCl_3_): δ 26.51; HRMS (MALDI-TOF) calcd for C_17_H_28_O_3_P [M + H]^+^: 311.1776, found 311.1779.

#### Diethyl (2-(4-Methoxyphenyl)allyl)phosphonate (**3ad**)^3b^

Colorless oil (185 mg, 65% from **1a**; 143 mg, 50% from **6a**); purification by silica gel chromatography (eluent: EtOAc, *R*_f_ = 0.33); ^1^H NMR (300 MHz, CDCl_3_): δ 7.39 (d, *J* = 8.9 Hz, 2H), 6.83 (d, *J* = 8.9 Hz, 2H), 5.42 (dd, *J* = 5.6, 0.9 Hz, 1H), 5.22 (dd, *J* = 5.6, 0.9 Hz, 1H), 4.01–3.94 (m, 4H), 3.78 (s, 3H), 3.01 (dd, *J* = 22.2, 0.9 Hz, 2H), 1.19 (t, *J* = 7.1 Hz, 6H); ^13^C{^1^H} NMR (75 MHz, CDCl_3_): δ 159.2, 137.8 (d, *J*_C–P_ = 10.3 Hz), 132.9 (d, *J*_C–P_ = 4.1 Hz), 127.4, 115.5 (d, *J*_C–P_ = 11.0 Hz), 113.5, 62.0 (d, *J*_C–P_ = 6.6 Hz), 55.2, 33.0 (d, *J*_C–P_ = 137.7 Hz), 16.2 (d, *J*_C–P_ = 6.1 Hz); ^31^P{^1^H} NMR (162 MHz, CDCl_3_): δ 26.77.

#### Diethyl (2-(4-Fluorophenyl)allyl)phosphonate (**3ae**)^3b^

Colorless oil (240 mg, 88% from **1a**; 183 mg, 67% from **6a**); purification by silica gel chromatography (eluent: EtOAc, *R*_f_ = 0.38); ^1^H NMR (300 MHz, CDCl_3_): δ 7.43–7.39 (m, 2H), 7.00–6.95 (m, 2H), 5.42 (d, *J* = 5.6 Hz, 1H), 5.28 (d, *J* = 5.6 Hz, 1H), 4.03–3.91 (m, 4H), 2.97 (d, *J* = 22.2 Hz, 2H), 1.17 (t, *J* = 7.1 Hz, 6H); ^13^C{^1^H} NMR (75 MHz, CDCl_3_): δ 162.4 (d, *J*_C–F_ = 245.3 Hz), 137.6 (d, *J*_C–P_ = 10.5 Hz), 136.6 (d, *J*_C–P_ = 3.5 Hz), 127.9 (d, *J*_C–F_ = 8.0 Hz), 117.1 (d, *J*_C–P_ = 10.8 Hz), 115.0 (d, *J*_C–F_ = 21.3 Hz), 62.0 (d, *J*_C–P_ = 6.7 Hz), 33.1 (d, *J*_C–P_ = 138.1 Hz), 16.2 (d, *J*_C–P_ = 6.2 Hz); ^31^P{^1^H} NMR (162 MHz, CDCl_3_): δ 26.13.

#### Diethyl (2-(4-Chlorophenyl)allyl)phosphonate (**3af**)^3b^

Colorless oil (260 mg, 90%); purification by silica gel chromatography (eluent: EtOAc, *R*_f_ = 0.33); ^1^H NMR (300 MHz, CDCl_3_): δ 7.41–7.37 (m, 2H), 7.30–7.26 (m, 2H), 5.48 (d, *J* = 5.6 Hz, 1H), 5.33 (d, *J* = 5.6 Hz, 1H), 4.05–3.94 (m, 4H), 3.00 (d, *J* = 22.1 Hz, 2H), 1.20 (t, *J* = 7.1 Hz, 6H); ^13^C{^1^H} NMR (75 MHz, CDCl_3_): δ 138.7 (d, *J*_C–P_ = 4.0 Hz), 137.3 (d, *J*_C–P_ = 10.5 Hz), 133.2, 128.0, 127.3, 117.4 (d, *J*_C–P_ = 11.0 Hz), 61.8 (d, *J*_C–P_ = 6.7 Hz), 32.6 (d, *J*_C–P_ = 138.3 Hz), 15.9 (d, *J*_C–P_ = 6.1 Hz); ^31^P{^1^H} NMR (162 MHz, CDCl_3_): δ 26.03.

#### Diethyl (2-(4-Acetylphenyl)allyl)phosphonate (**3ag**)

Colorless oil (222 mg, 75% from **1a**; 192 mg, 65% from **6a**); purification by silica gel chromatography (eluent: EtOAc, *R*_f_ = 0.27); ^1^H NMR (300 MHz, CDCl_3_): δ 7.89–7.84 (m, 2H), 7.53–7.49 (m, 2H), 5.56 (d, *J* = 5.5 Hz, 1H), 5.39 (d, *J* = 5.5 Hz, 1H), 4.02–3.91 (m, 4H), 3.01 (d, *J* = 22.1 Hz, 2H), 2.53 (s, 3H), 1.18–1.12 (m, 6H); ^13^C{^1^H} NMR (75 MHz, CDCl_3_): δ 197.5, 145.0 (d, *J*_C–P_ = 3.8 Hz), 137.8 (d, *J*_C–P_ = 10.4 Hz), 136.1, 128.3, 126.3, 119.1 (d, *J*_C–P_ = 10.8 Hz), 62.0 (d, *J*_C–P_ = 6.6 Hz), 32.7 (d, *J*_C–P_ = 138.4 Hz), 26.5, 16.1 (d, *J*_C–P_ = 6.1 Hz); ^31^P{^1^H} NMR (162 MHz, CDCl_3_): δ 26.00; HRMS (MALDI-TOF) calcd for C_15_H_22_O_4_P [M + H]^+^: 297.1256, found 297.1253.

#### Diethyl (2-(4-Cyanophenyl)allyl)phosphonate (**3ah**)

Colorless oil (159 mg, 57%); purification by silica gel chromatography (eluent: EtOAc, *R*_f_ = 0.30); ^1^H NMR (300 MHz, CDCl_3_): δ 7.59–7.52 (m, 4H), 5.55 (d, *J* = 5.5 Hz, 1H), 5.42 (d, *J* = 5.5 Hz, 1H), 4.01–3.94 (m, 4H), 2.99 (dd, *J* = 22.2, 0.9 Hz, 2H), 1.20–1.14 (m, 6H); ^13^C{^1^H} NMR (75 MHz, CDCl_3_): δ 145.0 (d, *J*_C–P_ = 3.9 Hz), 137.4 (d, *J*_C–P_ = 10.6 Hz), 132.0, 126.9, 119.9 (d, *J*_C–P_ = 11.0 Hz), 118.7, 111.1, 62.0 (d, *J*_C–P_ = 6.7 Hz), 32.7 (d, *J*_C–P_ = 138.7 Hz), 16.2 (d, *J*_C–P_ = 6.1 Hz); ^31^P{^1^H} NMR (162 MHz, CDCl_3_): δ 25.59. HRMS (MALDI-TOF) calcd for C_14_H_18_NNaO_3_P [M + Na]^+^: 302.0922, found 302.0920.

#### Diethyl (2-(4-Nitrophenyl)allyl)phosphonate (**3ai**)

Yellow oil (239 mg, 80% from **1a**; 180 mg, 60% from **6a**); purification by silica gel chromatography (eluent: EtOAc, *R*_f_ = 0.35); ^1^H NMR (300 MHz, CDCl_3_): δ 8.18 (d, *J* = 8.9 Hz, 2H), 7.63 (d, *J* = 8.9 Hz, 2H), 5.64 (d, *J* = 5.6 Hz, 1H), 5.51 (d, *J* = 5.6 Hz, 1H), 4.07–3.97 (m, 4H), 3.05 (d, *J* = 22.1 Hz, 2H), 1.21 (t, *J* = 7.1 Hz, 6H); ^13^C{^1^H} NMR (75 MHz, CDCl_3_): δ 147.0 (d, *J*_C–P_ = 4.3 Hz), 146.9, 137.2 (d, *J*_C–P_ = 10.6 Hz), 127.1, 123.5, 120.6 (d, *J*_C–P_ = 10.9 Hz), 62.1 (d, *J*_C–P_ = 6.9 Hz), 32.8 (d, *J*_C–P_ = 138.8 Hz), 16.2 (d, *J*_C–P_ = 6.2 Hz); ^31^P{^1^H} NMR (162 MHz, CDCl_3_): δ 25.46; HRMS (MALDI-TOF) calcd for C_13_H_19_NO_5_P [M + H]^+^: 300.1001, found 300.0993.

#### *tert*-Butyl 4-(3-(Diethoxyphosphoryl)prop-1-en-2-yl)benzoate (**3aj**)

Colorless oil (280 mg, 79%); purification by silica gel chromatography (eluent: EtOAc, *R*_f_ = 0.48); ^1^H NMR (300 MHz, CDCl_3_): δ 7.93 (d, *J* = 8.5 Hz, 2H), 7.49 (d, *J* = 8.5 Hz, 2H), 5.57 (d, *J* = 5.4 Hz, 1H), 5.41 (d, *J* = 5.4 Hz, 1H), 4.06–3.92 (m, 4H), 3.05 (dd, *J* = 22.1, 1.0 Hz, 2H), 1.57 (s, 9H), 1.19 (t, *J* = 7.1 Hz, 6H); ^13^C{^1^H} NMR (75 MHz, CDCl_3_): δ 165.4, 144.4 (d, *J*_C–P_ = 4.2 Hz), 138.0 (d, *J*_C–P_ = 10.6 Hz), 131.1, 129.3, 126.0, 118.7 (d, *J*_C–P_ = 10.8 Hz), 80.9, 62.0 (d, *J*_C–P_ = 6.7 Hz), 32.7 (d, *J*_C–P_ = 138.4 Hz), 28.1, 16.2 (d, *J*_C–P_ = 6.2 Hz); ^31^P{^1^H} NMR (162 MHz, CDCl_3_): δ 25.93; HRMS (MALDI-TOF) calcd for C_18_H_28_O_5_P [M + H]^+^: 355.1674, found 355.1667.

#### Diethyl (2-(3-(Dimethylamino)phenyl)allyl)phosphonate (**3ak**)

Colorless oil (182 mg, 65% from **1a**; 126 mg, 45% from **6a**); purification by silica gel chromatography (eluent: EtOAc, *R*_f_ = 0.27); ^1^H NMR (300 MHz, CDCl_3_): δ 7.20–7.14 (m, 1H), 6.83–6.77 (m, 2H), 6.67–6.63 (m, 1H), 5.48 (dd, *J* = 5.6, 1.1 Hz, 1H), 5.31 (dd, *J* = 5.6, 1.1 Hz, 1H), 4.06–3.92 (m, 4H), 3.04 (d, *J* = 22.2 Hz, 2H), 2.93 (s, 6H), 1.19 (t, *J* = 7.1 Hz, 6H); ^13^C{^1^H} NMR (75 MHz, CDCl_3_): δ 150.6, 141.6, 139.3 (d, *J*_C–P_ = 10.4 Hz), 128.8, 116.8 (d, *J*_C–P_ = 10.8 Hz), 114.8, 112.1, 110.7, 62.0 (d, *J*_C–P_ = 6.7 Hz), 40.7, 33.0 (d, *J*_C–P_ = 137.7 Hz), 16.2 (d, *J*_C–P_ = 6.2 Hz); ^31^P{^1^H} NMR (162 MHz, CDCl_3_): δ 26.74; HRMS (MALDI-TOF) calcd for C_15_H_25_NO_3_P [M + H]^+^: 298.1572, found 298.1564.

#### Diethyl (2-(3-Methoxyphenyl)allyl)phosphonate (**3al**)^3b^

Colorless oil (225 mg, 79%); purification by silica gel chromatography (eluent: EtOAc, *R*_f_ = 0.27); ^1^H NMR (300 MHz, CDCl_3_): δ 7.25–7.20 (m, 1H), 7.04–7.00 (m, 2H), 6.81 (dd, *J* = 8.1, 2.6 Hz, 1H), 5.50 (d, *J* = 5.6 Hz, 1H), 5.33 (d, *J* = 5.6 Hz, 1H), 4.06–3.95 (m, 4H), 3.80 (s, 3H), 3.02 (d, *J* = 22.2 Hz, 2H), 1.19 (t, *J* = 7.1 Hz, 6H); ^13^C{^1^H} NMR (75 MHz, CDCl_3_): δ 159.4, 142.1(d, *J*_C–P_ = 4.3 Hz), 138.4 (d, *J*_C–P_ = 10.3 Hz), 129.2, 118.7, 117.4 (d, *J*_C–P_ = 10.9 Hz), 113.1, 112.1, 62.1 (d, *J*_C–P_ = 6.6 Hz), 55.2, 32.9 (d, *J*_C–P_ = 138.0 Hz), 16.2 (d, *J*_C–P_ = 6.3 Hz); ^31^P{^1^H} NMR (162 MHz, CDCl_3_): δ 26.55.

#### Diethyl (2-(3,5-Dimethylphenyl)allyl)phosphonate (**3am**)^3b^

Colorless oil (248 mg, 88% from **1a**; 225 mg, 80% from **6a**); purification by silica gel chromatography (eluent: EtOAc, *R*_*f*_ = 0.46); ^1^H NMR (300 MHz, CDCl_3_): δ 7.04 (s, 2H), 6.88 (s, 1H), 5.44 (d, *J* = 5.6 Hz, 1H), 5.28 (d, *J* = 5.6 Hz, 1H), 4.03–3.91 (m, 4H), 3.00 (d, *J* = 22.2 Hz, 2H), 2.27 (s, 6H), 1.17 (t, *J* = 7.1 Hz, 6H); ^13^C{^1^H} NMR (75 MHz, CDCl_3_): δ 140.6 (d, *J*_C–P_ = 4.3 Hz), 138.6 (d, *J*_C–P_ = 10.3 Hz), 137.5, 129.2, 124.0, 116.7 (d, *J*_C–P_ = 10.8 Hz), 61.9 (d, *J*_C–P_ = 6.6 Hz), 32.8 (d, *J*_C–P_ = 137.8 Hz), 21.2, 16.1 (d, *J*_C–P_ = 6.3 Hz); ^31^P{^1^H} NMR (162 MHz, CDCl_3_): δ 26.56.

#### Diethyl (2-(3,4-Dimethoxyphenyl)allyl)phosphonate (**3an**)

Colorless oil (189 mg, 60%); purification by silica gel chromatography (eluent: EtOAc, *R*_*f*_ = 0.25); ^1^H NMR (300 MHz, CDCl_3_): δ 7.02–6.98 (m, 2H), 6.78 (d, *J* = 8.2 Hz, 1H), 5.42 (d, *J* = 5.6 Hz, 1H), 5.22 (d, *J* = 5.6 Hz, 1H), 4.04–3.91 (m, 4H), 3.86 (s, 3H), 3.83 (s, 3H), 3.00 (d, *J* = 22.2 Hz, 2H), 1.18 (t, *J* = 7.1 Hz, 6H); ^13^C{^1^H} NMR (75 MHz, CDCl_3_): δ 148.7 (d, *J*_C–P_ = 16.5 Hz), 138.1 (d, *J*_C–P_ = 10.5 Hz), 133.5 (d, *J*_C–P_ = 4.1 Hz), 118.8, 115.8 (d, *J*_C–P_ = 10.8 Hz), 110.7, 109.6, 62.1 (d, *J*_C–P_ = 6.7 Hz), 55.9, 33.1 (d, *J*_C–P_ = 137.8 Hz), 16.3 (d, *J*_C–P_ = 6.1 Hz); ^31^P{^1^H} NMR (162 MHz, CDCl_3_): δ 26.80; HRMS (MALDI-TOF) calcd for C_15_H_24_O_5_P [M + H]^+^: 315.1361, found 315.1355.

#### Diethyl (2-(Naphthalen-2-yl)allyl)phosphonate (**3ao**)

Colorless oil (289 mg, 95% from **1a**; 268 mg, 88% from **6a**); purification by silica gel chromatography (eluent: EtOAc, *R*_*f*_ = 0.40); ^1^H NMR (400 MHz, CDCl_3_): δ 7.89 (s, 1H), 7.83–7.76 (m, 3H), 7.61 (d, *J* = 8.6 Hz, 1H), 7.47–7.41 (m, 2H), 5.66 (d, *J* = 5.5 Hz, 1H), 5.44 (d, *J* = 5.5 Hz, 1H), 4.06–3.91 (m, 4H), 3.16 (d, *J* = 22.2 Hz, 2H), 1.16 (t, *J* = 7.1 Hz, 6H); ^13^C{^1^H} NMR (100 MHz, CDCl_3_): δ 138.4 (d, *J*_C–P_ = 10.3 Hz), 137.7 (d, *J*_C–P_ = 4.3 Hz), 133.1, 132.8, 128.2, 127.8, 127.4, 126.2, 126.0, 125.1, 124.4, 117.6 (d, *J*_C–P_ = 10.7 Hz), 62.0 (d, *J*_C–P_ = 6.6 Hz), 32.9 (d, *J*_C–P_ = 138.2 Hz), 16.2 (d, *J*_C–P_ = 6.2 Hz); ^31^P{^1^H} NMR (162 MHz, CDCl_3_): δ 26.41; HRMS (MALDI-TOF) calcd for C_17_H_22_O_3_P [M + H]^+^: 305.1307, found: 305.1307.

#### Diethyl (2-(Naphthalen-1-yl)allyl)phosphonate (**3ap**)

Colorless oil (180 mg, 59%); purification by silica gel chromatography (eluent: EtOAc, *R*_f_ = 0.44); ^1^H NMR (300 MHz, CDCl_3_): δ 8.03–7.99 (m, 1H), 7.83–7.73 (m, 2H), 7.47–7.39 (m, 4H), 5.68 (dd, *J* = 5.2, 1.3 Hz, 1H), 5.33 (dd, *J* = 5.2, 1.3 Hz, 1H), 3.93–3.84 (m, 4H), 3.10 (dd, *J* = 22.1, 0.9 Hz, 2H), 1.07 (t, *J* = 7.1 Hz, 6H); ^13^C{^1^H} NMR (75 MHz, CDCl_3_): δ 140.2 (d, *J*_C–P_ = 4.3 Hz), 138.4 (d, *J*_C–P_ = 10.4 Hz), 133.6, 130.7, 128.3, 127.6, 125.9, 125.6, 125.5 (d, *J*_C–P_ = 1.5 Hz), 125.4, 125.1, 120.6 (d, *J*_C–P_ = 11.0 Hz), 61.7 (d, *J*_C–P_ = 6.5 Hz), 35.3 (d, *J*_C–P_ = 136.8 Hz), 16.1 (d, *J*_C–P_ = 6.3 Hz); ^31^P{^1^H} NMR (162 MHz, CDCl_3_): δ 26.03; HRMS (MALDI-TOF) calcd for C_17_H_22_O_3_P [M + H]^+^: 305.1307, found: 305.1303.

#### Diethyl (2-(2-Methoxyphenyl)allyl)phosphonate (**3aq**)^3b^

Colorless oil (168 mg, 59%); purification by silica gel chromatography (eluent: EtOAc, *R*_f_ = 0.39); ^1^H NMR (300 MHz, CDCl_3_): δ 7.27–7.21 (m, 2H), 6.92–6.90 (m, 1H), 6.83 (d, *J* = 8.1 Hz, 1H), 5.33 (dd, *J* = 5.0, 1.3 Hz, 1H), 5.21 (dd, *J* = 5.0, 1.3 Hz, 1H), 3.95–3.85 (m, 4H), 3.81 (s, 3H), 3.15 (d, *J* = 21.5 Hz, 2H), 1.13 (t, *J* = 7.1 Hz, 6H); ^13^C{^1^H} NMR (75 MHz, CDCl_3_): δ 156.3, 139.1 (d, *J*_C–P_ = 10.3 Hz), 130.8 (d, *J*_C–P_ = 3.8 Hz), 130.5, 128.9, 120.6, 119.3 (d, *J*_C–P_ = 11.5 Hz), 110.3, 61.6 (d, *J*_C–P_ = 6.5 Hz), 55.3, 33.2 (d, *J*_C–P_ = 136.1 Hz), 16.2 (d, *J*_C–P_ = 6.4 Hz); ^31^P{^1^H} NMR (162 MHz, CDCl_3_): δ 27.36.

### General Procedure for the Synthesis of ((2-Arylallyl)sulfonyl)benzenes (**5**)

A 20 mL sealable glass reactor equipped with a magnetic stirrer bar was charged with arylboronic acid (2 mmol, 2.0 equiv), K_3_PO_4_ (318 mg, 1.5 mmol, 1.5 equiv), **4a** or **7a** (261 mg for **4a** or 217 mg for **7a**, 1.0 mmol, 1.0 equiv), and 2.5 mL of H_2_O. After the addition of NiSO_4_**·**6H_2_O/**L** (13/23 mg, 0.05 mmol, 0.05 equiv, in 1.5 mL of H_2_O), the reaction tube was sealed, and the reaction was stirred at 120 °C (oil bath) for 1 h. After cooling the reaction to room temperature, the aqueous solution was extracted with EtOAc (3 × 4 mL). The combined organic phase was dried over MgSO_4,_ and the solvent was removed under reduced pressure. Column chromatography on silica gel provided the desired products.

#### ((2-Phenylallyl)sulfonyl)benzene (**5aa**)^17^

Pale yellow solid (256 mg, 99% from **4a**; 220 mg, 85% from **7a**); mp 61–62 °C; purification by silica gel chromatography (eluent: hexane/EtOAc = 1:1, *R*_f_ = 0.60); ^1^H NMR (300 MHz, CDCl_3_): δ 7.81–7.79 (m, 2H), 7.59–7.55 (m, 1H), 7.47–7.42 (m, 2H), 7.28–7.25 (m, 5H), 5.61 (s, 1H), 5.24 (s, 1H), 4.29 (s, 2H); ^13^C{^1^H} NMR (75 MHz, CDCl_3_): δ 138.7, 138.3, 136.4, 133.6, 128.9, 128.6, 128.4, 128.0, 126.2, 121.9, 62.0.

#### 1-Methyl-4-(3-(phenylsulfonyl)prop-1-en-2-yl)benzene (**5ab**)^19^

Pale yellow oil (270 mg, 99% from **4a**; 202 mg, 74% from **7a**); purification by silica gel chromatography (eluent: hexane/EtOAc = 1:1, *R*_f_ = 0.62); ^1^H NMR (300 MHz, CDCl_3_): δ 7.78–7.75 (m, 2H), 7.57–7.52 (m, 1H), 7.45–7.40 (m, 2H), 7.15 (d, *J* = 8.2 Hz, 2H), 7.03 (d, *J* = 8.2 Hz, 2H), 5.53 (s, 1H), 5.11 (s, 1H), 4.24 (s, 2H), 2.29 (s, 3H); ^13^C{^1^H} NMR (75 MHz, CDCl_3_): δ 138.2, 137.8, 136.1, 135.7, 133.5, 129.0, 128.8, 128.5, 126.0, 120.9, 61.9, 21.0.

#### 1-(*tert*-Butyl)-4-(3-(phenylsulfonyl)prop-1-en-2-yl)benzene (**5ac**)

Pale yellow oil (236 mg, 75%); purification by silica gel chromatography (eluent: hexane/EtOAc = 1:1, *R*_f_ = 0.64); ^1^H NMR (300 MHz, CDCl_3_): δ 7.76–7.73 (m, 2H), 7.53–7.48 (m, 1H), 7.41–7.36 (m, 2H), 7.23–7.15 (m, 4H), 5.56 (s, 1H), 5.15 (s, 1H), 4.25 (s, 2H), 1.26 (s, 9H); ^13^C{^1^H} NMR (75 MHz, CDCl_3_): δ 151.1, 138.4, 136.2, 135.7, 133.6, 128.9, 128.7, 125.9, 125.3, 121.1, 62.1, 34.5, 31.2; HRMS (MALDI-TOF) calcd for C_19_H_23_O_2_S [M + H]^+^: 315.1419, found: 315.1415.

#### 1-Methoxy-4-(3-(phenylsulfonyl)prop-1-en-2-yl)benzene (**5ad**)^8d^

Pale yellow oil (274 mg, 95% from **4a**; 205 mg, 71% from **7a**); purification by silica gel chromatography (eluent: hexane/EtOAc = 1:1, *R*_f_ = 0.44); ^1^H NMR (300 MHz, CDCl_3_): δ 7.79–7.75 (m, 2H), 7.57–7.53 (m, 1H), 7.45–7.40 (m, 2H), 7.20 (d, *J* = 8.9 Hz, 2H), 6.75 (d, *J* = 8.9 Hz, 2H), 5.48 (s, 1H), 5.06 (s, 1H), 4.23 (s, 2H), 3.77 (s, 3H); ^13^C{^1^H} NMR (75 MHz, CDCl_3_): δ 159.4, 138.2, 135.6, 133.5, 131.0, 128.8, 128.5, 127.3, 120.0, 113.6, 62.1, 55.2.

#### 1-Fluoro-4-(3-(phenylsulfonyl)prop-1-en-2-yl)benzene (**5ae**)^19^

Pale yellow oil (246 mg, 89% from **4a**; 171 mg, 62% from **7a**); purification by silica gel chromatography (eluent: hexane/EtOAc = 2:1, *R*_f_ = 0.38); ^1^H NMR (300 MHz, CDCl_3_): δ 7.78–7.75 (m, 2H), 7.60–7.54 (m, 1H), 7.46–7.41 (m, 2H), 7.25–7.21 (m, 2H), 6.94–6.88 (m, 2H), 5.52 (s, 1H), 5.17 (s, 1H), 4.22 (s, 2H); ^13^C{^1^H} NMR (75 MHz, CDCl_3_): δ 162.4 (d, *J*_C–F_ = 247.8 Hz), 138.2, 135.4, 134.8 (d, *J*_C–F_ = 3.3 Hz), 133.7, 128.9, 128.5, 127.9 (d, *J*_C–F_ = 8.2 Hz), 121.8, 115.2 (d, *J*_C–F_ = 21.6 Hz), 62.1.

#### 1-Chloro-4-(3-(phenylsulfonyl)prop-1-en-2-yl)benzene (**5af**)^8d^

Pale yellow oil (258 mg, 88%); purification by silica gel chromatography (eluent: hexane/EtOAc = 1:1, *R*_f_ = 0.56); ^1^H NMR (300 MHz, CDCl_3_): δ 7.78–7.75 (m, 2H), 7.60–7.55 (m, 1H), 7.47–7.42 (m, 2H), 7.19 (s, 4H), 5.56 (s, 1H), 5.19 (s, 1H), 4.21 (s, 2H); ^13^C{^1^H} NMR (75 MHz, CDCl_3_): δ 138.1, 137.1, 135.3, 133.9, 133.7, 128.9, 128.5, 128.4, 127.5, 122.4, 61.8.

#### 1-(4-(3-(Phenylsulfonyl)prop-1-en-2-yl)phenyl)ethan-1-one (**5ag**)^36^

White solid (273 mg, 91% from **4a**; 210 mg, 70% from **7a**); mp 131–132 °C; purification by silica gel chromatography (eluent: hexane/EtOAc = 1:1, *R*_f_ = 0.39); ^1^H NMR (400 MHz, CDCl_3_): δ 7.82 (d, *J* = 8.4 Hz, 2H), 7.77 (d, *J* = 7.5 Hz, 1H), 7.55 (t, *J* = 7.5 Hz, 2H), 7.43 (t, *J* = 7.5 Hz, 2H), 7.36 (d, *J* = 8.4 Hz, 2H), 5.67 (s, 1H), 5.29 (s, 1H), 4.26 (s, 2H), 2.55 (s, 3H); ^13^C{^1^H} NMR (100 MHz, CDCl_3_): δ 197.4, 143.2, 138.1, 136.3, 135.6, 133.8, 129.0, 128.6, 128.5, 126.4, 123.8, 61.7, 26.6.

#### 4-(3-(Phenylsulfonyl)prop-1-en-2-yl)benzonitrile (**5ah**)

Pale yellow oil (258 mg, 91%); purification by silica gel chromatography (eluent: hexane/EtOAc = 1:1, *R*_f_ = 0.44); ^1^H NMR (400 MHz, CDCl_3_): δ 7.78 (d, *J* = 8.1 Hz, 2H), 7.62–7.58 (m, 1H), 7.54 (d, *J* = 8.4 Hz, 2H), 7.48–7.44 (m, 2H), 7.39 (d, *J* = 8.1 Hz, 2H), 5.69 (s, 1H), 5.34 (s, 1H), 4.23 (s, 2H); ^13^C{^1^H} NMR (100 MHz, CDCl_3_): δ 143.2, 138.0, 135.1, 133.9, 132.2, 129.1, 128.5, 126.9, 124.8, 118.5, 111.6, 61.5; HRMS (MALDI-TOF) calcd for C_16_H_13_NNaO_2_S [M + Na]^+^: 306.0565, found 306.0562.

#### 1-Nitro-4-(3-(phenylsulfonyl)prop-1-en-2-yl)benzene (**5ai**)^36^

Yellowish solid (252 mg, 83% from **4a**; 194 mg, 64% from **7a**); mp 138–139 °C; purification by silica gel chromatography (eluent: hexane/EtOAc = 1:1, *R*_f_ = 0.42); ^1^H NMR (300 MHz, CDCl_3_): δ 8.11 (d, *J* = 9.0 Hz, 2H), 7.81–7.78 (m, 2H), 7.62–7.57 (m, 1H), 7.50–7.44 (m, 2H), 5.73 (s, 1H), 5.38 (s, 1H), 4.26 (s, 2H); ^13^C{^1^H} NMR (75 MHz, CDCl_3_): δ 147.2, 145.1, 137.9, 134.9, 134.0, 129.1, 128.5, 127.1, 125.4, 123.7, 61.6.

#### *tert*-Butyl 4-(3-(phenylsulfonyl)prop-1-en-2-yl)benzoate (**5aj**)

Pale yellow oil (233 mg, 65%); purification by silica gel chromatography (eluent: hexane/EtOAc = 1:1, *R*_f_ = 0.60); ^1^H NMR (300 MHz, CDCl_3_): δ 7.85 (d, *J* = 8.4 Hz, 2H), 7.77 (d, *J* = 8.4 Hz, 2H), 7.59–7.54 (m, 1H), 7.46–7.41 (m, 2H), 7.29 (d, *J* = 7.5 Hz, 2H), 5.65 (s, 1H), 5.27 (s, 1H), 4.25 (s, 2H), 1.57 (s, 9H); ^13^C{^1^H} NMR (75 MHz, CDCl_3_): δ 165.2, 142.5, 138.0, 135.7, 133.7, 131.3, 129.4, 128.9, 128.4, 125.9, 123.4, 81.0, 61.6, 28.0; HRMS (MALDI-TOF) calcd for C_20_H_22_NaO_4_S [M + Na]^+^: 381.1136, found 381.1132.

#### *N*,*N*-Dimethyl-3-(3-(phenylsulfonyl)prop-1-en-2-yl)aniline (**5ak**)

Pale yellow oil (166 mg, 55% from **4a**; 136 mg, 45% from **7a**); purification by silica gel chromatography (eluent: hexane/EtOAc = 1:1, *R*_f_ = 0.49); ^1^H NMR (300 MHz, CDCl_3_): δ 7.79–7.76 (m, 2H), 7.55–7.50 (m, 1H), 7.43–7.38 (m, 2H), 7.10–7.04 (m, 1H), 6.61–6.55 (m, 3H), 5.56 (s, 1H), 5.19 (s, 1H), 4.25 (s, 2H), 2.89 (s, 6H); ^13^C{^1^H} NMR (75 MHz, CDCl_3_): δ 150.4, 139.7, 138.4, 137.2, 133.5, 128.9, 128.8, 128.6, 121.3, 114.6, 112.3, 110.4, 62.3, 40.5; HRMS (MALDI-TOF) calcd for C_17_H_20_NO_2_S [M + H]^+^: 302.1215, found: 302.1209.

#### 1-Methoxy-3-(3-(phenylsulfonyl)prop-1-en-2-yl)benzene (**5al**)^17^

Pale yellow oil (260 mg, 90%); purification by silica gel chromatography (eluent: hexane/EtOAc = 1:1, *R*_f_ = 0.51); ^1^H NMR (300 MHz, CDCl_3_): δ 7.78–7.75 (m, 2H), 7.55–7.50 (m, 1H), 7.43–7.38 (m, 2H), 7.15–7.10 (m, 1H), 6.83–6.72 (m, 3H), 5.57 (s, 1H), 5.20 (s, 1H), 4.23 (s, 2H), 3.73 (s, 3H); ^13^C{^1^H} NMR (75 MHz, CDCl_3_): δ 159.4, 140.1, 138.2, 136.2, 133.6, 129.3, 128.8, 128.5, 122.0, 118.6, 113.3, 112.0, 62.0, 55.1.

#### 1,3-Dimethyl-5-(3-(phenylsulfonyl)prop-1-en-2-yl)benzene (**5am**)

White solid (201 mg, 70% from **4a**; 158 mg, 55% from **7a**); purification by silica gel chromatography (eluent: hexane/EtOAc = 2:1, *R*_*f*_ = 0.51); mp 79–81 °C; ^1^H NMR (300 MHz, CDCl_3_): δ 7.77–7.74 (m, 2H), 7.56–7.51 (m, 1H), 7.43–7.38 (m, 2H), 6.83 (s, 1H), 6.77 (s, 2H), 5.54 (s, 1H), 5.18 (s, 1H), 4.23 (s, 2H), 2.21 (s, 3H), 2.20 (s, 3H); ^13^C{^1^H} NMR (75 MHz, CDCl_3_): δ 138.6, 138.3, 137.7, 136.5, 133.5, 129.6, 128.7, 128.6, 124.0, 121.3, 62.1, 21.2; HRMS (MALDI-TOF) calcd for C_17_H_19_O_2_S [M + H]^+^: 287.1106, found 287.1110.

#### 1,2-Dimethoxy-4-(3-(phenylsulfonyl)prop-1-en-2-yl)benzene (**5an**)^8d^

Pale yellow oil (277 mg, 87%); purification by silica gel chromatography (eluent: hexane/EtOAc = 1:1, *R*_f_ = 0.33); ^1^H NMR (300 MHz, CDCl_3_): δ 7.76 (d, *J* = 8.6 Hz, 2H), 7.56–7.51 (m, 1H), 7.43–7.38 (m, 2H), 6.83–6.77 (m, 2H), 6.70 (d, *J* = 8.3 Hz, 1H), 5.49 (s, 1H), 5.09 (s, 1H), 4.22 (s, 2H), 3.82 (s, 3H), 3.80 (s, 3H); ^13^C{^1^H} NMR (75 MHz, CDCl_3_): δ 149.0, 148.5, 138.3, 135.9, 133.5, 131.5, 128.8, 128.6, 120.3, 118.8, 110.7, 109.4, 62.2, 55.8, 55.7.

#### 2-(3-(Phenylsulfonyl)prop-1-en-2-yl)naphthalene (**5ao**)^8d^

White solid (302 mg, 98% from **4a**; 243 mg, 79% from **7a**); mp 128–129 °C; purification by silica gel chromatography (eluent: hexane/EtOAc = 2:1, *R*_f_ = 0.40); ^1^H NMR (300 MHz, CDCl_3_): δ 7.79–7.68 (m, 5H), 7.62 (s, 1H), 7.45–7.33 (m, 6H), 5.72 (s, 1H), 5.31 (s, 1H), 4.37 (s, 2H); ^13^C{^1^H} NMR (75 MHz, CDCl_3_): δ 138.2, 136.2, 135.7, 133.5, 132.9, 132.7, 128.7, 128.5, 128.2, 128.0, 127.4, 126.2, 125.3, 123.9, 122.2, 62.0.

#### 1-(3-(Phenylsulfonyl)prop-1-en-2-yl)naphthalene (**5ap**)

Pale yellow oil (148 mg, 48%); purification by silica gel chromatography (eluent: hexane/EtOAc = 1:1, *R*_f_ = 0.58); ^1^H NMR (300 MHz, CDCl_3_): δ 7.81–7.77 (m, 2H), 7.70–7.66 (m, 3H), 7.46–7.42 (m, 3H), 7.33–7.27 (m, 3H), 7.13–7.10 (m, 1H), 5.74 (d, *J* = 1.1 Hz, 1H), 5.52 (d, *J* = 1.1 Hz, 1H), 4.33 (s, 2H); ^13^C{^1^H} NMR (75 MHz, CDCl_3_): δ 138.7, 137.9, 135.6, 133.6, 133.3, 130.4, 128.7, 128.5, 128.2, 128.1, 126.2, 125.9, 125.8, 125.4, 125.0, 124.8, 63.4; HRMS (MALDI-TOF) calcd for C_19_H_16_NaO_2_S [M + Na]^+^: 331.0769, found 331.0760.

#### 1-Methyl-2-(3-(phenylsulfonyl)prop-1-en-2-yl)benzene (**5ar**)^8d^

Colorless oil (139 mg, 51%); purification by silica gel chromatography (eluent: hexane/EtOAc = 1:1, *R*_f_ = 0.60); ^1^H NMR (300 MHz, CDCl_3_): δ 7.77–7.74 (m, 2H), 7.58–7.53 (m, 1H), 7.45–7.40 (m, 2H), 7.10–7.01 (m, 2H), 6.90 (d, *J* = 7.4 Hz, 2H), 5.50 (d, *J* = 1.1 Hz, 2H), 5.28 (d, *J* = 1.1 Hz, 2H), 4.15 (s, 2H), 2.21 (s, 3H); ^13^C{^1^H} NMR (75 MHz, CDCl_3_): δ 139.8, 138.9, 136.6, 134.6, 133.5, 130.3, 128.9, 128.6, 128.2, 127.7, 125.6, 124.3, 63.1, 20.0.

#### Diisopropyl (2-Phenylallyl)phosphonate (**8**)^3c^

Colorless oil (2.54 g, 90%); purification by silica gel chromatography (eluent: EtOAc, *R*_f_ = 0.37); ^1^H NMR (300 MHz, CDCl_3_): δ 7.46–7.43 (m, 2H), 7.33–7.26 (m, 3H), 5.48 (dd, *J* = 5.6, 1.1 Hz, 1H), 5.33 (dd, *J* = 5.6, 1.1 Hz, 1H), 4.67–4.56 (m, 2H), 3.00 (d, *J* = 22.2 Hz, 2H), 1.23 (d, *J* = 6.2 Hz, 6H), 1.14 (d, *J* = 6.2 Hz, 6H); ^13^C{^1^H} NMR (75 MHz, CDCl_3_): δ 140.8 (d, *J*_C–P_ = 4.4 Hz), 138.9 (d, *J*_C–P_ = 10.4 Hz), 128.1, 127.5, 126.3, 117.0 (d, *J*_C–P_ = 10.9 Hz), 70.4 (d, *J*_C–P_ = 6.9 Hz), 33.9 (d, *J*_C–P_ = 139.4 Hz), 24.0 (d, *J*_C–P_ = 3.7 Hz), 23.7 (d, *J*_C–P_ = 5.2 Hz); ^31^P{^1^H} NMR (162 MHz, CDCl_3_): δ 24.58.

#### 1-Methyl-4-((2-phenylallyl)sulfonyl)benzene (**9**)^17^

White solid (2.37 g, 87%); mp 98–99 °C; purification by silica gel chromatography (eluent: hexane/EtOAc = 1:1, *R*_f_ = 0.60); ^1^H NMR (300 MHz, CDCl_3_): δ 7.63 (d, *J* = 8.3 Hz, 2H), 7.24–7.18 (m, 7H), 5.57 (s, 1H), 5.18 (s, 1H), 4.23 (s, 2H), 2.36 (s, 3H); ^13^C{^1^H} NMR (75 MHz, CDCl_3_): δ 144.5, 138.7, 136.5, 135.3, 129.4, 128.6, 128.3, 127.8, 126.1, 121.7, 62.0, 21.5.

## Data Availability

The data underlying this study are available in the published article and its Supporting Information.

## Supporting Information Available

Copies of ^1^H and ^13^C{^1^H} NMR spectra for **1** and **3**–**9**, and ^31^P{^1^H} NMR spectra for **1**, **3**, **6a**, and **8** (PDF)
