# Supplementary material for: Nickel-Catalyzed Suzuki–Miyaura Coupling in Water for the Synthesis of 2-Aryl Allyl Phosphonates and Sulfones
Source: J Org Chem. 2024 Jan 26;89(4):2448–58. doi: 10.1021/acs.joc.3c02455 (PMC10877605; doi:10.1021/acs.joc.3c02455)

Nickel-Catalyzed Suzuki-Miyaura Coupling in Water for the Synthesis of 2-Aryl Allyl Phosphonates and Sulfones

Yu-Chen Yu,[a,‡] Yun-Chiao Sung,[b,‡] Jun-Hao Fu,[c] Wen-Sheng Peng,[b] Yu-Chia Yu,[b] Juyun Li,[b] Yi-Tsu Chan,[c*] and Fu-Yu Tsai[a,b*]

[a] *Department of Molecular Science and Engineering, National Taipei University of Technology, Taipei 10608, Taiwan*

[b] *Institute of Organic and Polymeric Materials, National Taipei University of Technology, Taipei 10608, Taiwan*

[c] *Department of Chemistry, National Taiwan University, Taipei 10617, Taiwan*

* Corresponding authors: e-mail: ytchan@ntu.edu.tw (Y.-T. Chan); fuyutsai@ntut.edu.tw (F.-Y. Tsai).

‡These authors contributed equally to this paper.

Table of Contents

Copies of NMR spectra for compounds **1** and **3–9** S2–S55

Copies of NMR spectra for compounds 1 and 3–9

^{1}H NMR (300 MHz, CDCl$_3$), ^{13}C{^{1}H} NMR (75 MHz, CDCl$_3$), and ^{31}P{^{1}H} NMR (162 MHz, CDCl$_3$) spectra for diethyl (2-bromoallyl)phosphonate **(1a)**

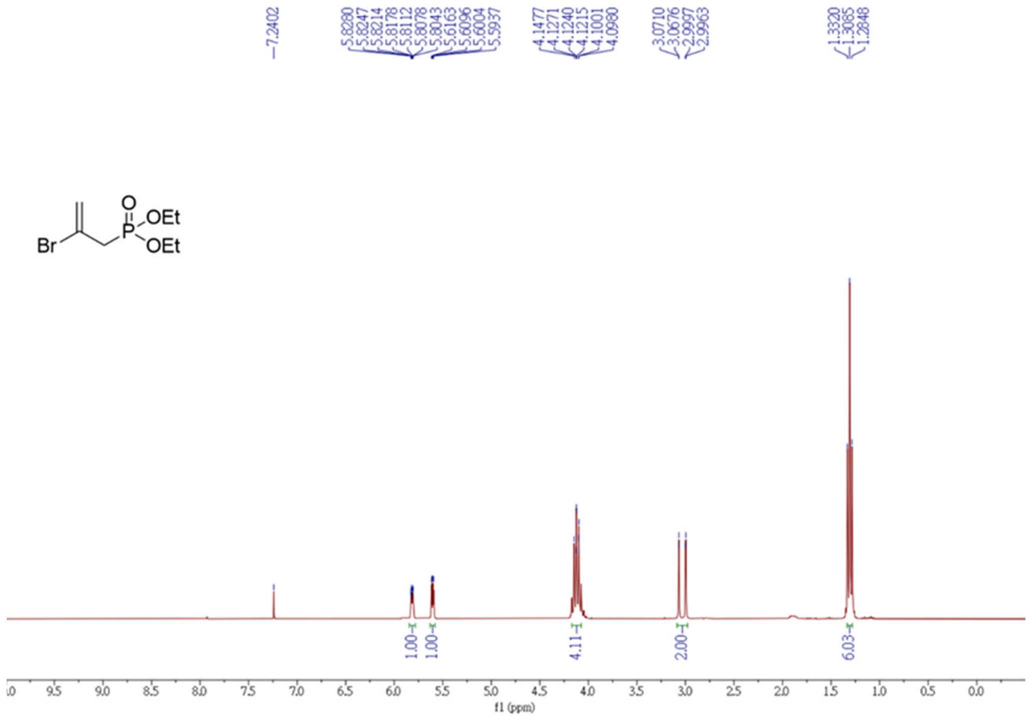

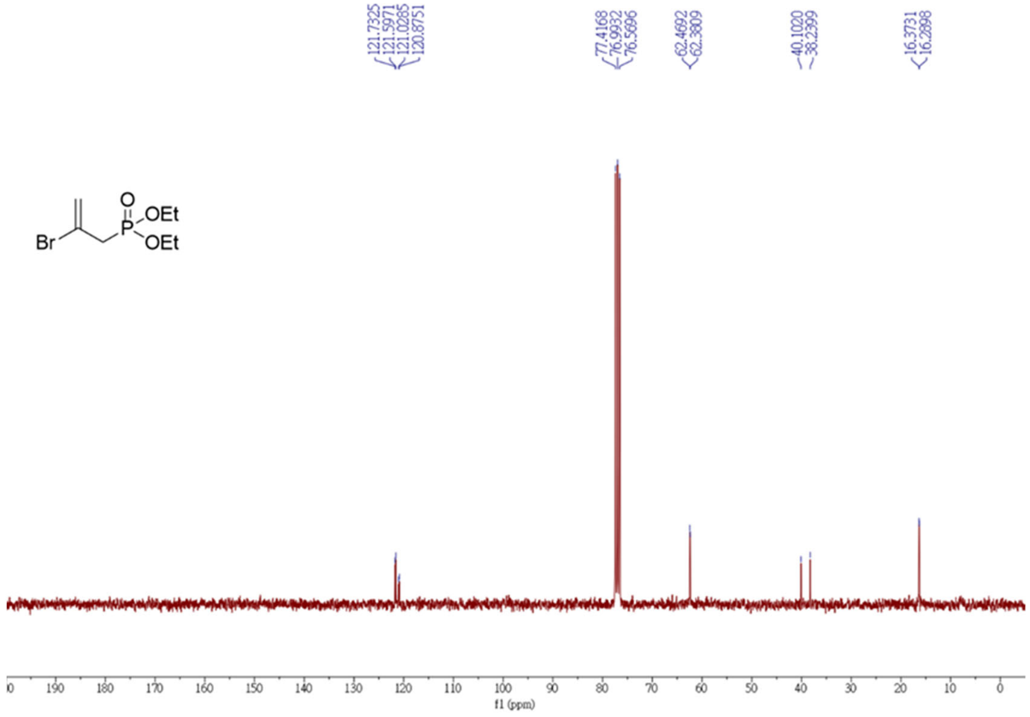

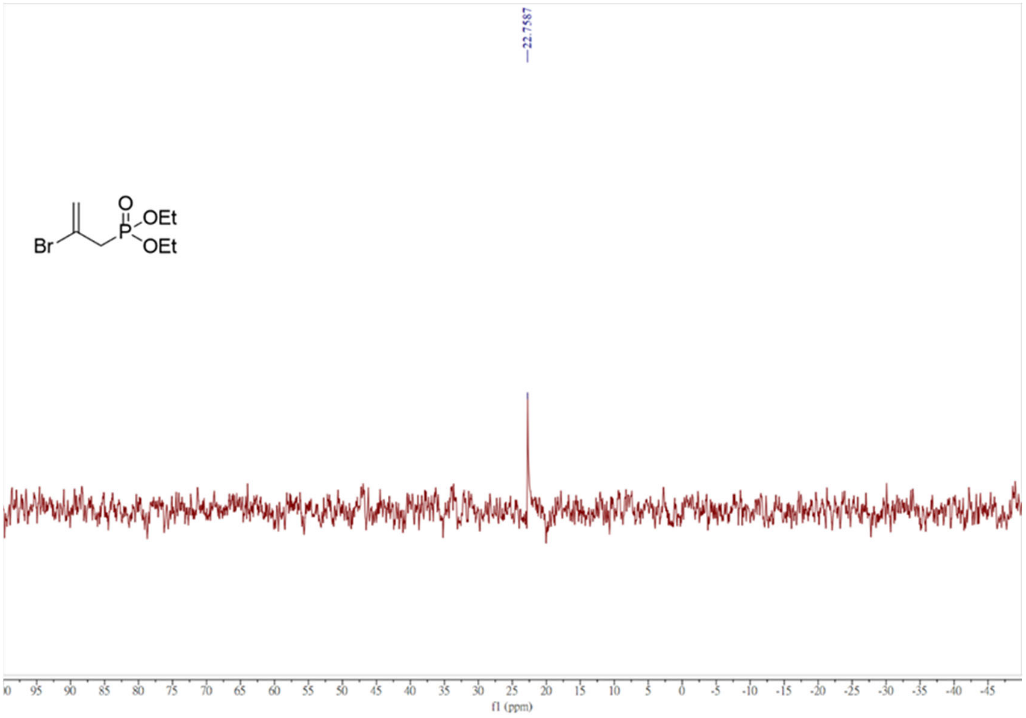

^{1}H NMR (300 MHz, $CDCl_3$), $^{13}C\{^{1}H\}$ NMR (75 MHz, $CDCl_3$), and $^{31}P\{^{1}H\}$ NMR (162 MHz, $CDCl_3$) spectra for diisopropyl (2-bromoallyl)phosphonate **(1b)**

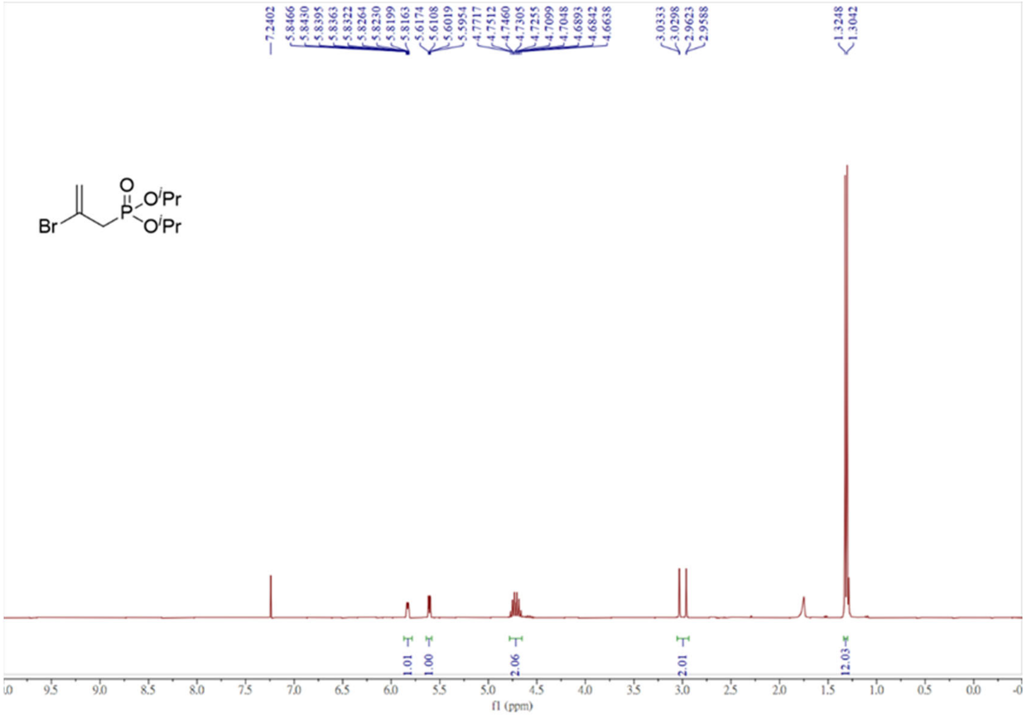

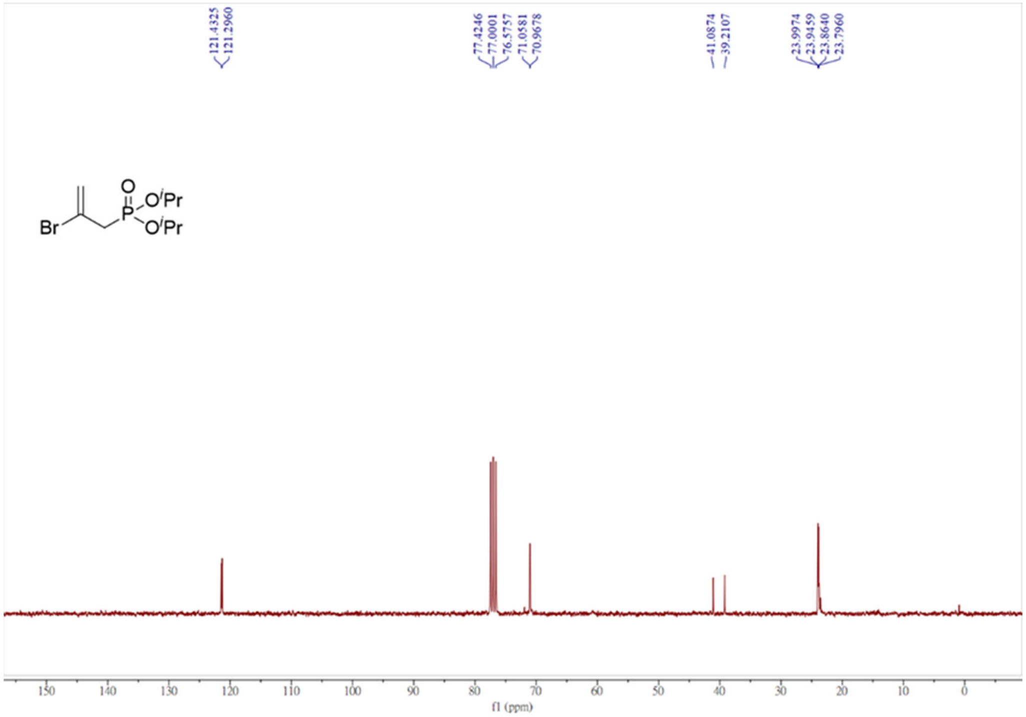
121.4325
121.2960
77.4246
77.0001
76.5757
71.0581
70.9678
41.0874
39.2107
23.9974
23.9459
23.8640
23.7960
Br
O
P
OⁱPr
OⁱPr
f1 (ppm)

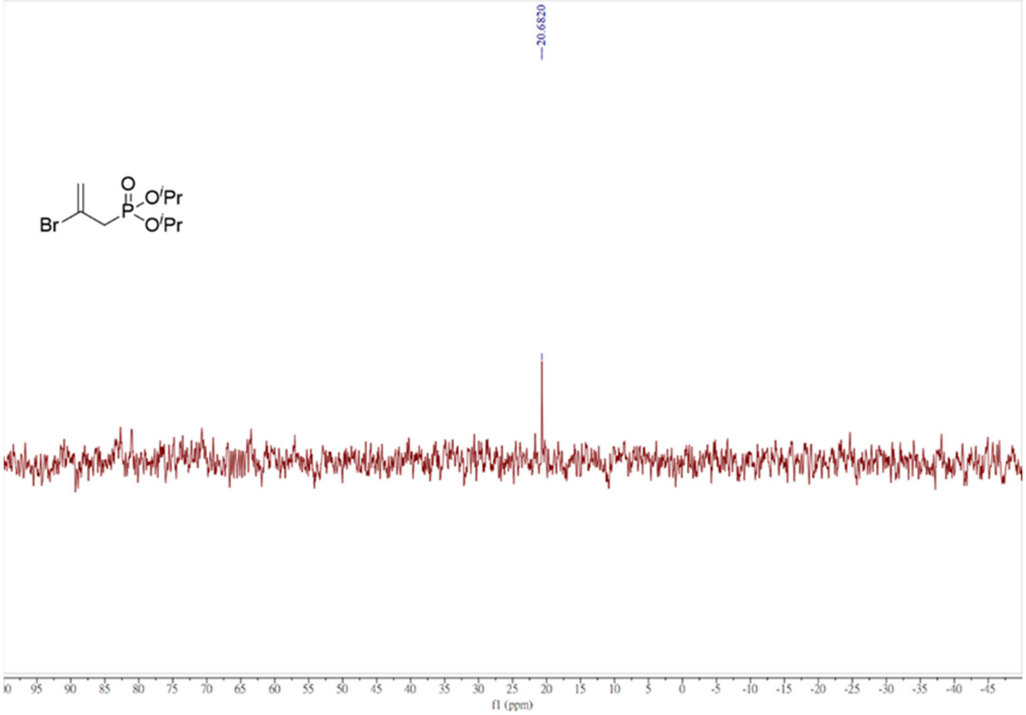
20.6820
Br
O
P
OⁱPr
OⁱPr
f1 (ppm)

^{1}H NMR (300 MHz, $CDCl_3$), $^{13}C\{^{1}H\}$ NMR (75 MHz, $CDCl_3$), and $^{31}P\{^{1}H\}$ NMR (162 MHz, $CDCl_3$) spectra for diethyl (2-phenylallyl)phosphonate **(3aa)**

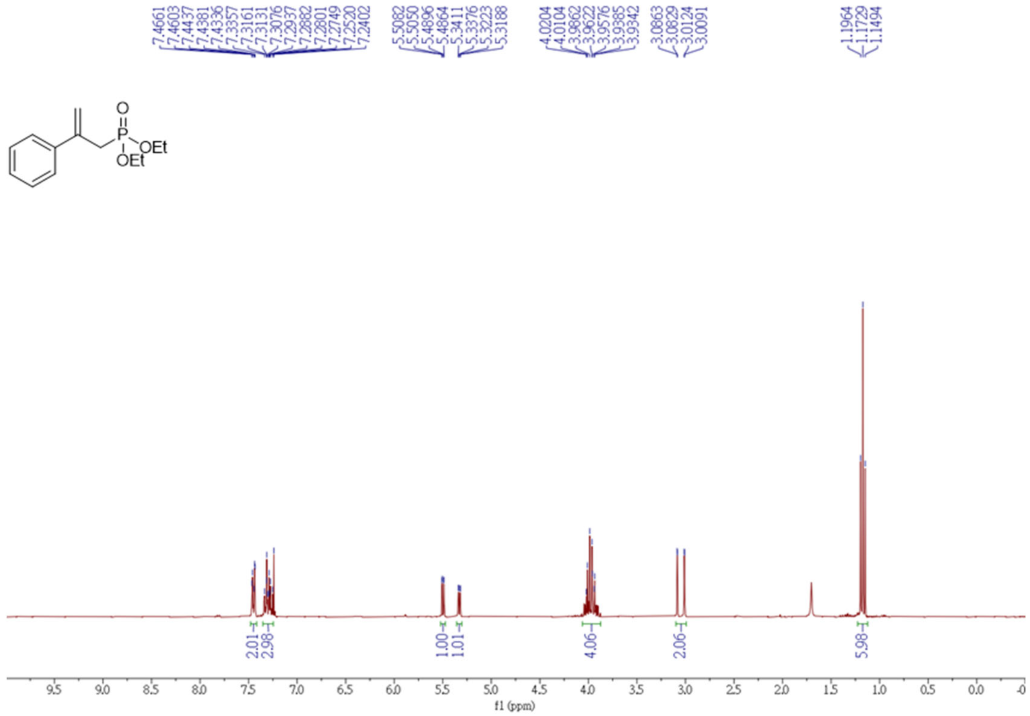

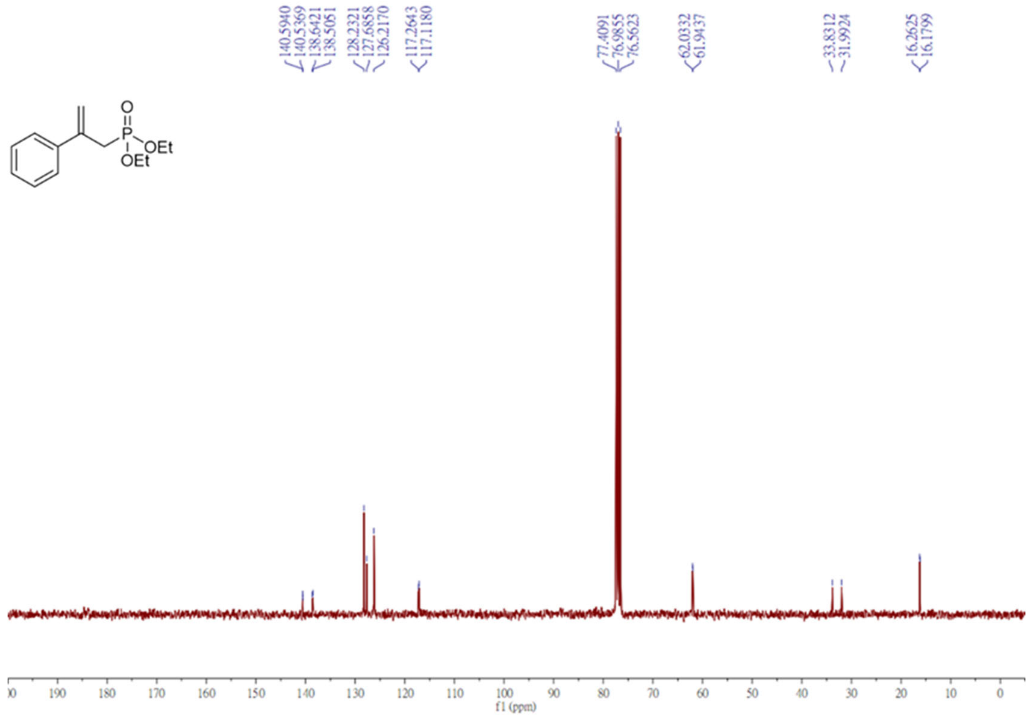

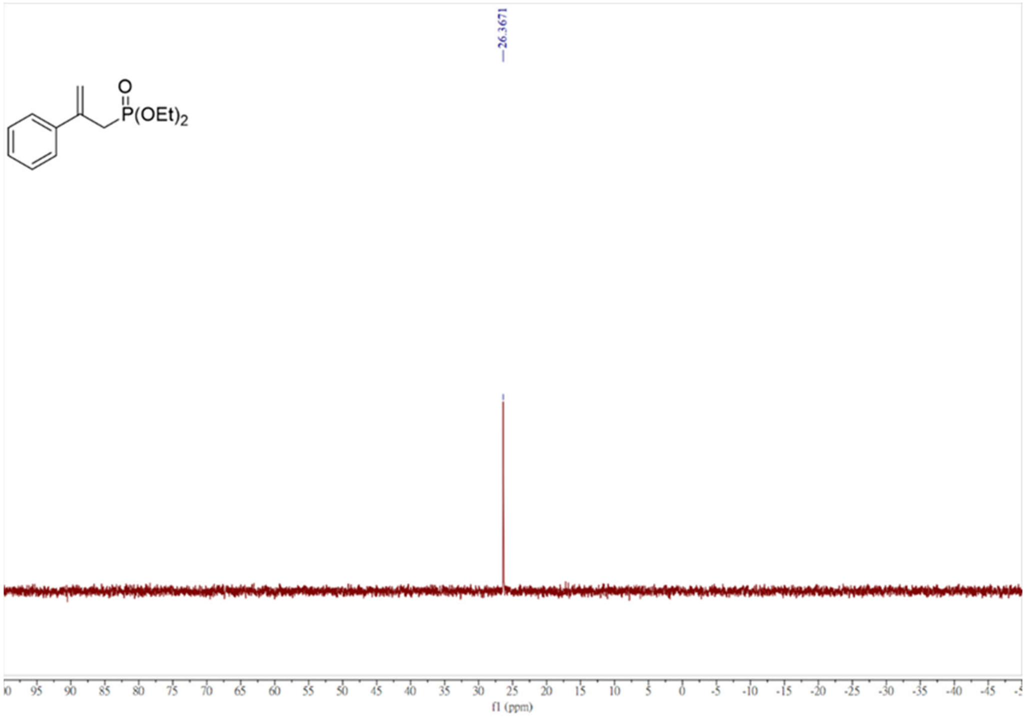

^{1}H NMR (300 MHz, $CDCl_3$), $^{13}C\{^1H\}$ NMR (75 MHz, $CDCl_3$), and $^{31}P\{^1H\}$ NMR (162 MHz, $CDCl_3$) spectra for diethyl (2-(*p*-tolyl)allyl)phosphonate **(3ab)**

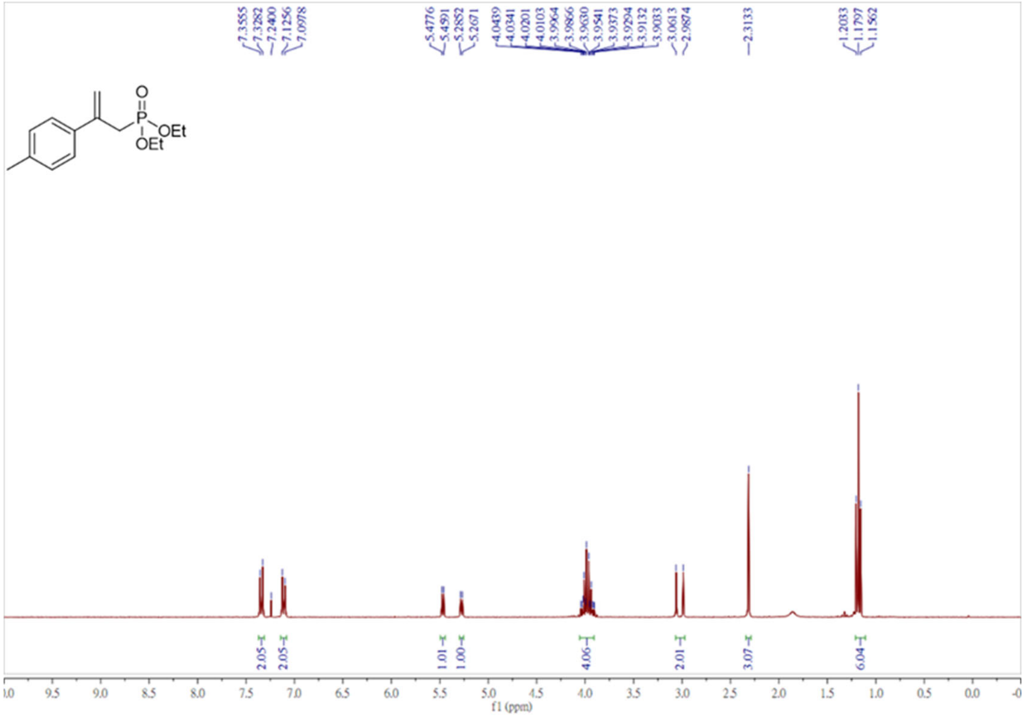

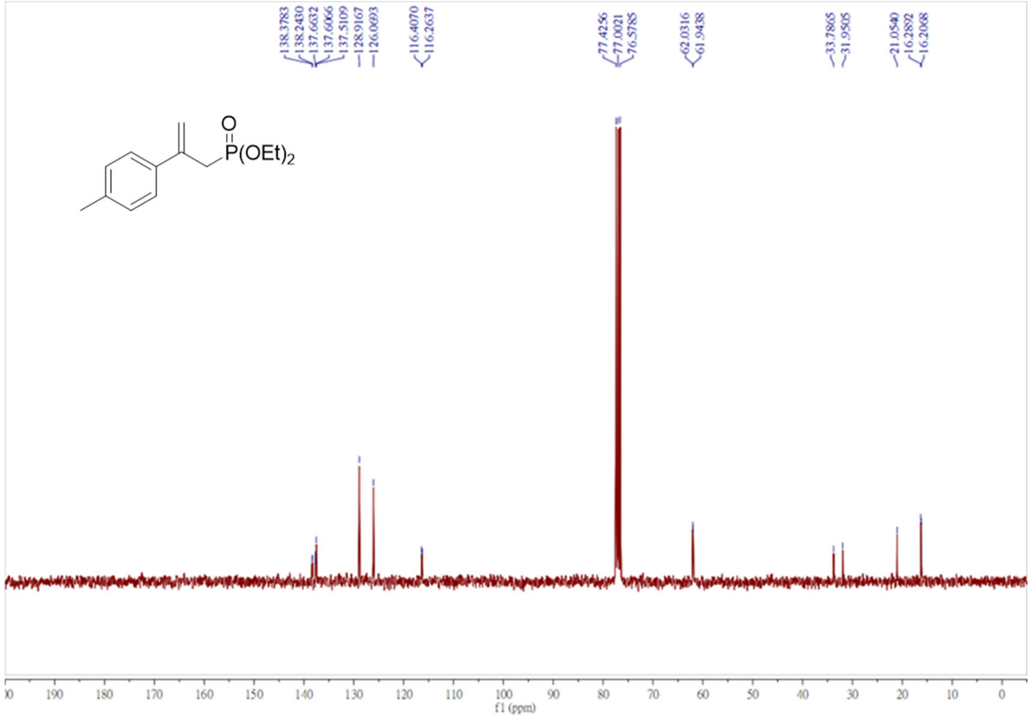
138.3783
138.2430
137.6632
137.6066
137.5109
128.9167
126.0693
116.4070
116.2637
77.4256
77.0021
76.5785
62.0316
61.9438
33.7865
31.9505
21.0540
16.2892
16.2068
P(OEt)2
f1 (ppm)

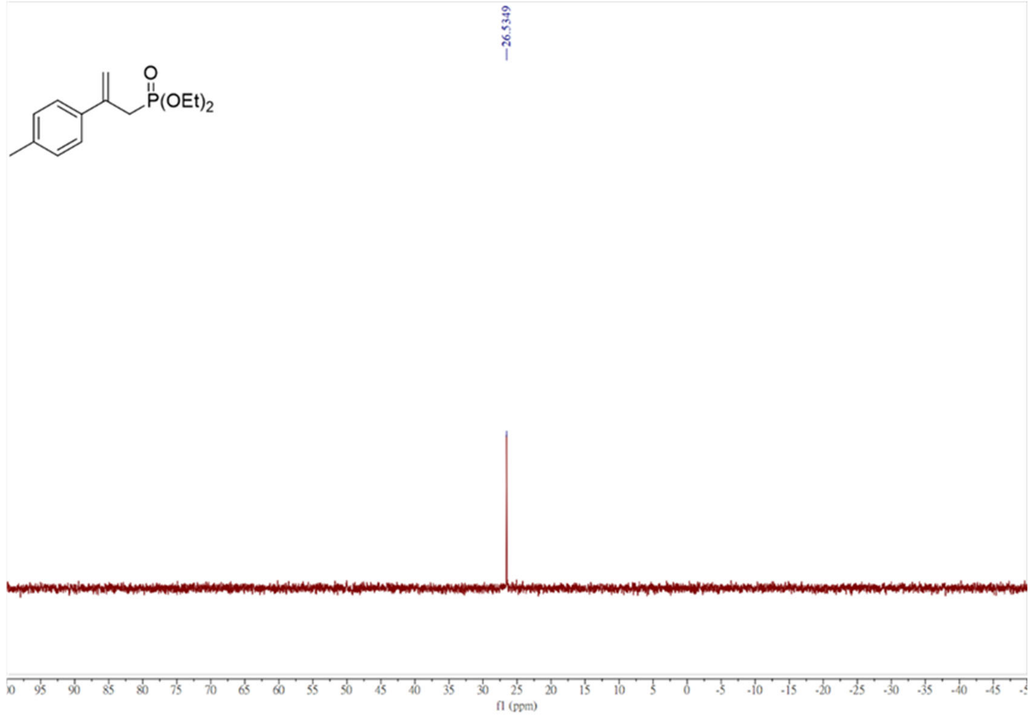
26.5349
P(OEt)2
f1 (ppm)

^{1}H NMR (300 MHz, $CDCl_3$), ^{13}C{^{1}H} NMR (75 MHz, $CDCl_3$), and ^{31}P{^{1}H} NMR (162 MHz, $CDCl_3$) spectra for diethyl (2-(4-(*tert*-butyl)phenyl)allyl)phosphonate **(3ac)**

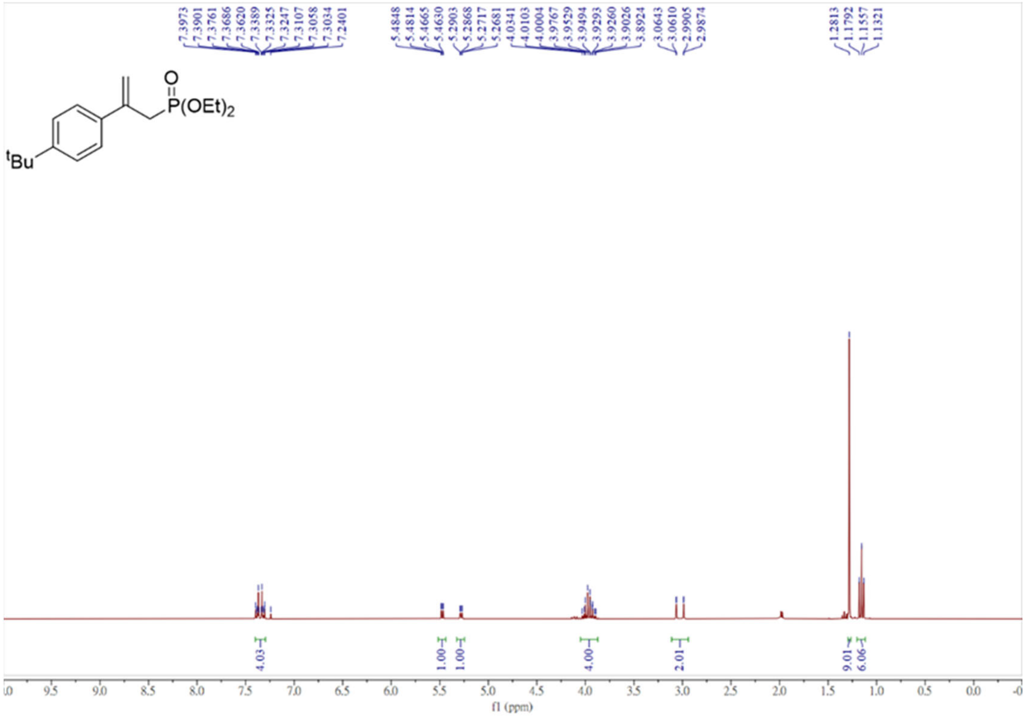

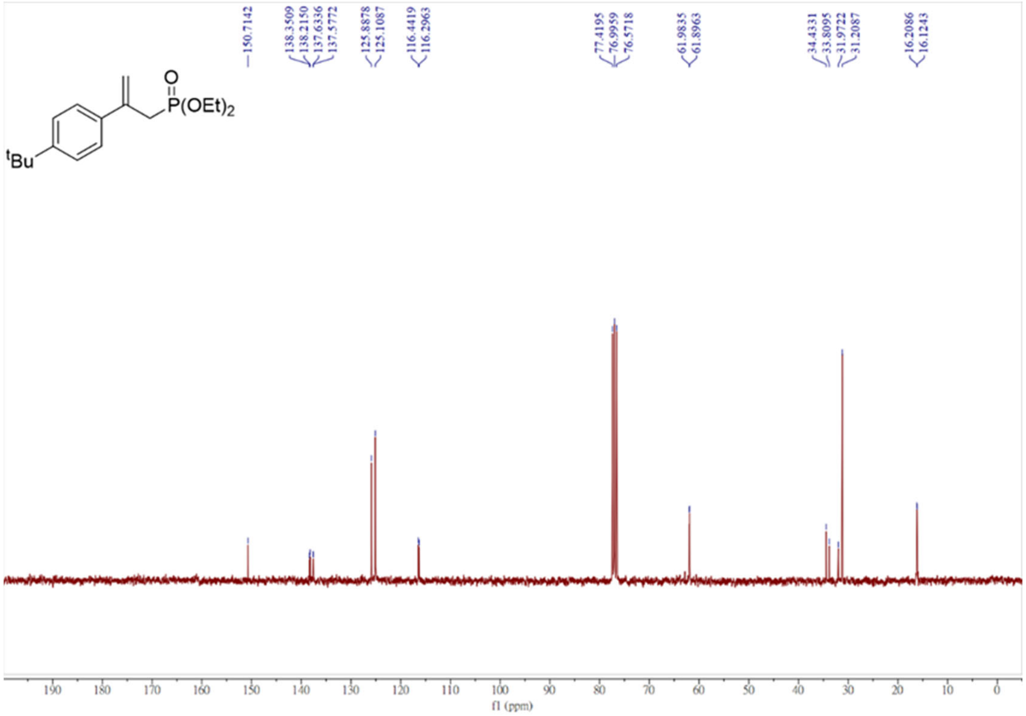

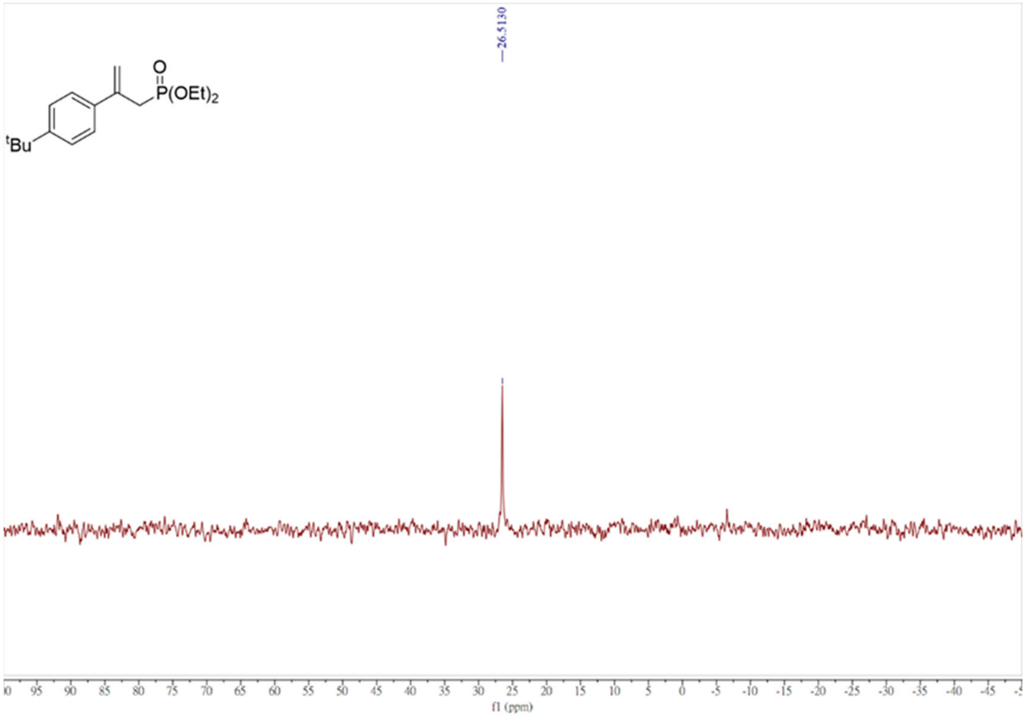

^{1}H NMR (300 MHz, $CDCl_3$), $^{13}C\{^{1}H\}$ NMR (75 MHz, $CDCl_3$), and $^{31}P\{^{1}H\}$ NMR (162 MHz, $CDCl_3$) spectra for diethyl (2-(4-methoxyphenyl)allyl)phosphonate **(3ad)**

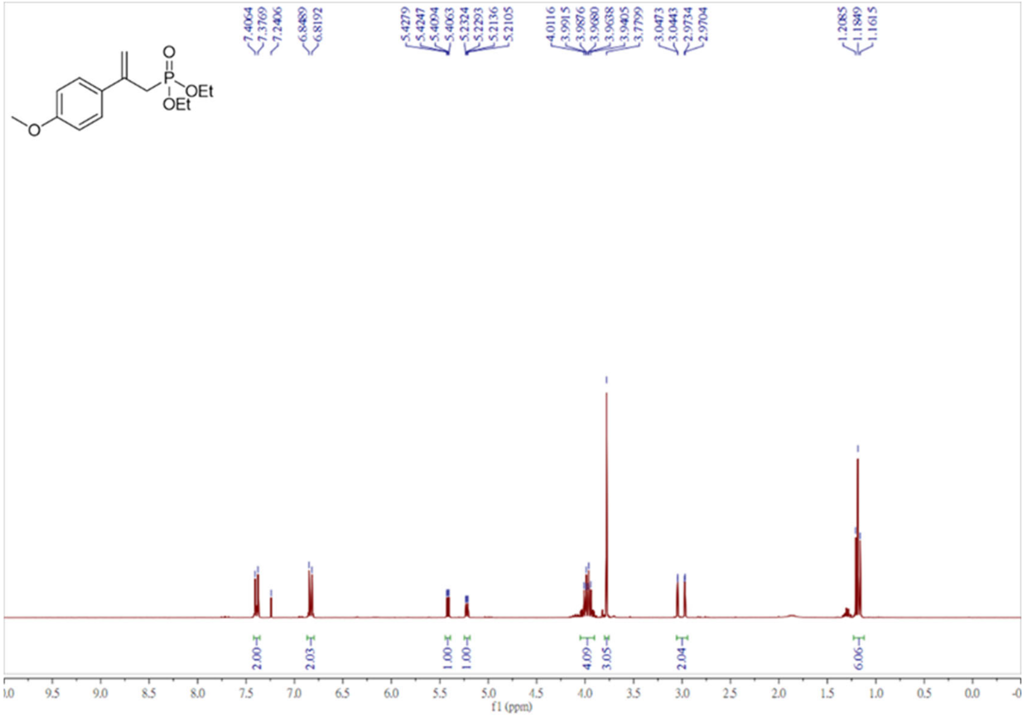

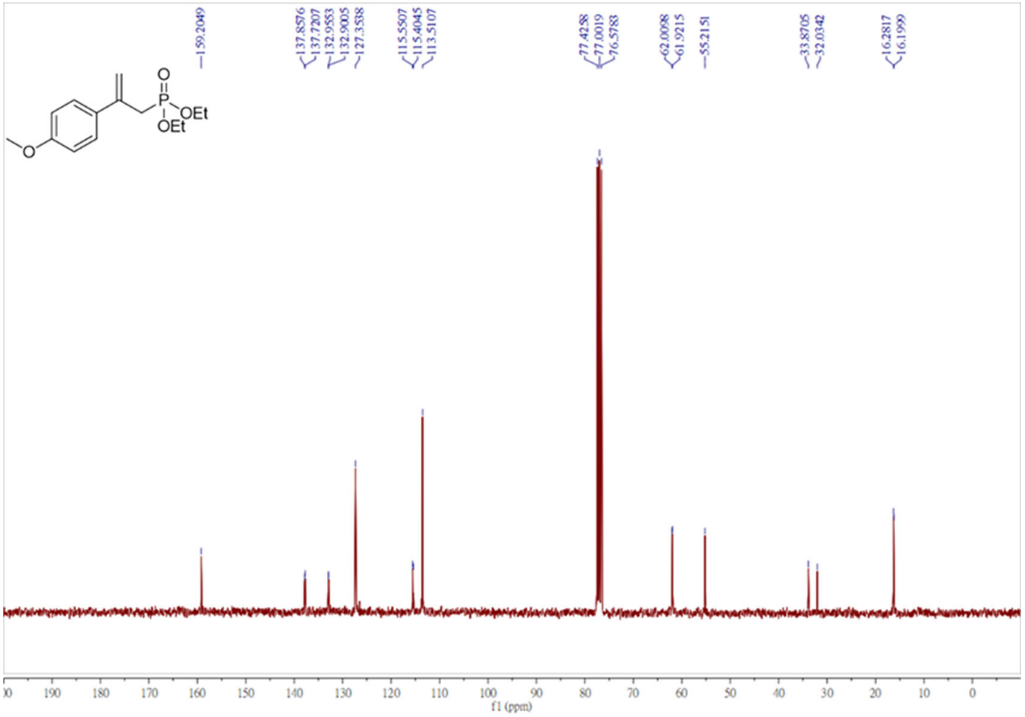
159.2049
137.8576
137.7207
132.9553
132.9005
127.3538
115.5507
115.4045
113.5107
77.4258
77.0019
76.5783
62.0098
61.9215
55.2151
33.8705
32.0342
16.2817
16.1999
OEt
OEt
O
P
O
f1 (ppm)

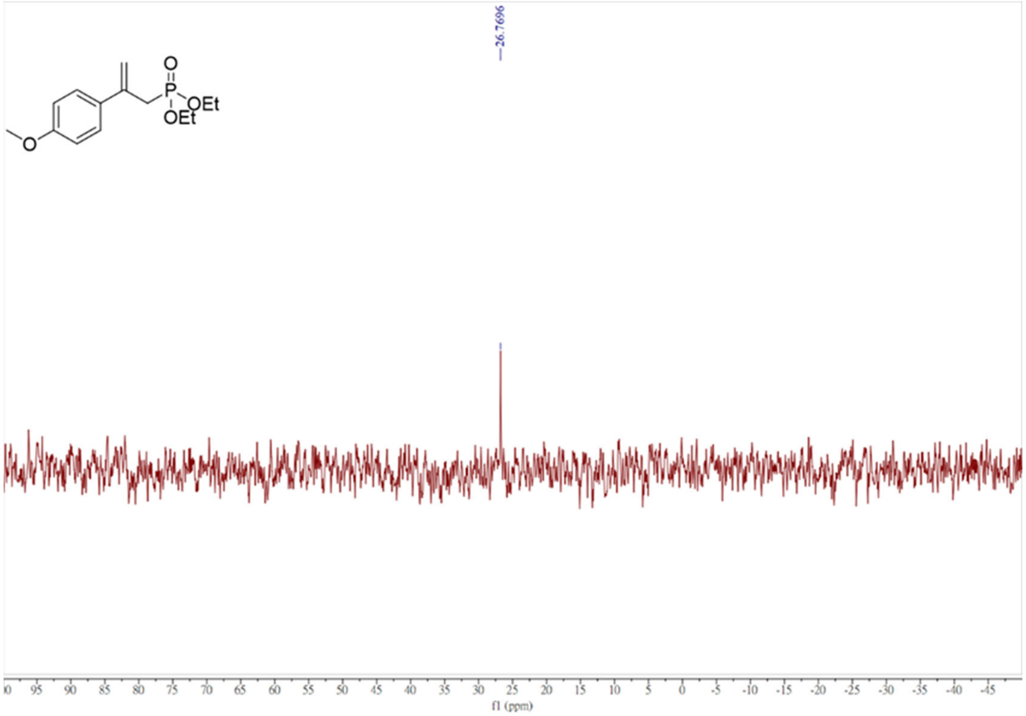
26.7696
OEt
OEt
O
P
O
f1 (ppm)

^{1}H NMR (300 MHz, $CDCl_3$), ^{13}C{^{1}H} NMR (75 MHz, $CDCl_3$), and ^{31}P{^{1}H} NMR (162 MHz, $CDCl_3$) spectra for diethyl (2-(4-fluorophenyl)allyl)phosphonate **(3ae)**

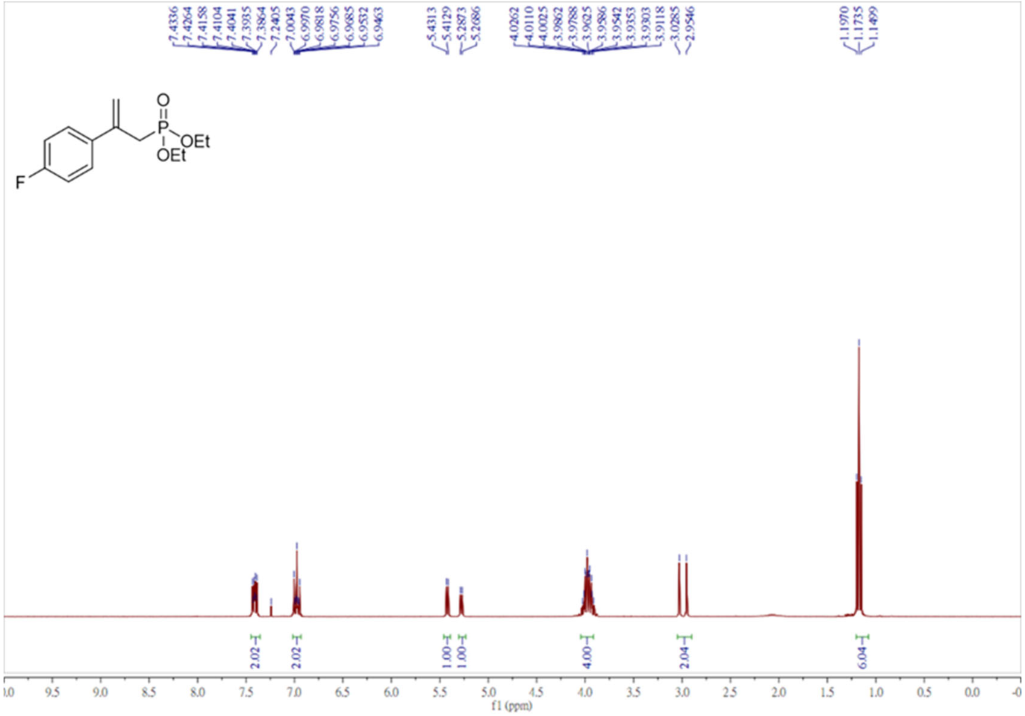

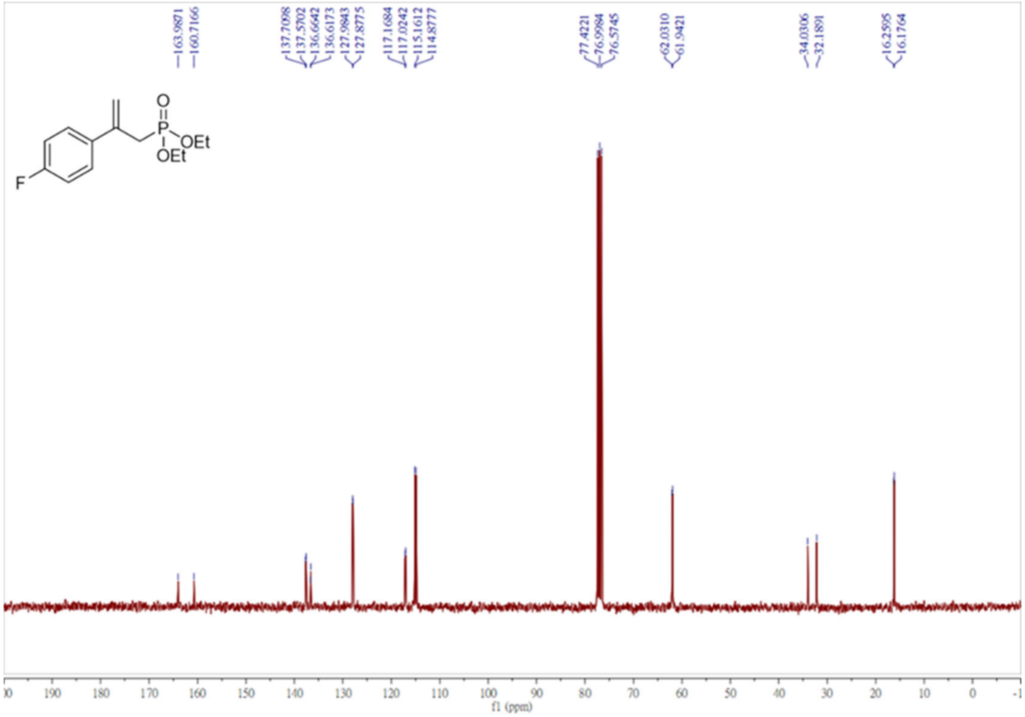

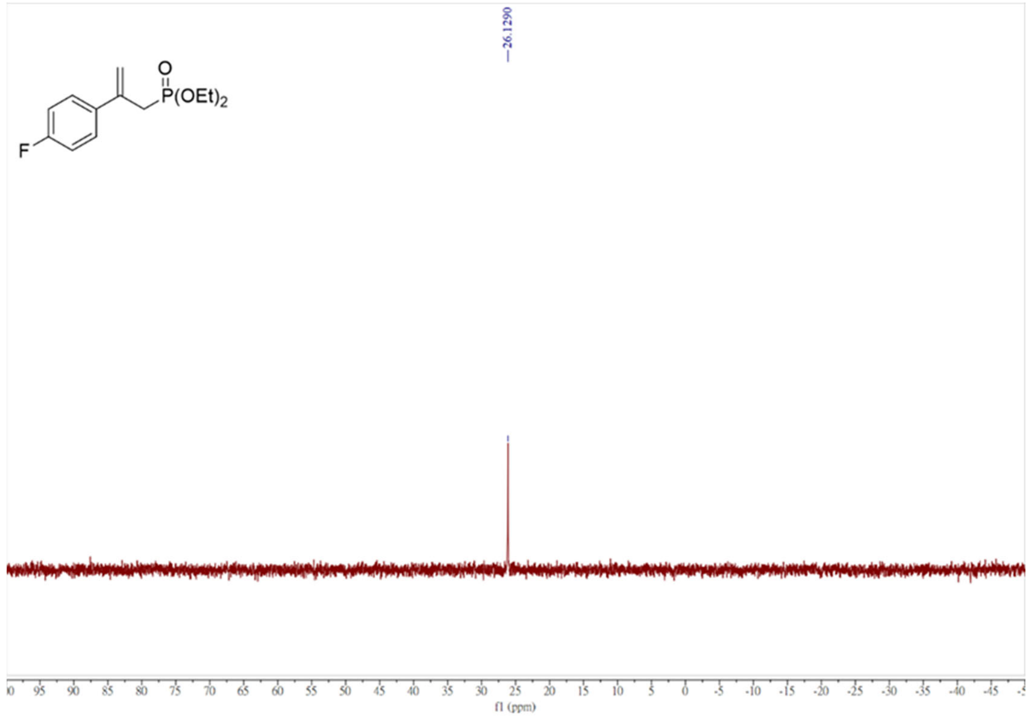

^{1}H NMR (300 MHz, $CDCl_3$), $^{13}C\{^{1}H\}$ NMR (75 MHz, $CDCl_3$), and $^{31}P\{^{1}H\}$ NMR (162 MHz, $CDCl_3$) spectra for diethyl (2-(4-chlorophenyl)allyl)phosphonate **(3af)**

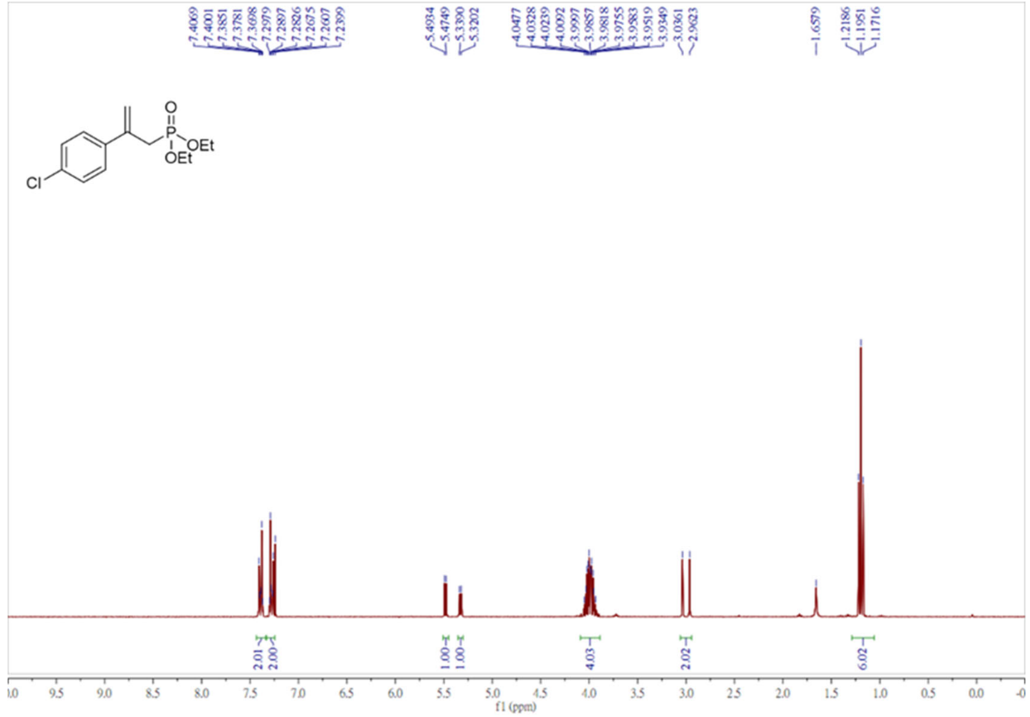

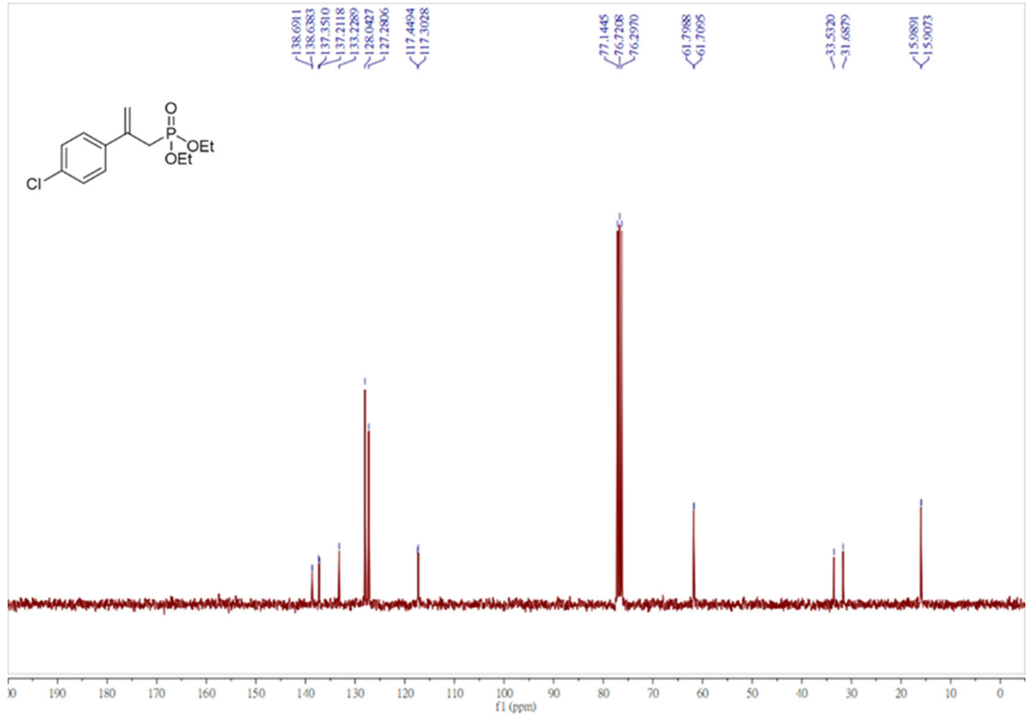
138.6911
138.6383
137.3510
137.2118
133.2289
128.0427
127.2806
117.4494
117.3028
77.1445
76.7208
76.2970
61.7988
61.7095
33.5320
31.6879
15.9891
15.9073
O
P
OEt
OEt
Cl
f1 (ppm)

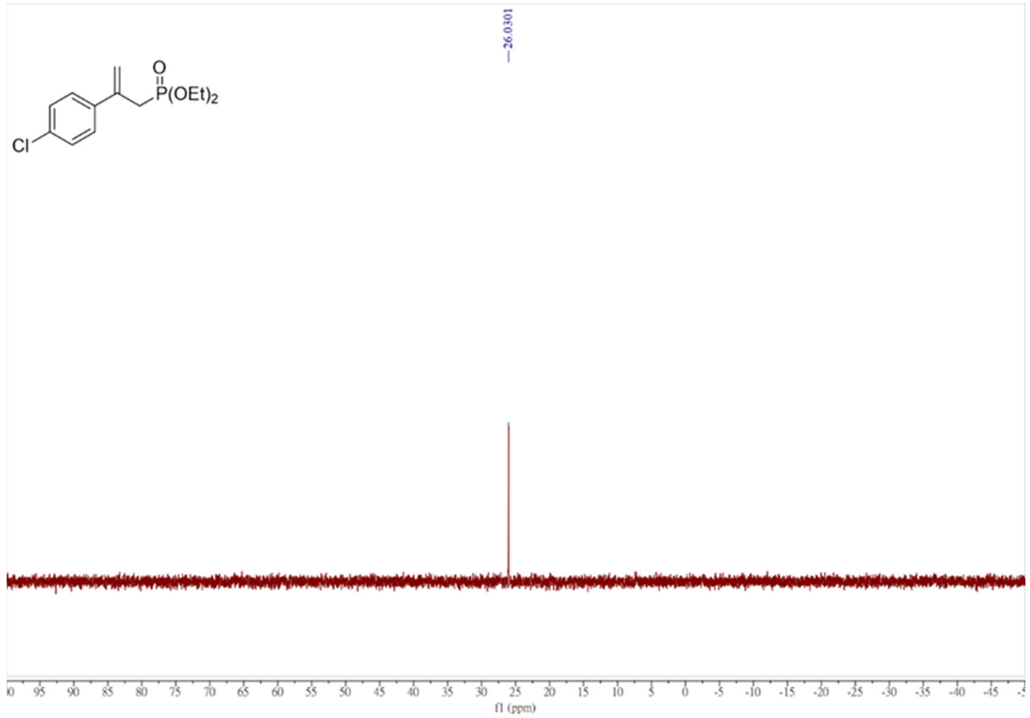
26.0301
O
P(OEt)2
Cl
f1 (ppm)

^{1}H NMR (300 MHz, $CDCl_3$), $^{13}C\{^{1}H\}$ NMR (75 MHz, $CDCl_3$), and $^{31}P\{^{1}H\}$ NMR (162 MHz, $CDCl_3$) spectra for diethyl (2-(4-acetylphenyl)allyl)phosphonate **(3ag)**

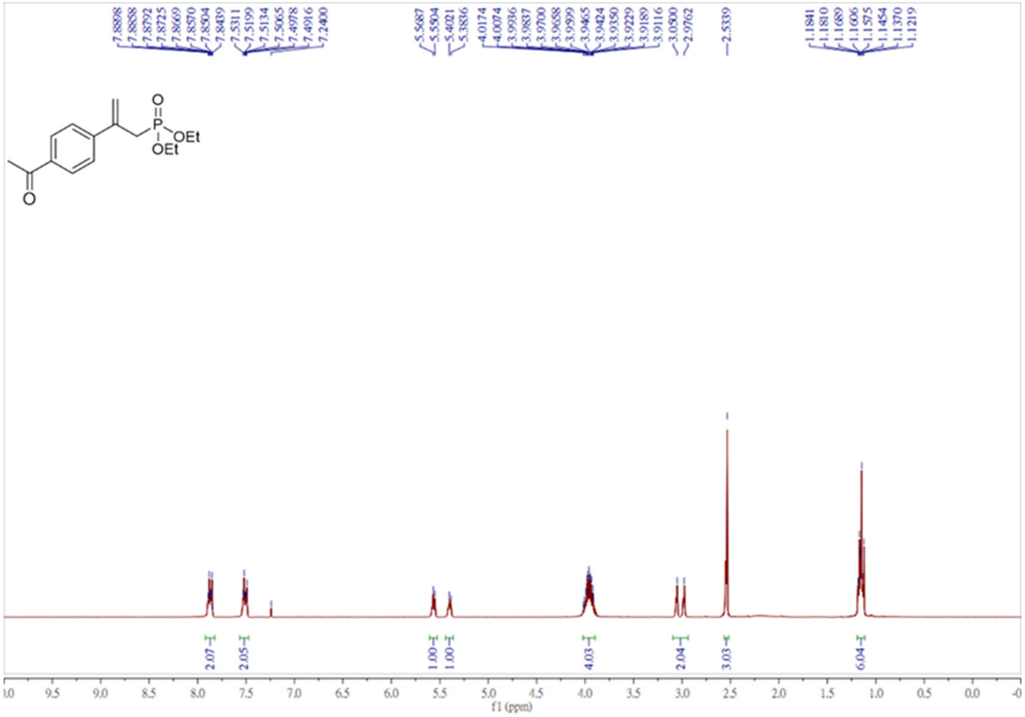

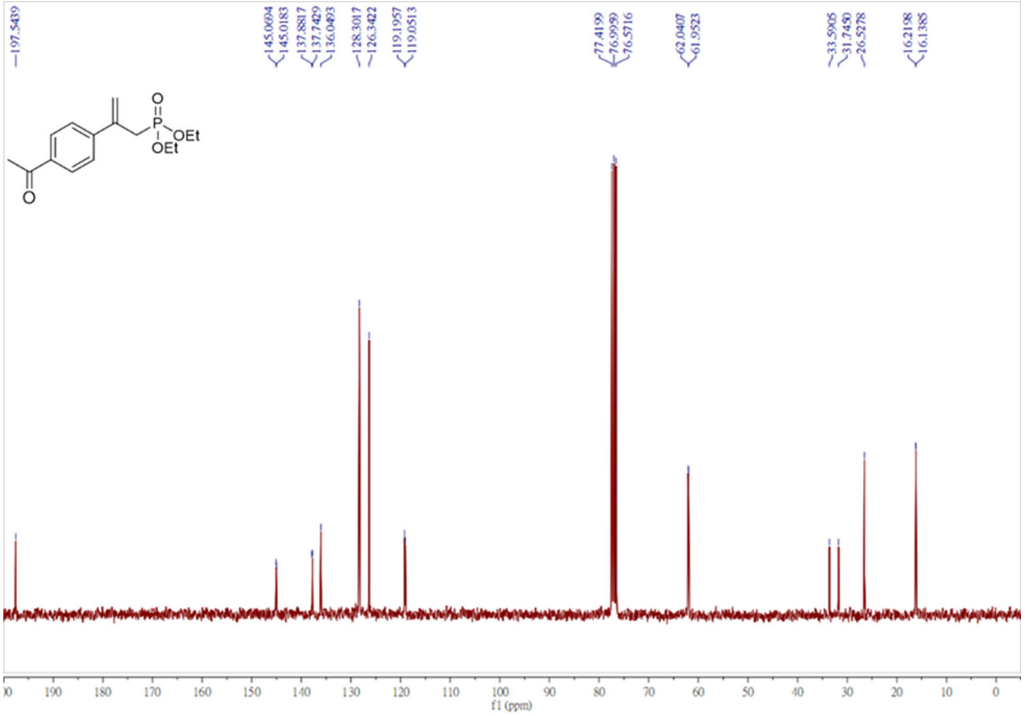

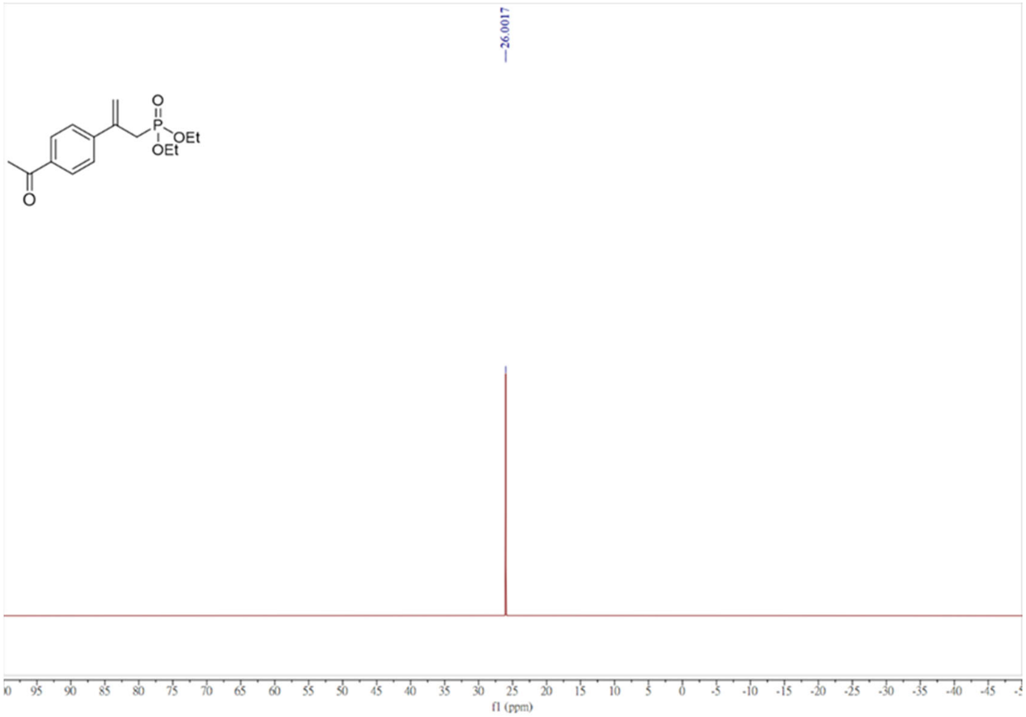

^{1}H NMR (300 MHz, $CDCl_3$), $^{13}C\{^{1}H\}$ NMR (75 MHz, $CDCl_3$), and $^{31}P\{^{1}H\}$ NMR (162 MHz, $CDCl_3$) spectra for diethyl (2-(4-cyanophenyl)allyl)phosphonate **(3ah)**

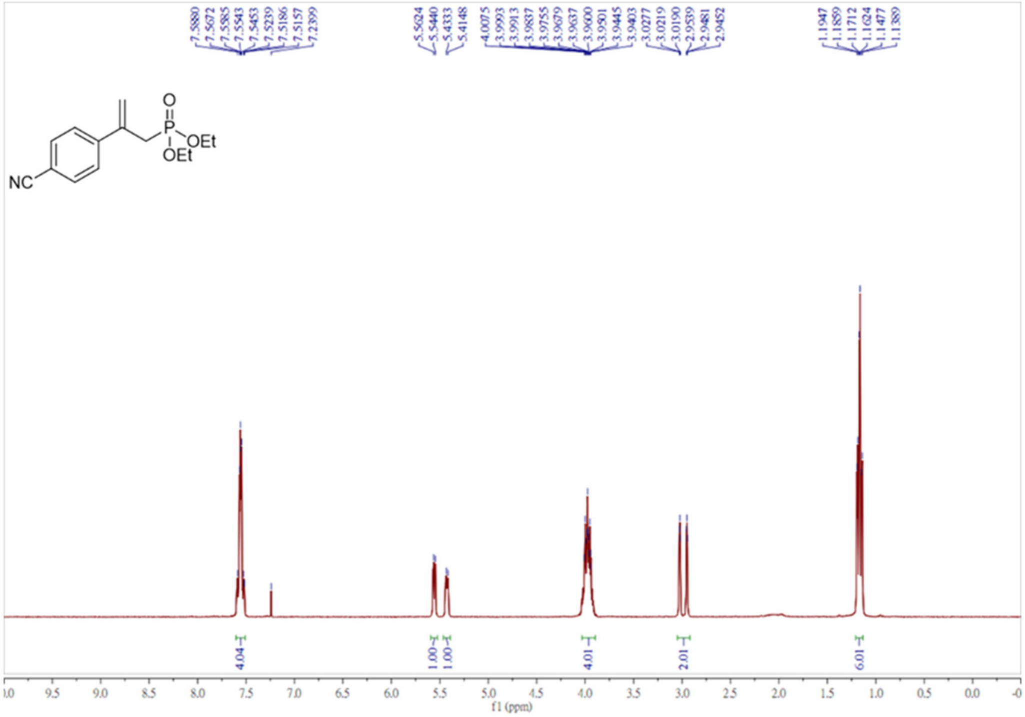

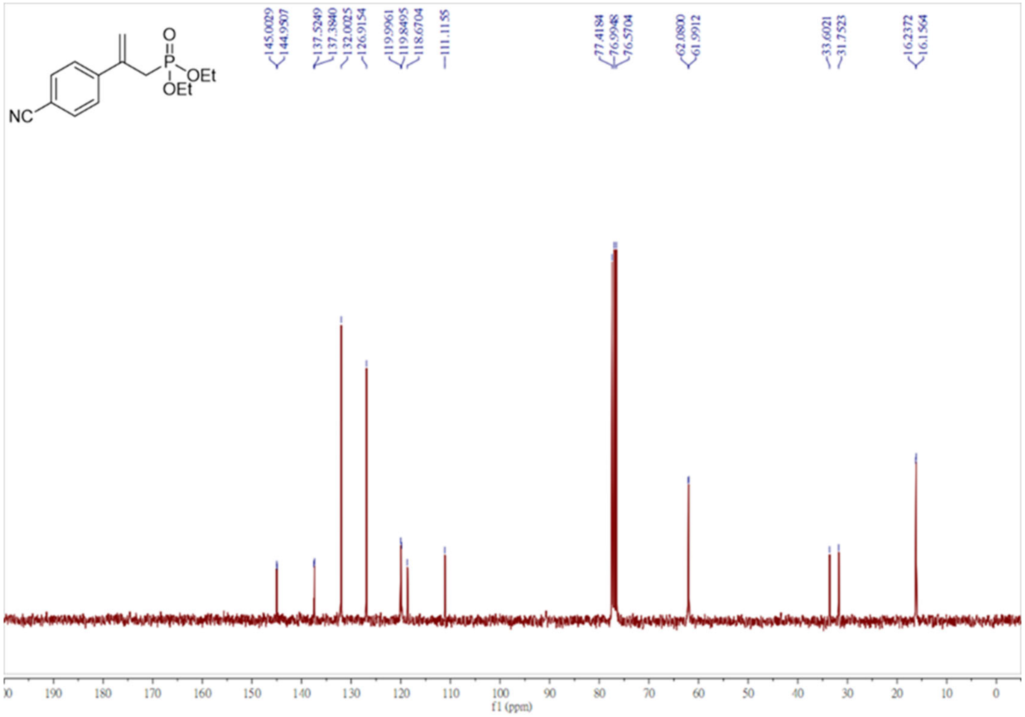

145.0029
144.9507
137.5249
137.3840
132.0025
126.9154
119.9961
119.8495
118.6704
111.1155
77.4184
76.9948
76.5704
62.0800
61.9912
33.6021
31.7523
16.2372
16.1564
NC
OEt
OEt
f1 (ppm)

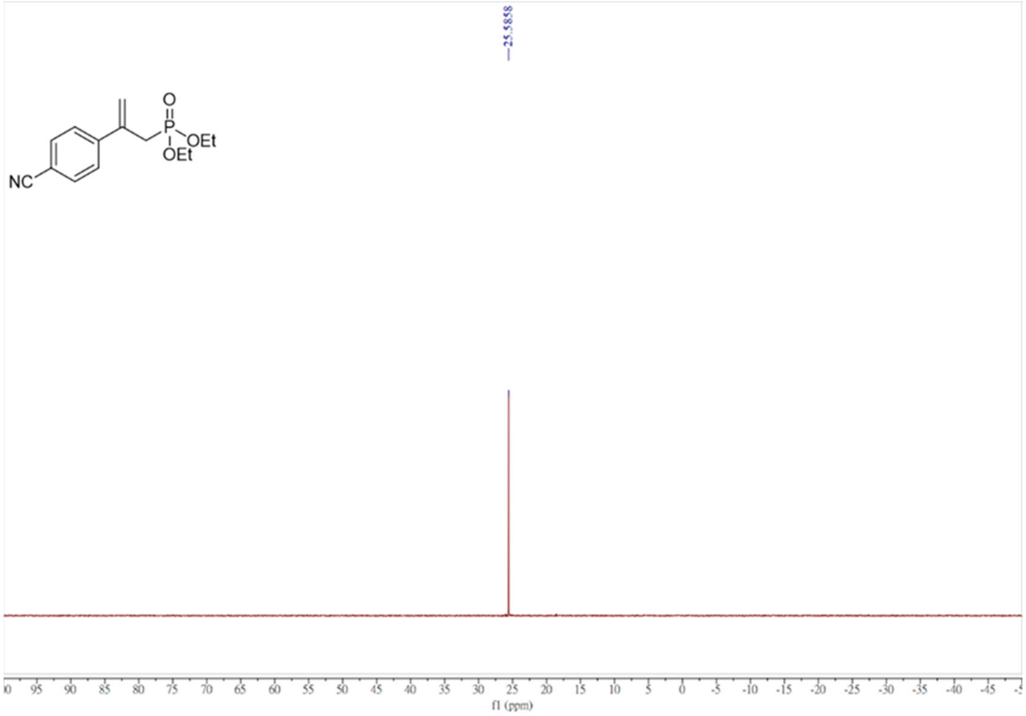

25.5858
NC
OEt
OEt
f1 (ppm)

^{1}H NMR (300 MHz, $CDCl_3$), $^{13}C\{^{1}H\}$ NMR (75 MHz, $CDCl_3$), and $^{31}P\{^{1}H\}$ NMR (162 MHz, $CDCl_3$) spectra for diethyl (2-(4-nitrophenyl)allyl)phosphonate **(3ai)**

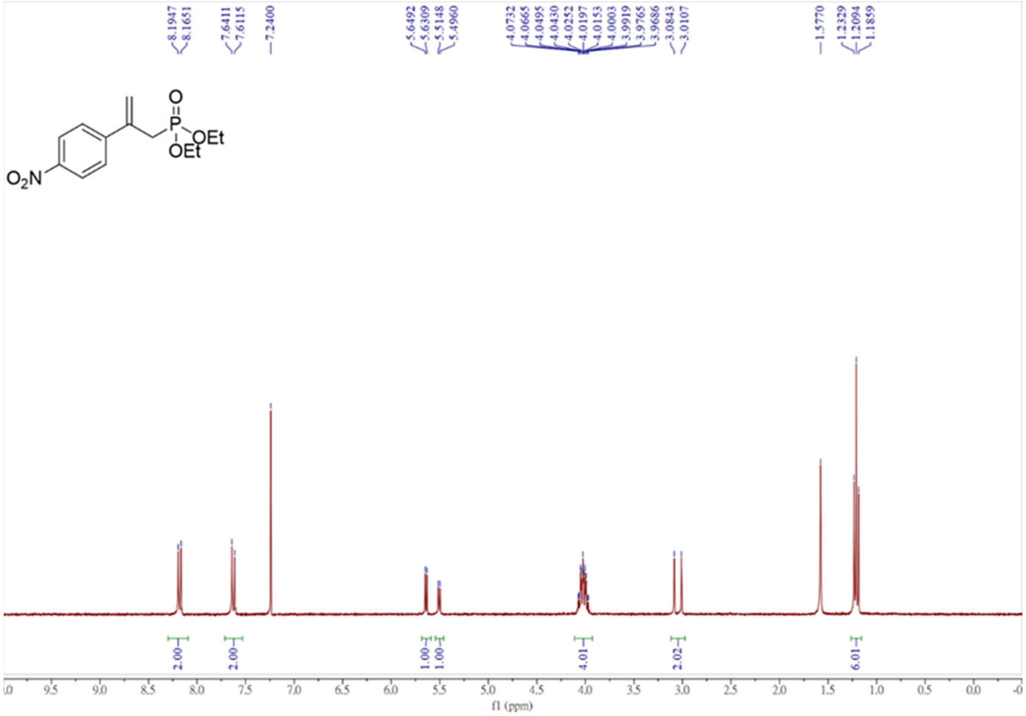

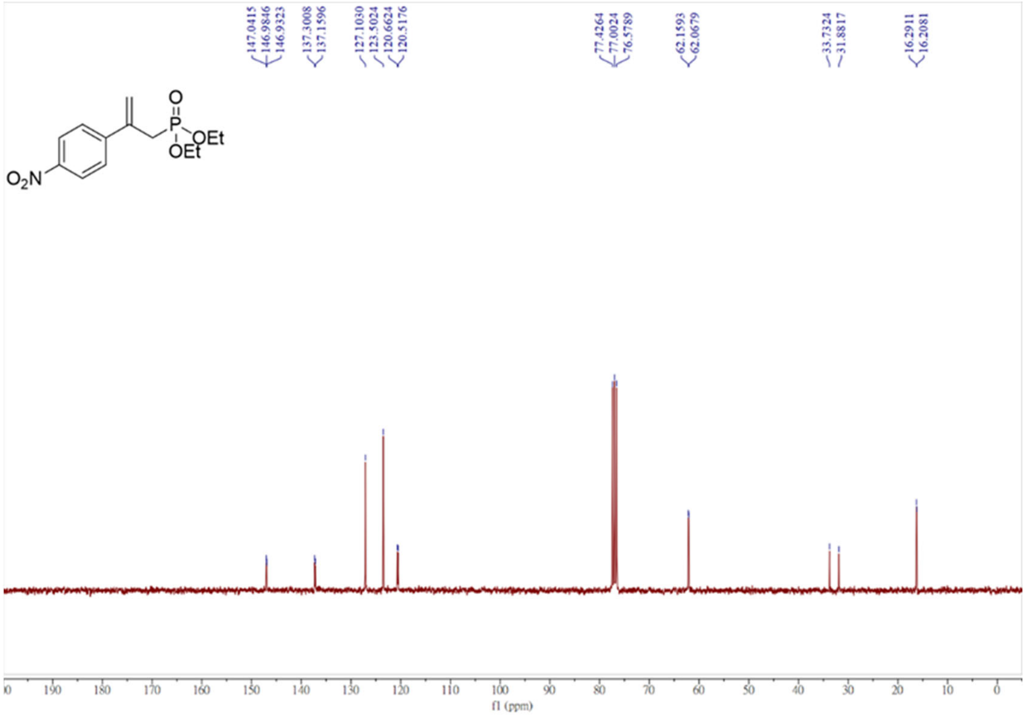

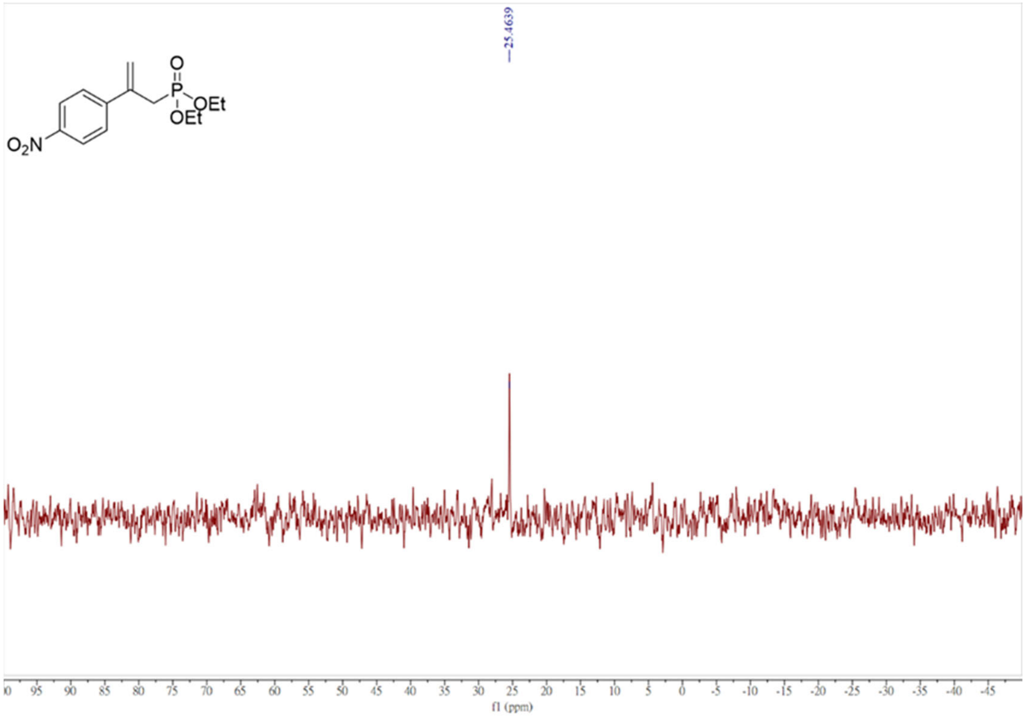

^{1}H NMR (300 MHz, $CDCl_3$), ^{13}C{^{1}H} NMR (75 MHz, $CDCl_3$), and ^{31}P{^{1}H} NMR (162 MHz, $CDCl_3$) spectra for *tert*-butyl 4-(3-(diethoxyphosphoryl)prop-1-en-2-yl)benzoate **(3aj)**

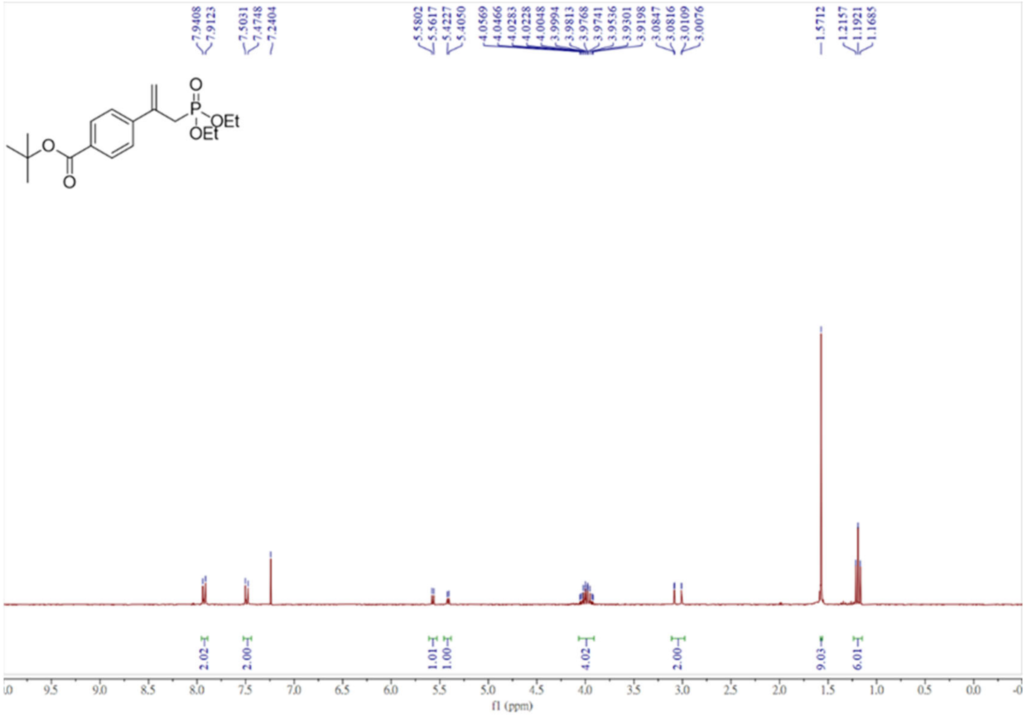

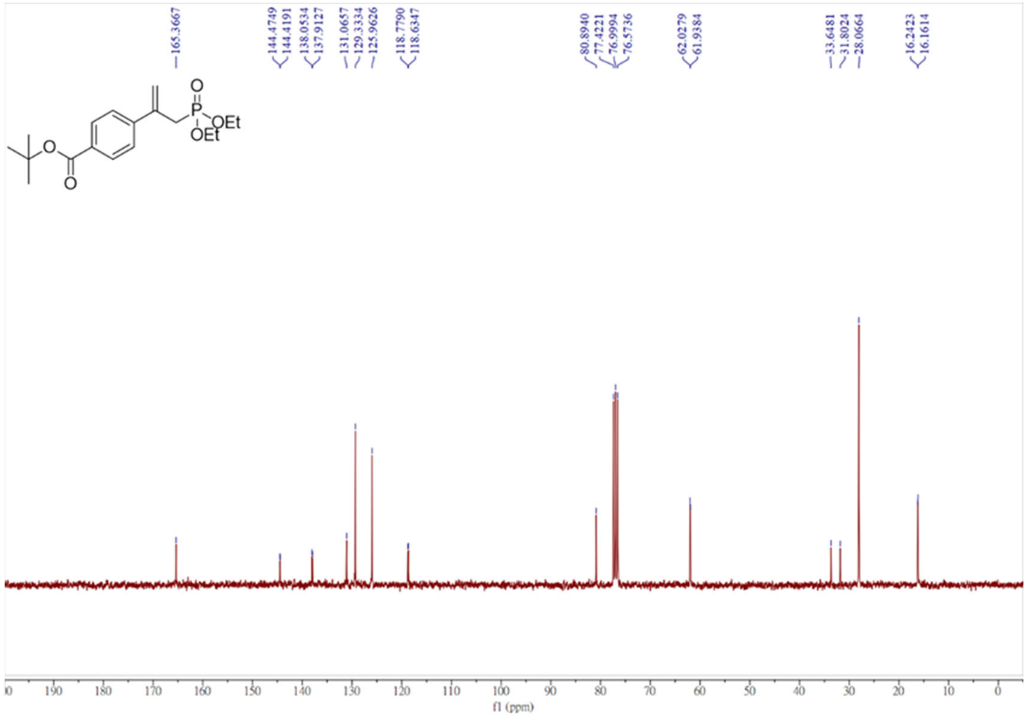
165.3667
144.4749
144.4191
138.0534
137.9127
131.0657
129.3334
125.9626
118.7790
118.6347
80.8940
77.4221
76.9994
76.5736
62.0279
61.9384
33.6481
31.8024
28.0664
16.2423
16.1614
O
P
OEt
OEt
O
O
f1 (ppm)

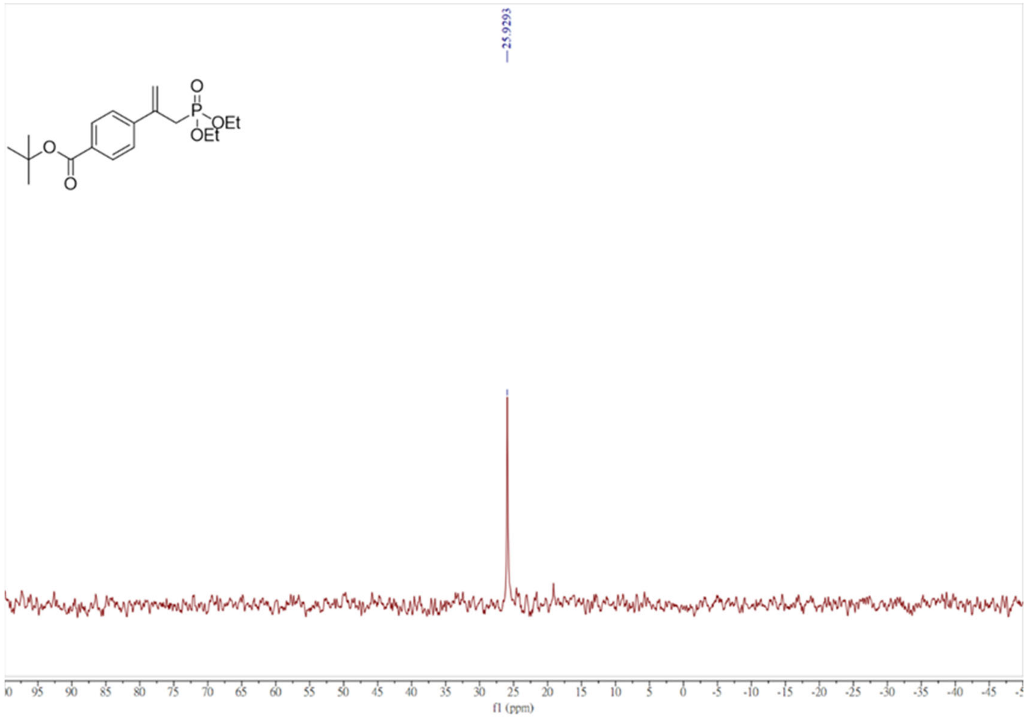
25.9293
O
P
OEt
OEt
O
O
f1 (ppm)

^1H NMR (300 MHz, $CDCl_3$), $^{13}C\{^1H\}$ NMR (75 MHz, $CDCl_3$), and $^{31}P\{^1H\}$ NMR (162 MHz, $CDCl_3$) spectra for diethyl (2-(3-(dimethylamino)phenyl)allyl)phosphonate **(3ak)**

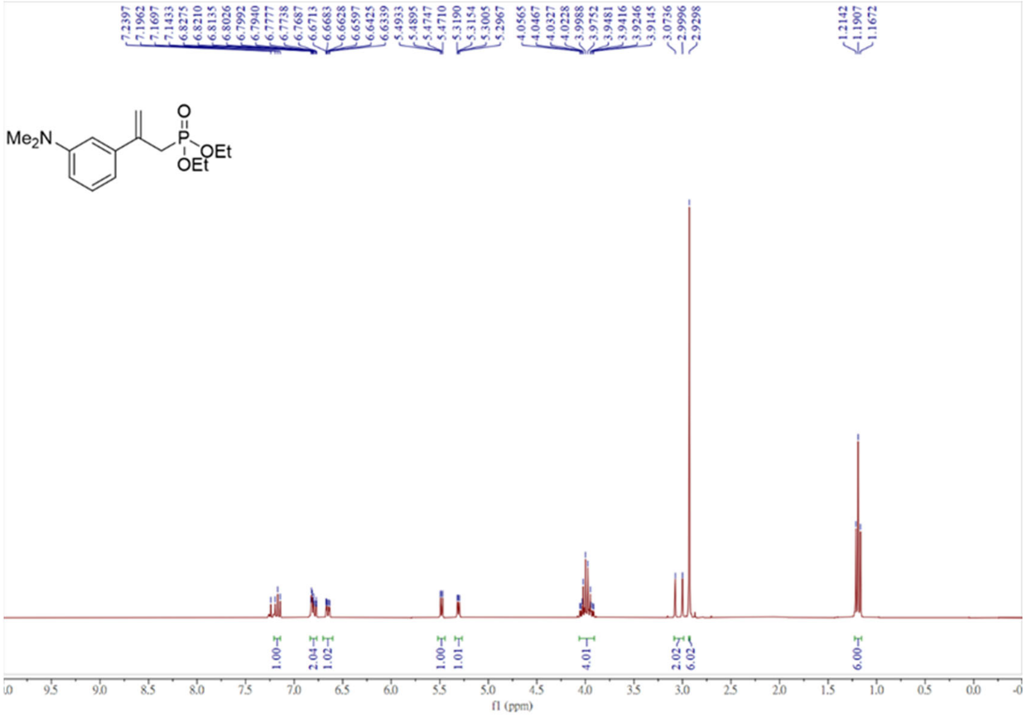

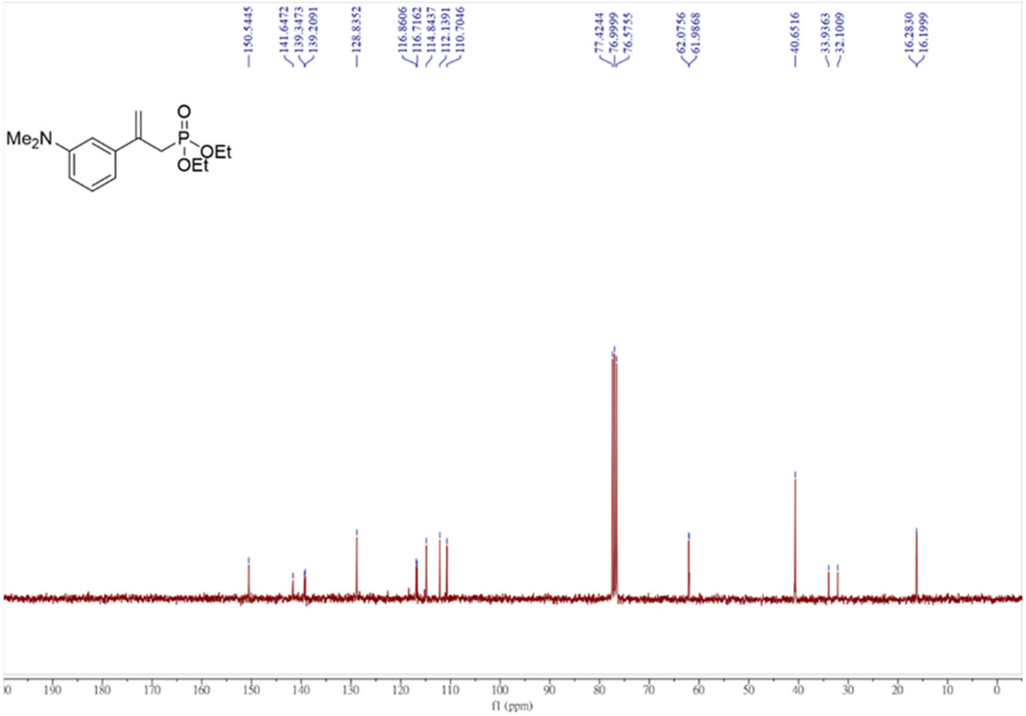

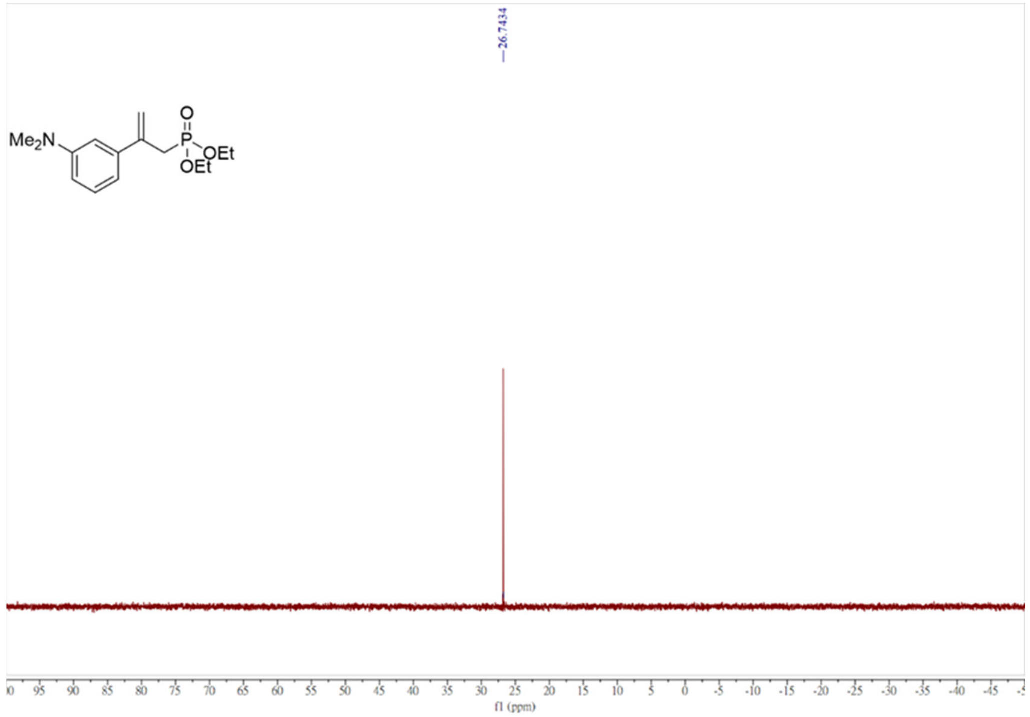

^{1}H NMR (300 MHz, $CDCl_3$), $^{13}C\{^1H\}$ NMR (75 MHz, $CDCl_3$), and $^{31}P\{^1H\}$ NMR (162 MHz, $CDCl_3$) spectra for diethyl (2-(3-methoxyphenyl)allyl)phosphonate **(3al)**

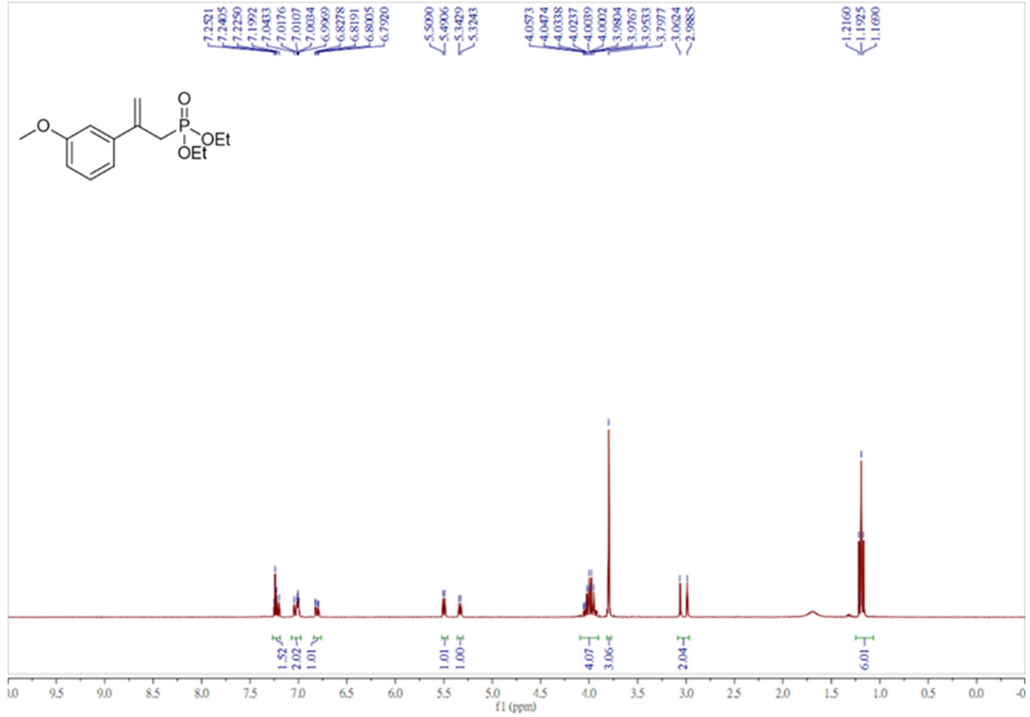

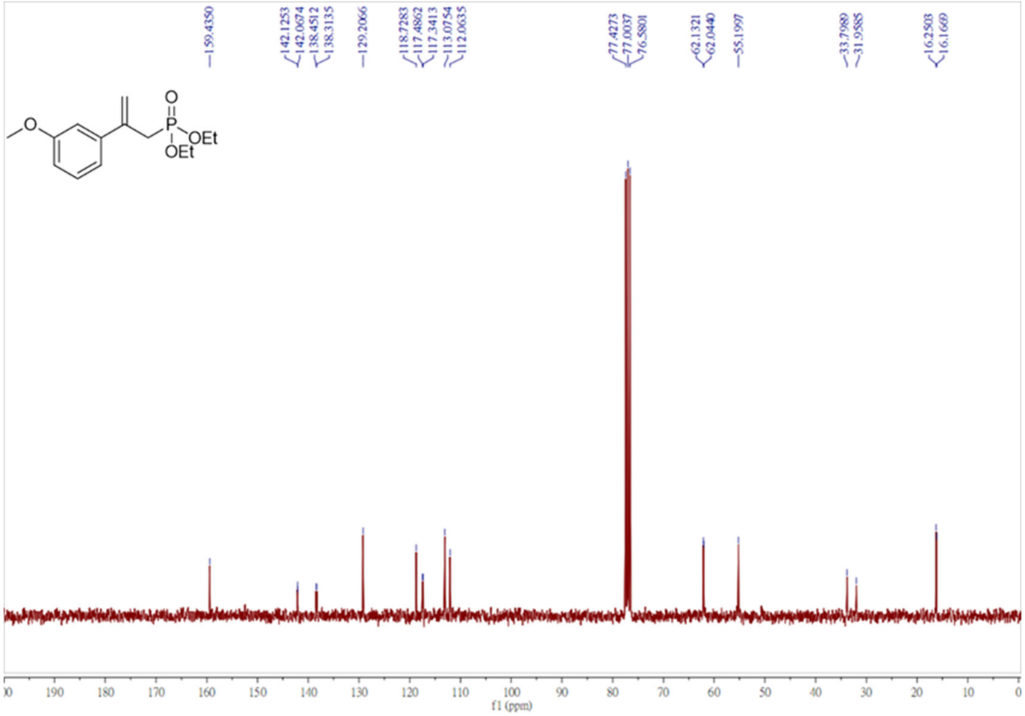
159.4350
142.1253
142.0674
138.4512
138.3135
129.2066
118.7283
117.4862
117.3413
113.0754
112.0635
77.4273
77.0037
76.5801
62.1321
62.0440
55.1997
33.7989
31.9585
16.2503
16.1669
OEt
OEt
f1 (ppm)

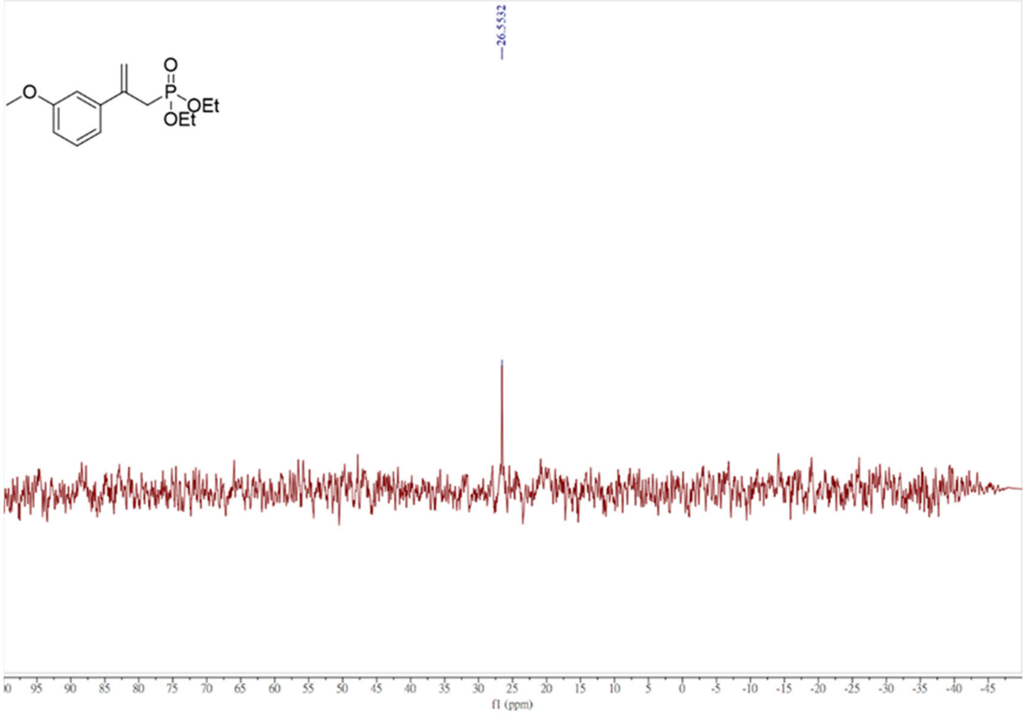
26.5532
OEt
OEt
f1 (ppm)

^{1}H NMR (300 MHz, $CDCl_3$), $^{13}C\{^{1}H\}$ NMR (75 MHz, $CDCl_3$), and $^{31}P\{^{1}H\}$ NMR (162 MHz, $CDCl_3$) spectra for diethyl (2-(3,5-dimethylphenyl)allyl)phosphonate **(3am)**

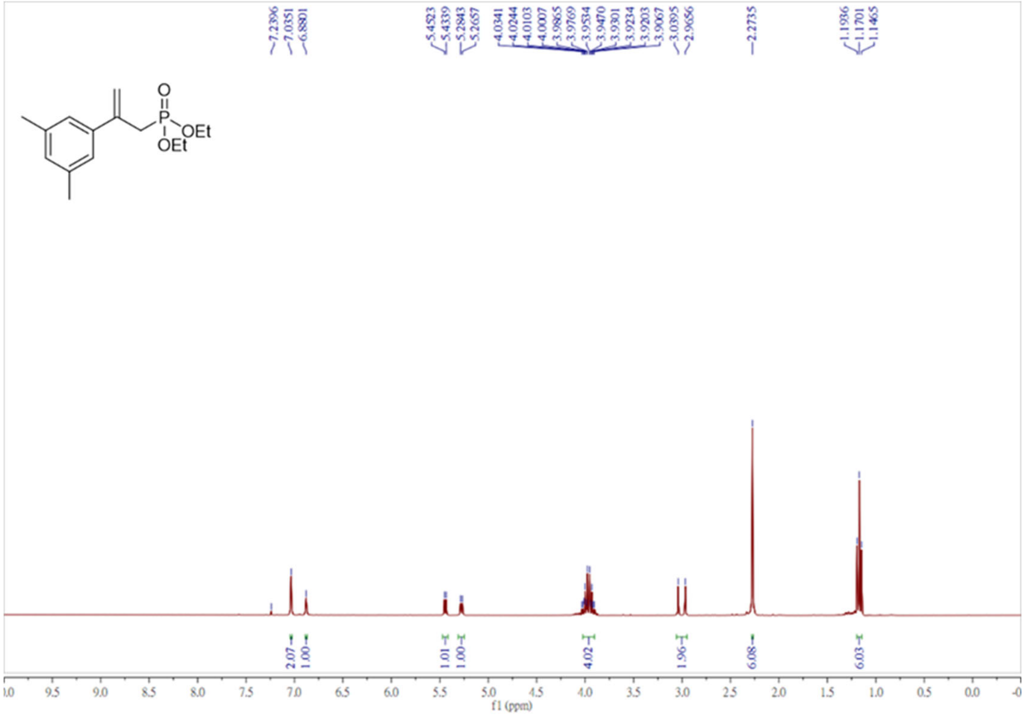

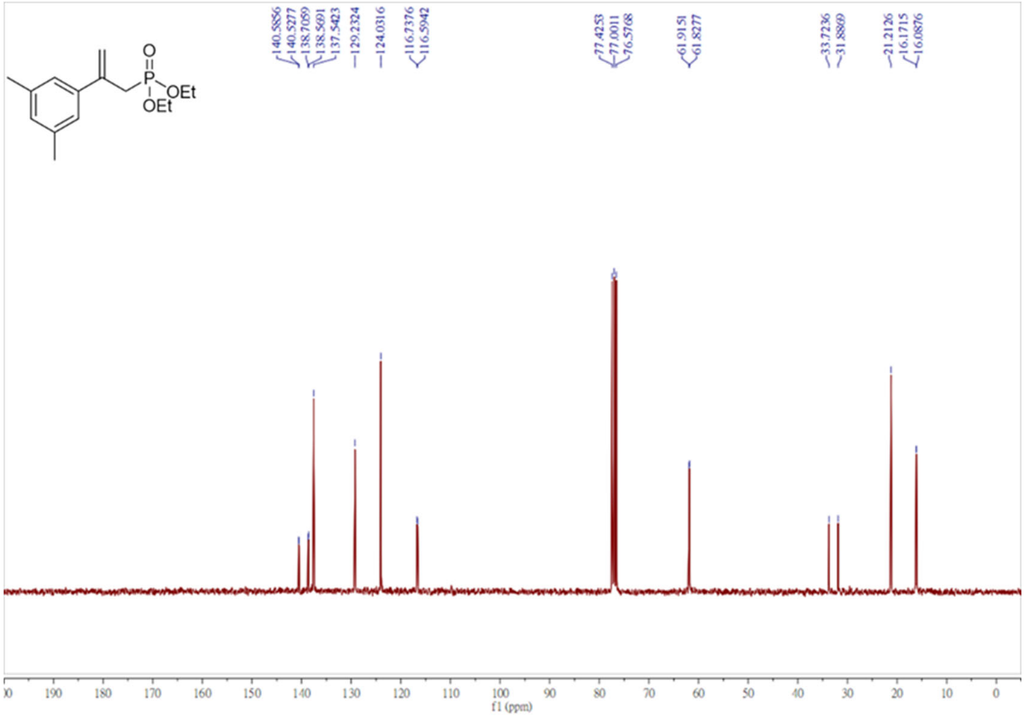

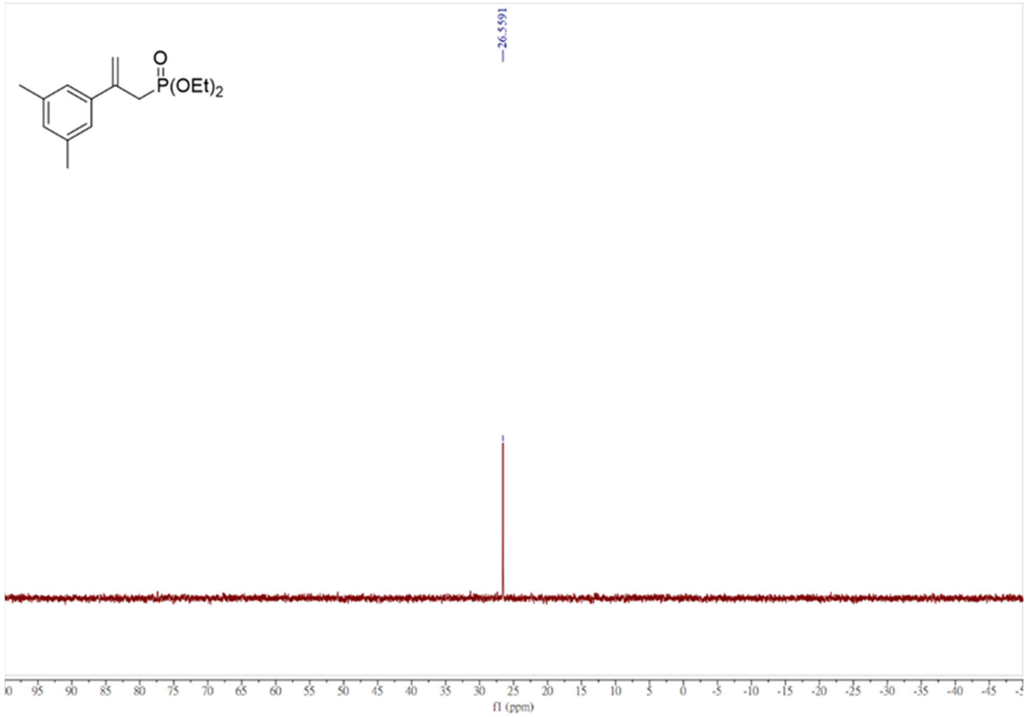

^1H NMR (300 MHz, $CDCl_3$), $^{13}C\{^1H\}$ NMR (75 MHz, $CDCl_3$), and $^{31}P\{^1H\}$ NMR (162 MHz, $CDCl_3$) spectra for diethyl (2-(3,4-dimethoxyphenyl)allyl)phosphonate **(3an)**

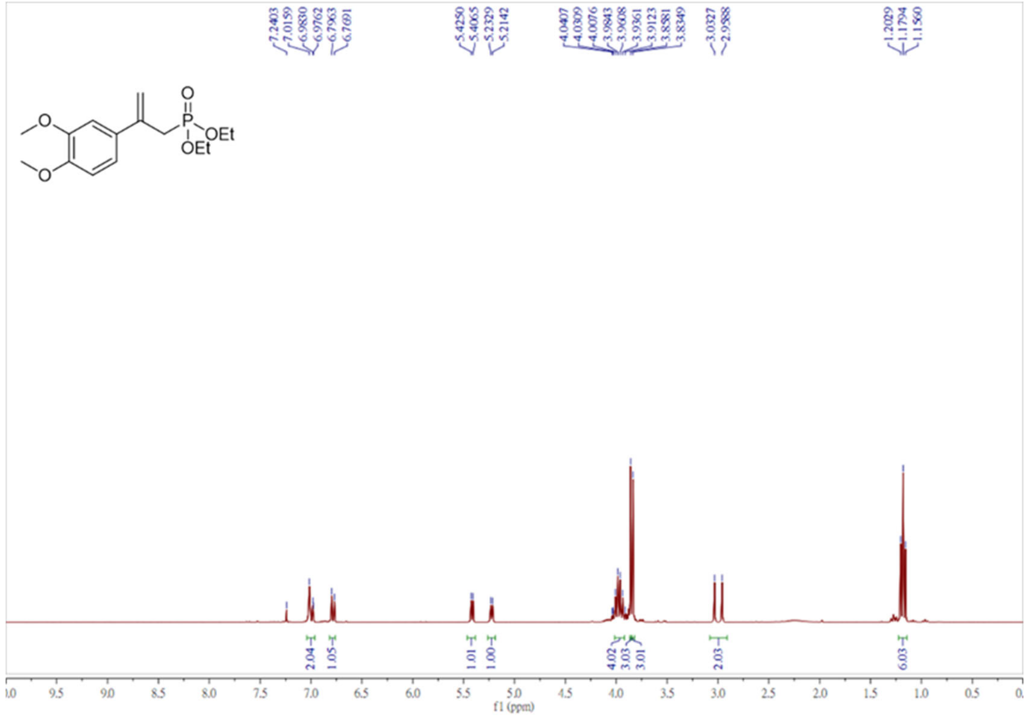

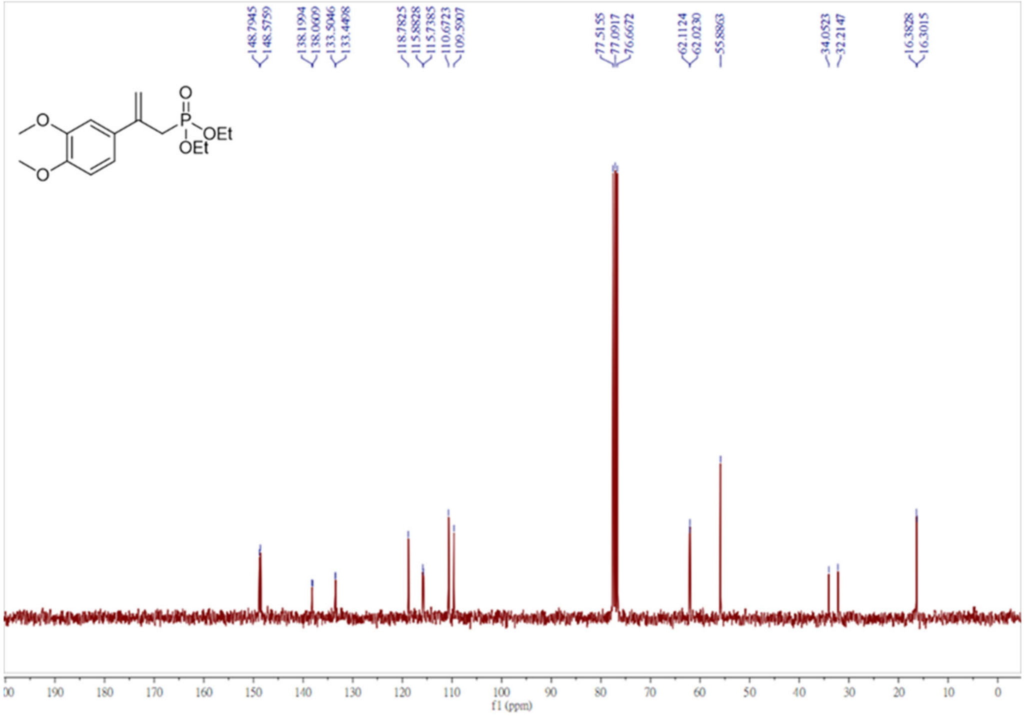
148.7945
148.5759
138.1994
138.0609
133.5046
133.4408
118.7825
115.8828
115.7385
110.6723
109.5907
77.5155
77.0917
76.6672
62.1124
62.0230
55.8863
34.0523
32.2147
16.3828
16.3015
O
P
OEt
OEt
f1 (ppm)

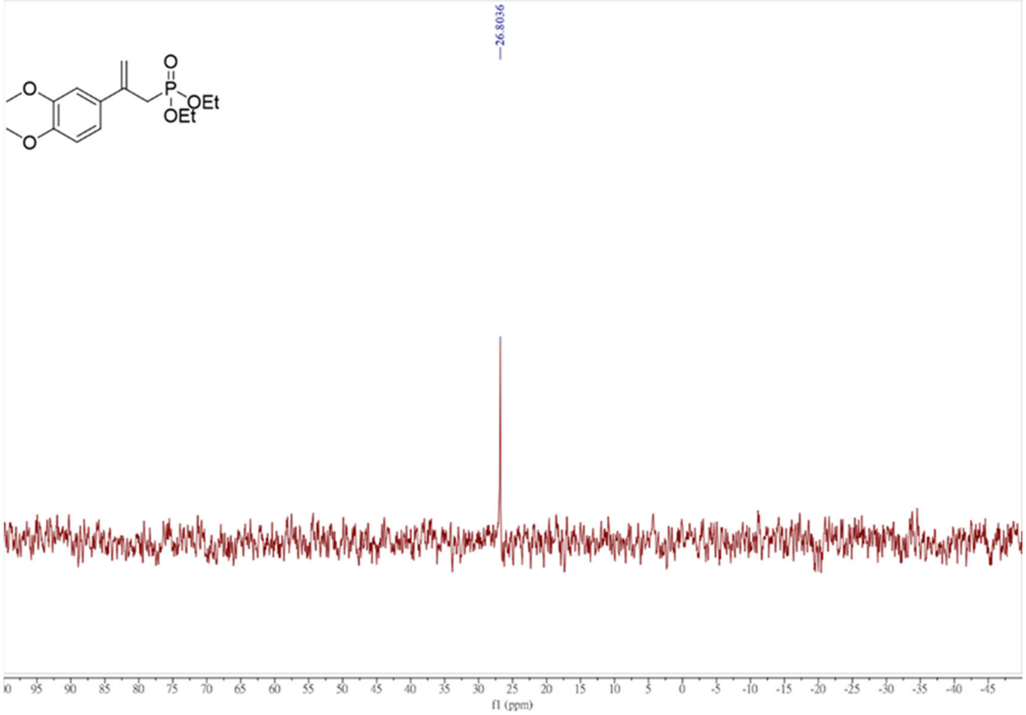
26.8036
O
P
OEt
OEt
f1 (ppm)

^{1}H NMR (400 MHz, $CDCl_3$), ^{13}C{^{1}H} NMR (100 MHz, $CDCl_3$), and ^{31}P{^{1}H} NMR (162 MHz, $CDCl_3$) spectra for diethyl (2-(naphthalen-2-yl)allyl)phosphonate **(3ao)**

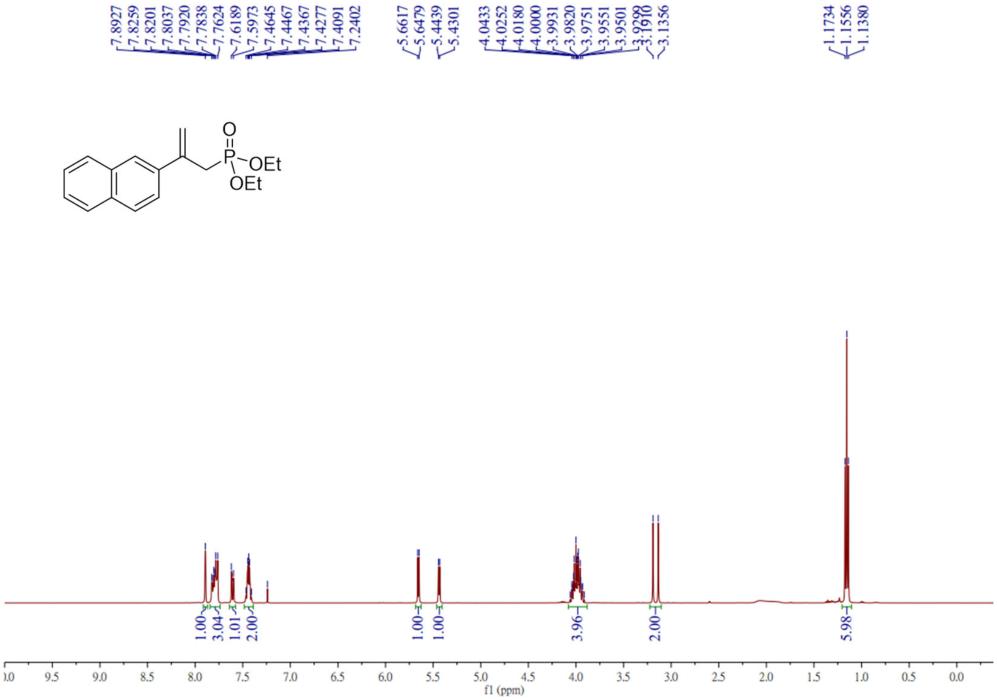

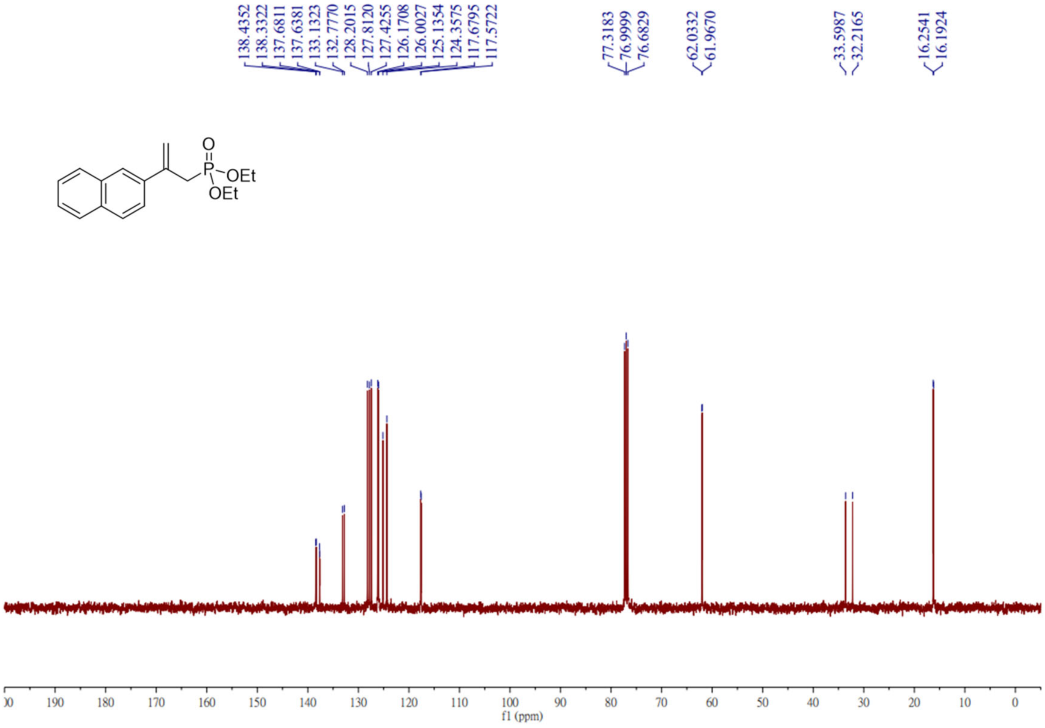

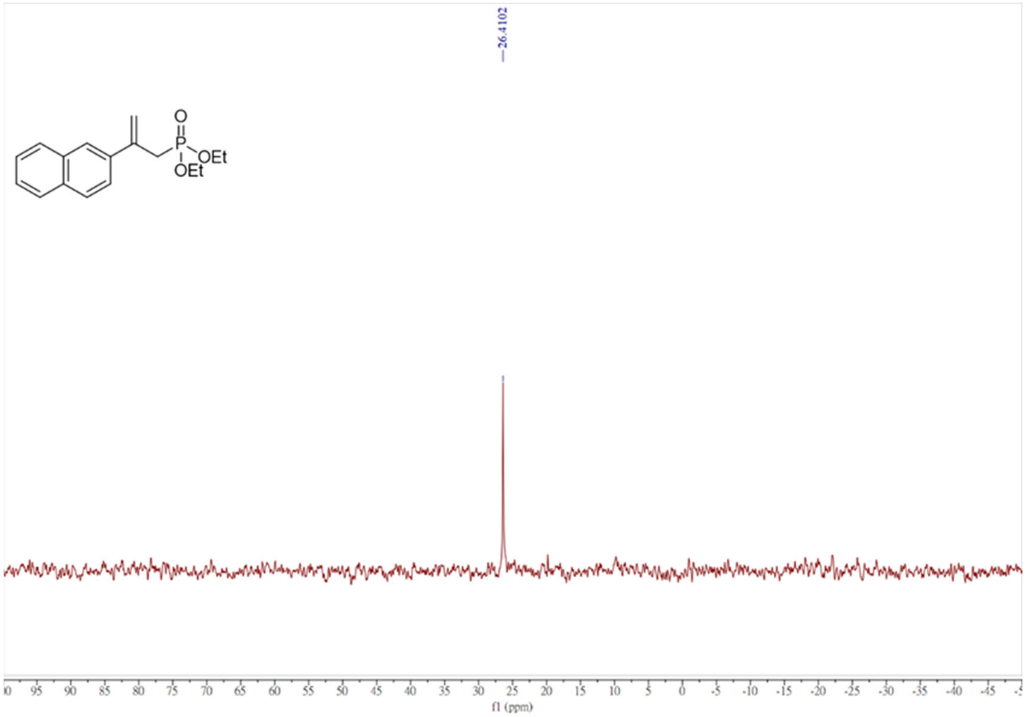

^{1}H NMR (300 MHz, CDCl3), ^{13}C{^{1}H} NMR (75 MHz, CDCl3), and ^{31}P{^{1}H} NMR (162 MHz, CDCl3) spectra for diethyl (2-(naphthalen-1-yl)allyl)phosphonate **(3ap)**

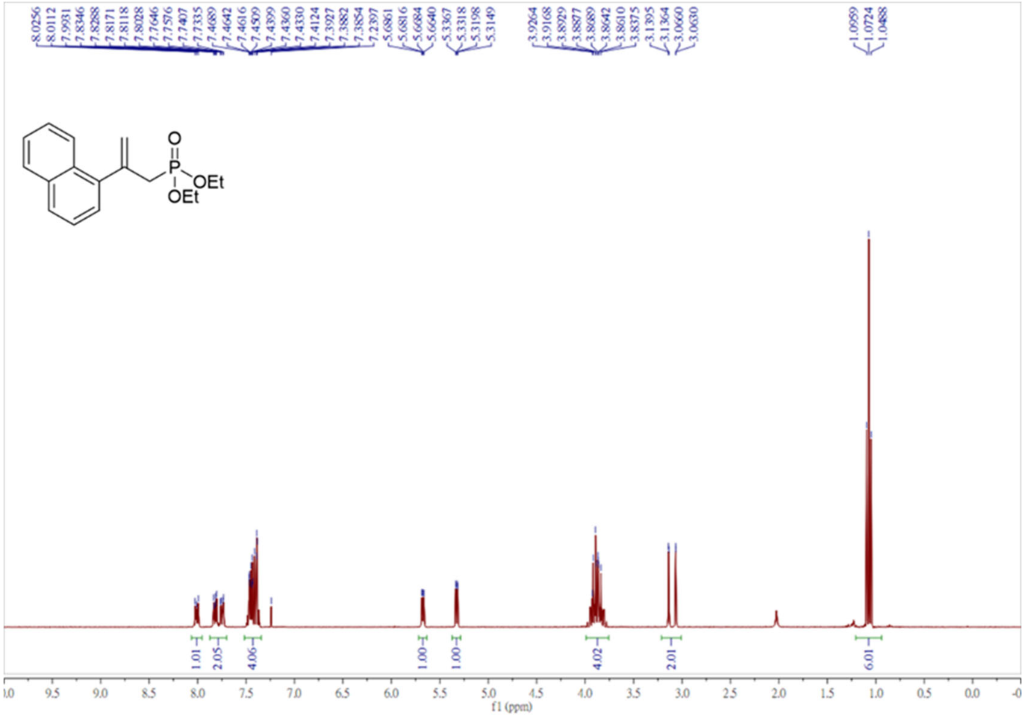

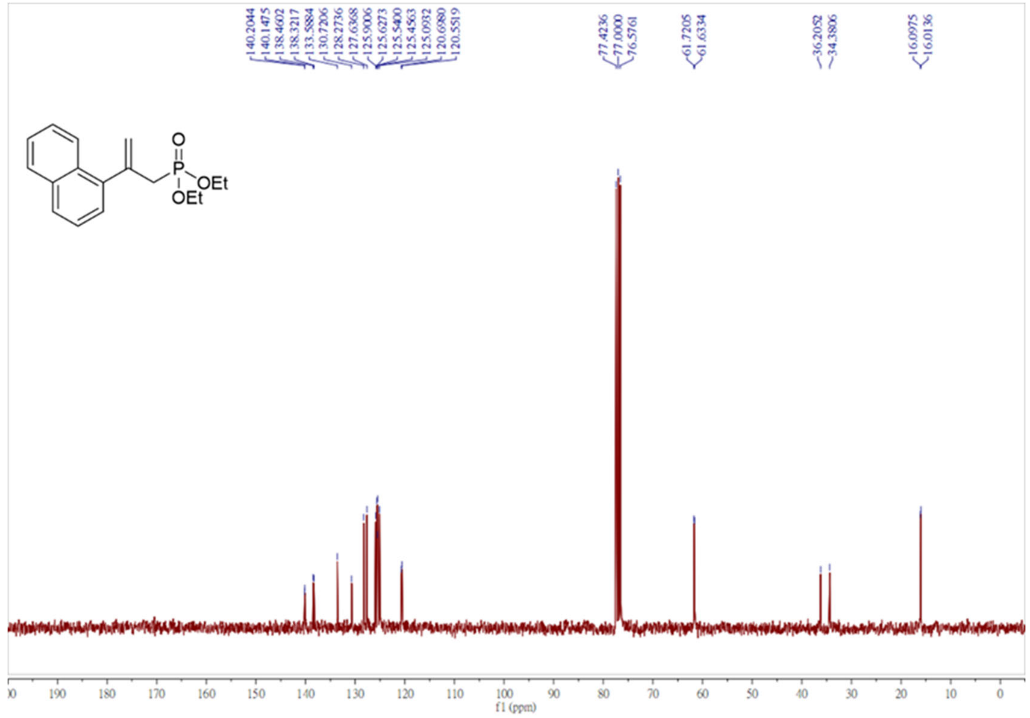
140.2044
140.1475
138.4602
138.3217
133.5884
130.7206
128.2736
127.6368
125.9006
125.6273
125.5400
125.4563
125.0932
120.6980
120.5519
77.4236
77.0000
76.5761
61.7205
61.6334
36.2052
34.3806
16.0975
16.0136
OEt
OEt
O
P
f1 (ppm)

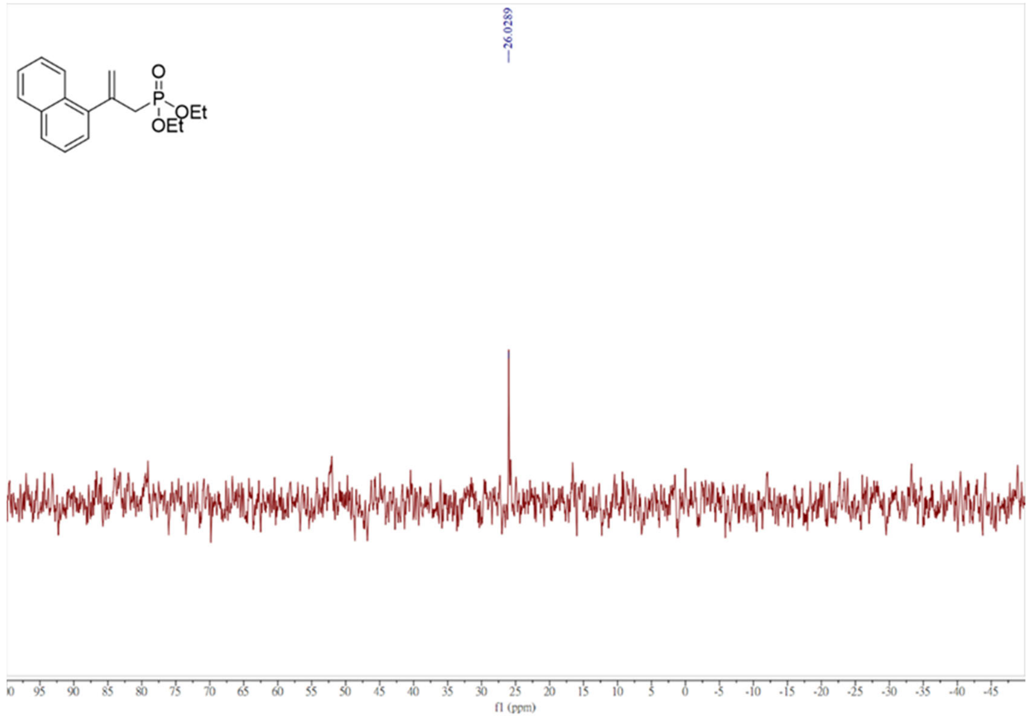
26.0289
O
P
OEt
OEt
f1 (ppm)

^{1}H NMR (300 MHz, $CDCl_3$), ^{13}C{^{1}H} NMR (75 MHz, $CDCl_3$), and ^{31}P{^{1}H} NMR (162 MHz, $CDCl_3$) spectra for diethyl (2-(2-methoxyphenyl)allyl)phosphonate **(3aq)**

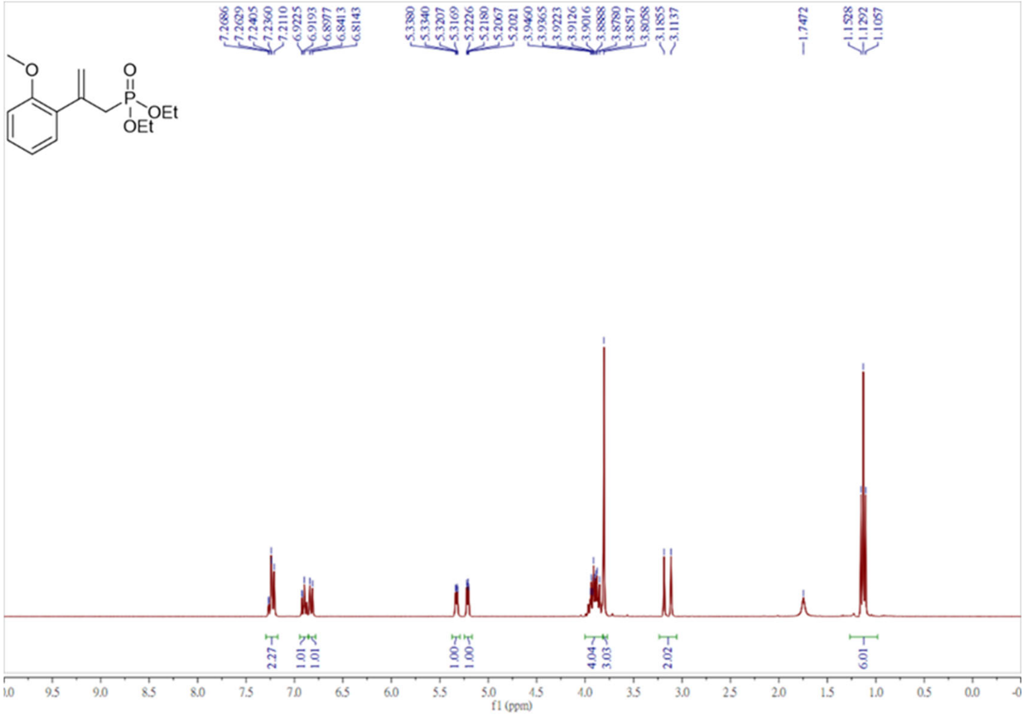

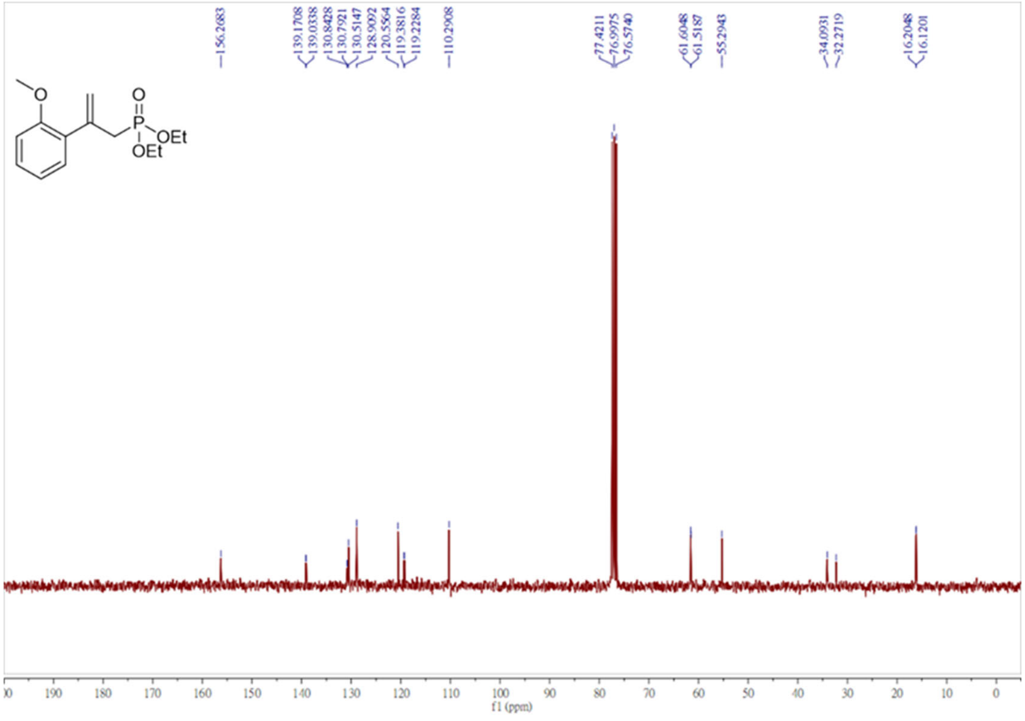

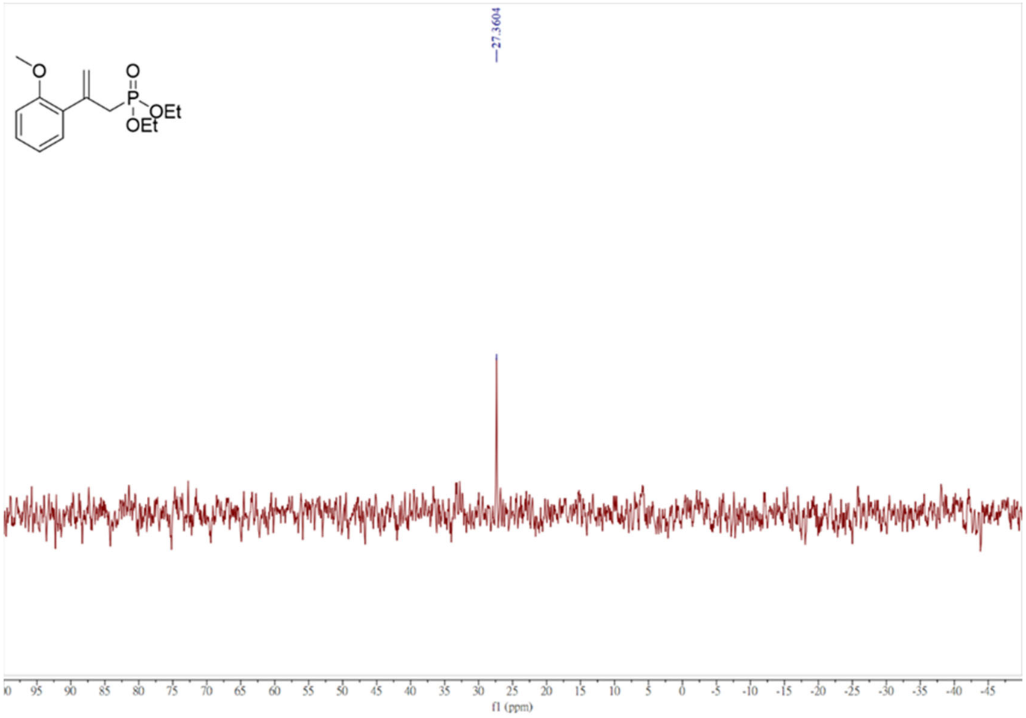

27.3604
O
OEt
OEt
f1 (ppm)

^{1}H NMR (300 MHz, $CDCl_3$) and $^{13}C\{^1H\}$ NMR (75 MHz, $CDCl_3$) spectra for ((2-bromoallyl)sulfonyl)benzene **(4a)**

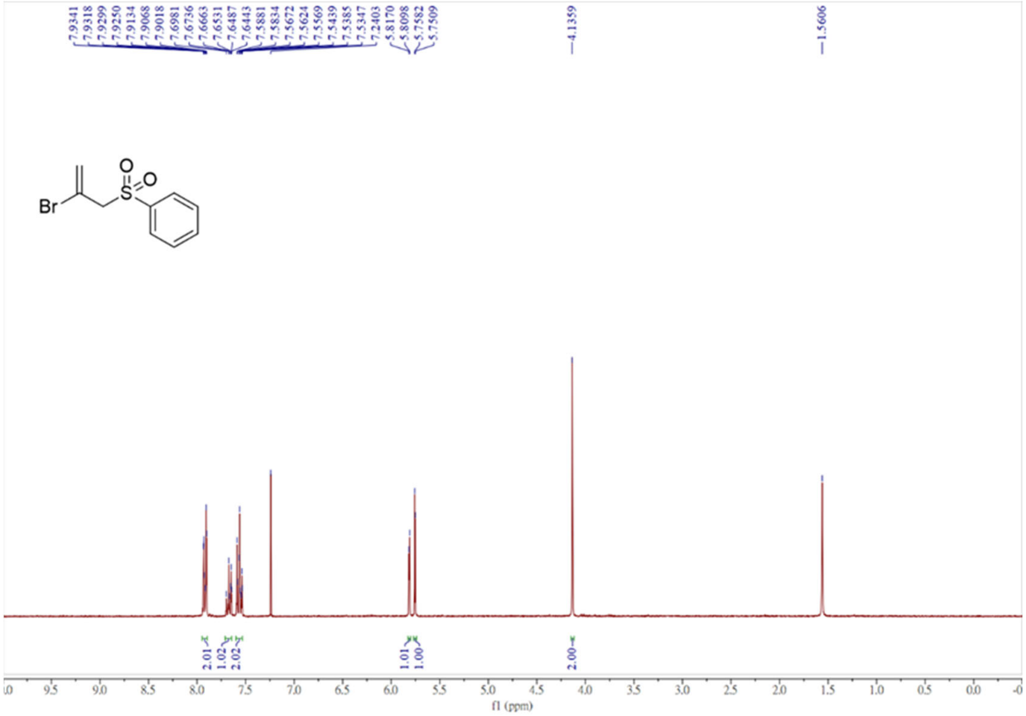

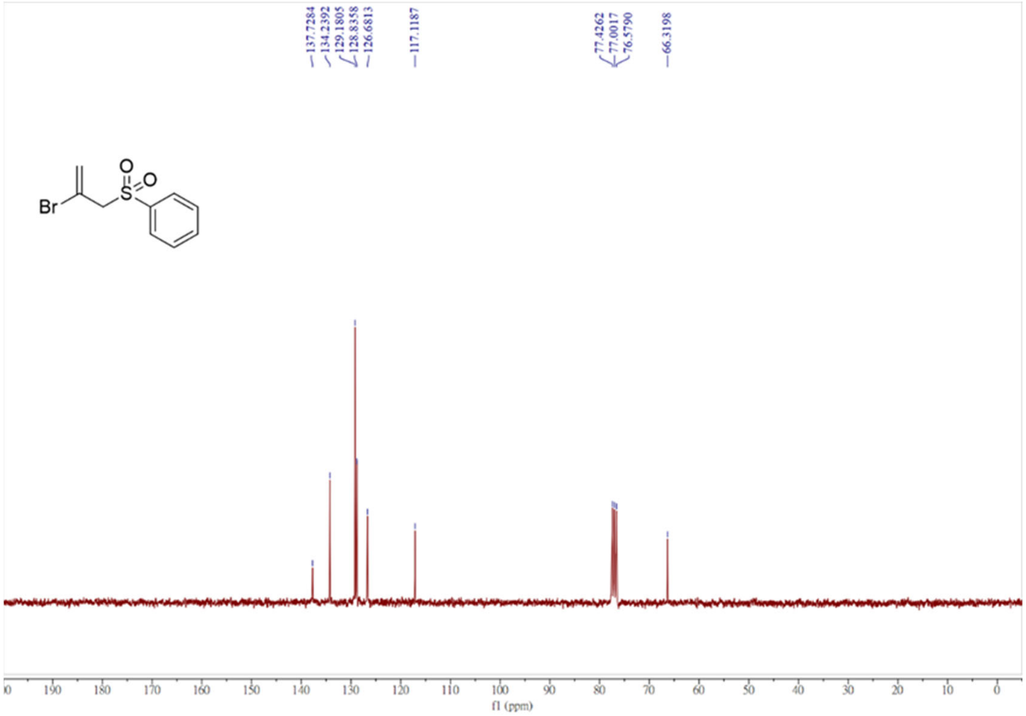

^{1}H NMR (300 MHz, $CDCl_3$) and $^{13}C\{^{1}H\}$ NMR (75 MHz, $CDCl_3$) spectra for 1-((2-bromoallyl)sulfonyl)-4-methylbenzene **(4b)**

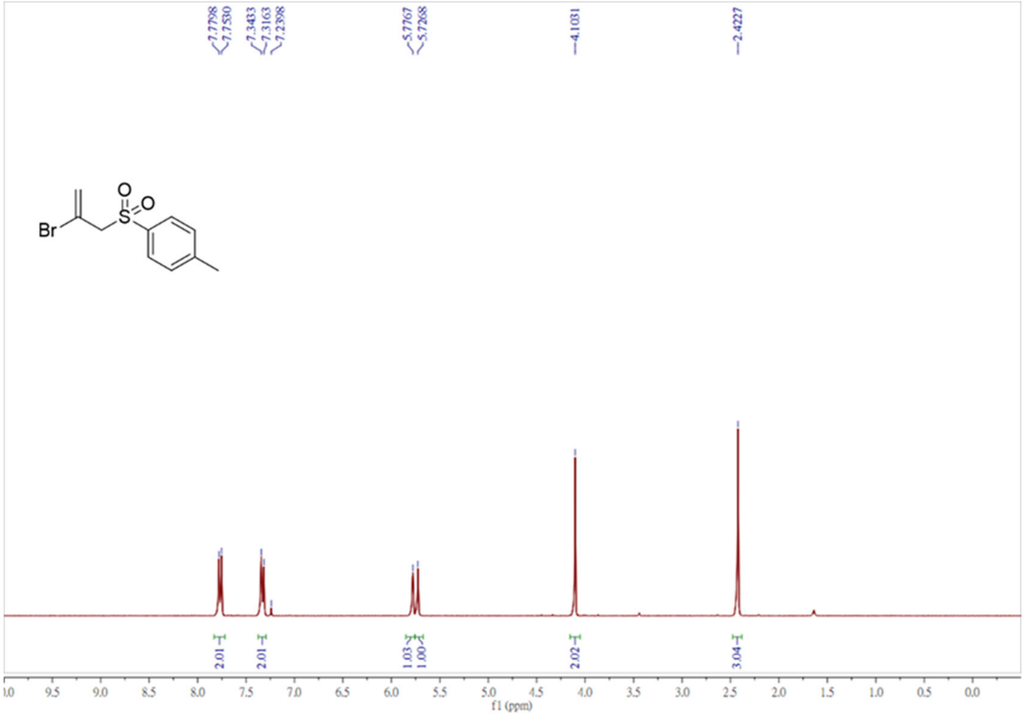

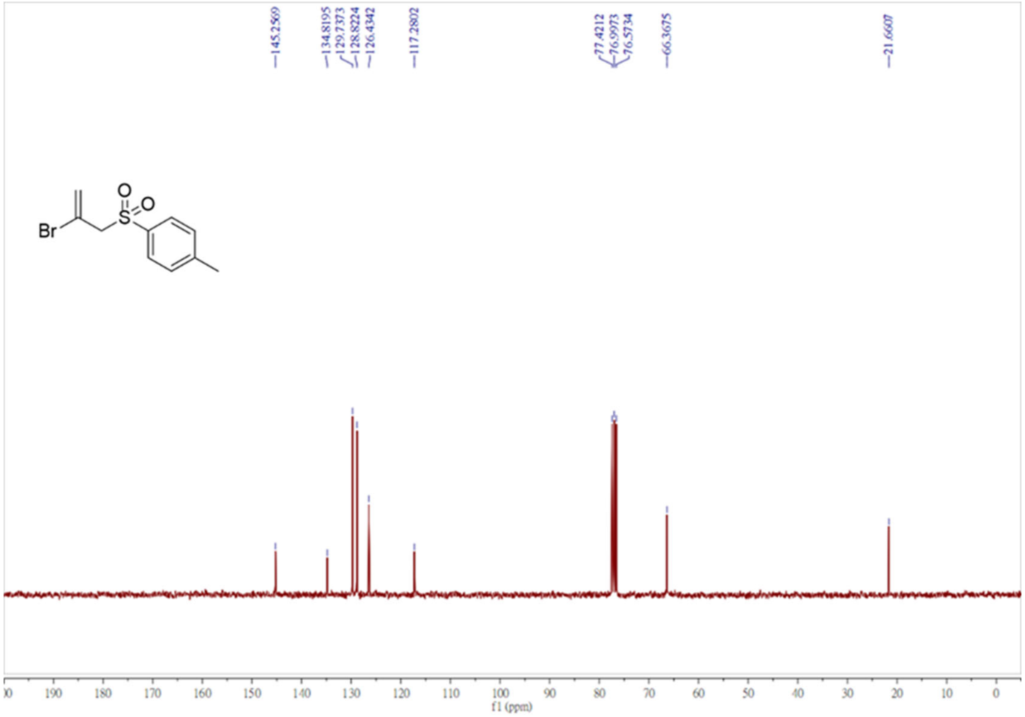

^1H NMR (300 MHz, $CDCl_3$) and $^{13}C\{^1H\}$ NMR (75 MHz, $CDCl_3$) spectra for ((2-phenylallyl)sulfonyl)benzene **(5aa)**

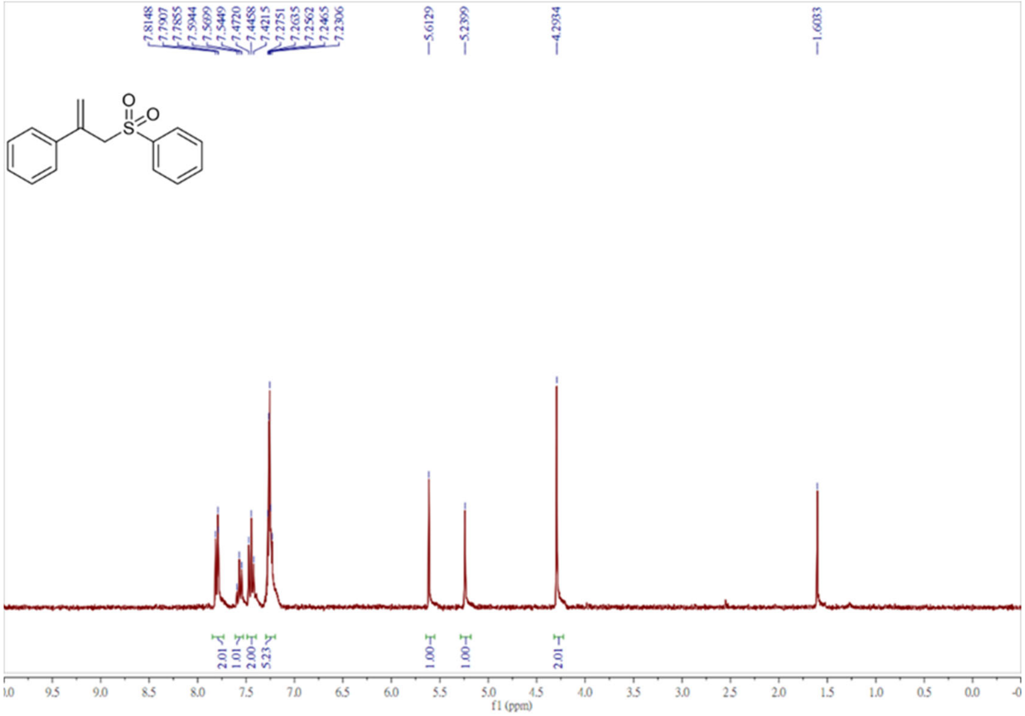

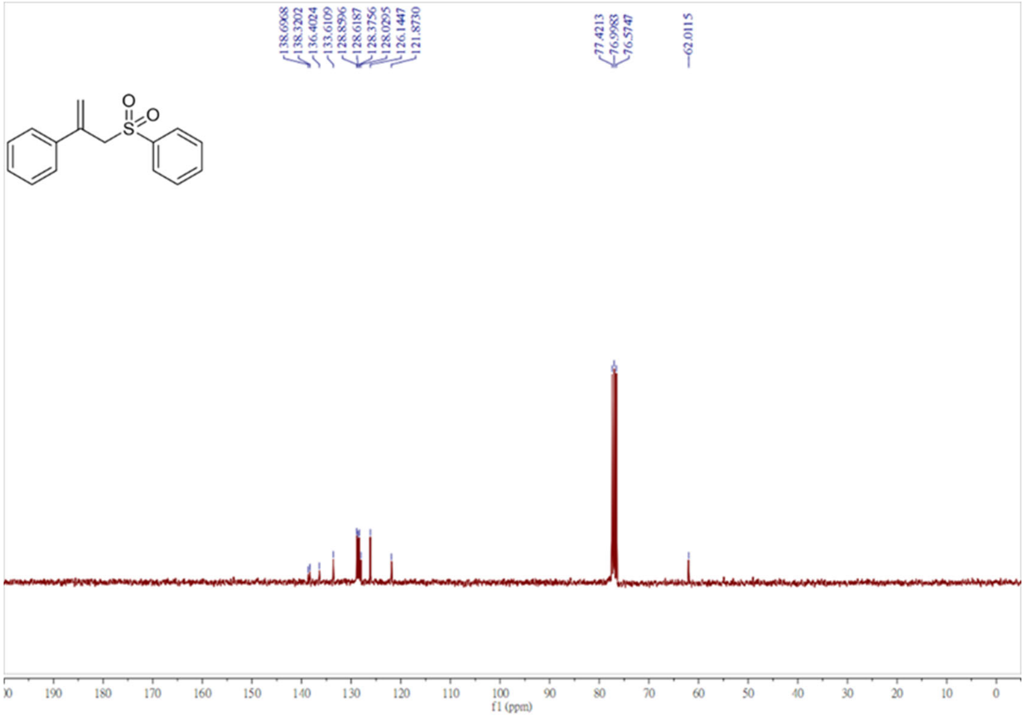

^{1}H NMR (300 MHz, $CDCl_3$) and $^{13}C\{^{1}H\}$ NMR (75 MHz, $CDCl_3$) spectra for 1-methyl-4-(3-(phenylsulfonyl)prop-1-en-2-yl)benzene **(5ab)**

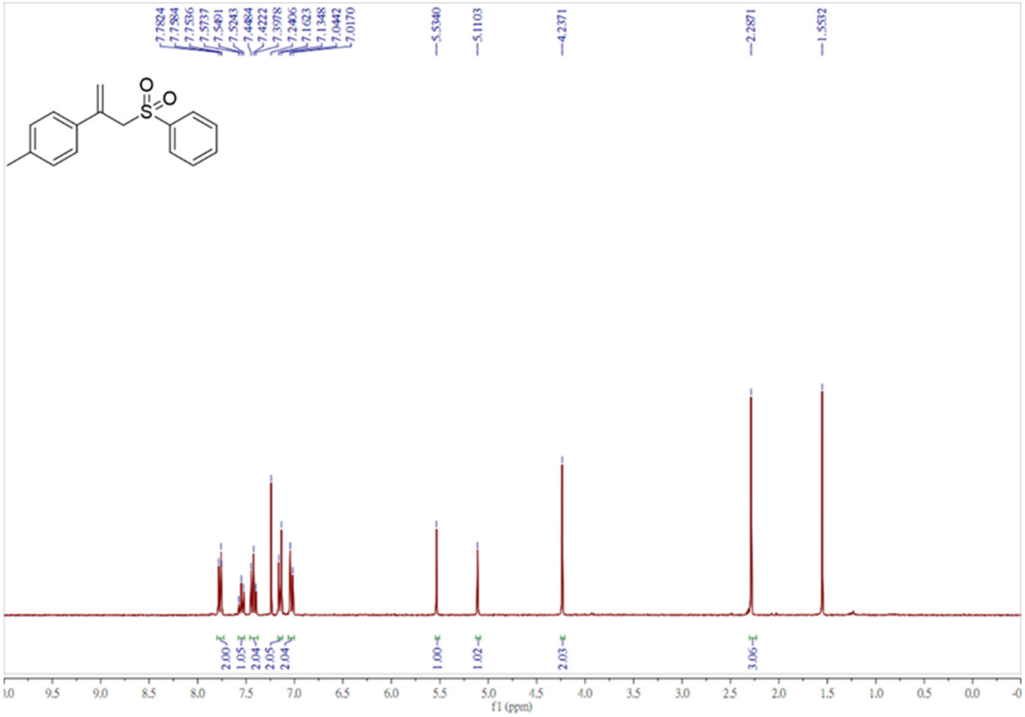

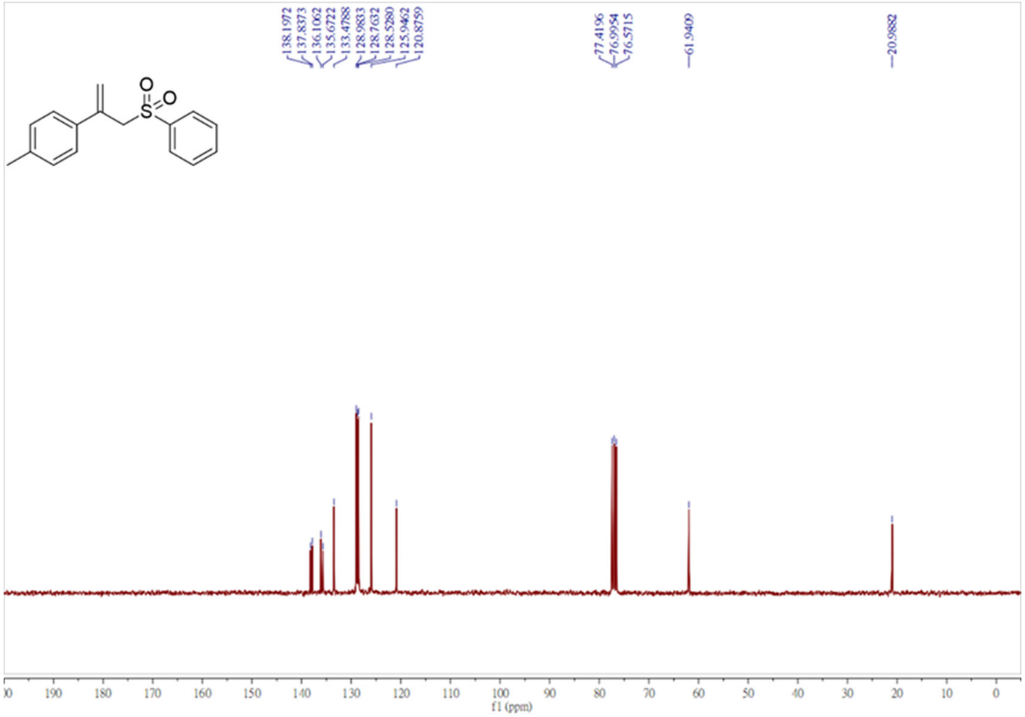

^{1}H NMR (300 MHz, $CDCl_3$) and $^{13}C\{^{1}H\}$ NMR (75 MHz, $CDCl_3$) spectra for 1-(*tert*-butyl)-4-(3-(phenylsulfonyl)prop-1-en-2-yl)benzene **(5ac)**

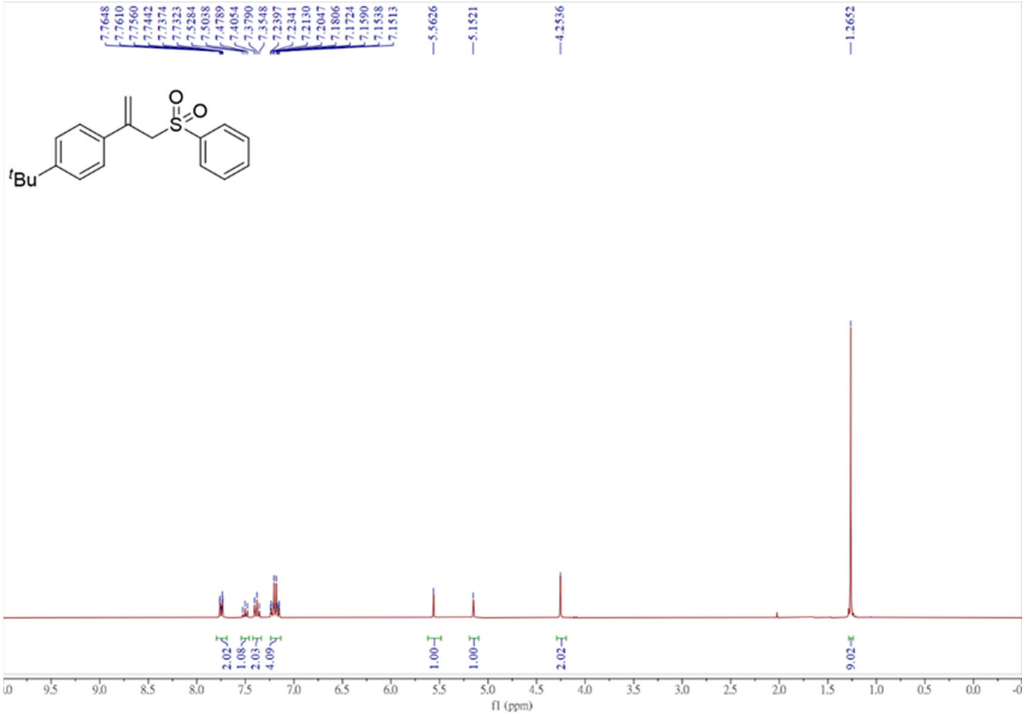

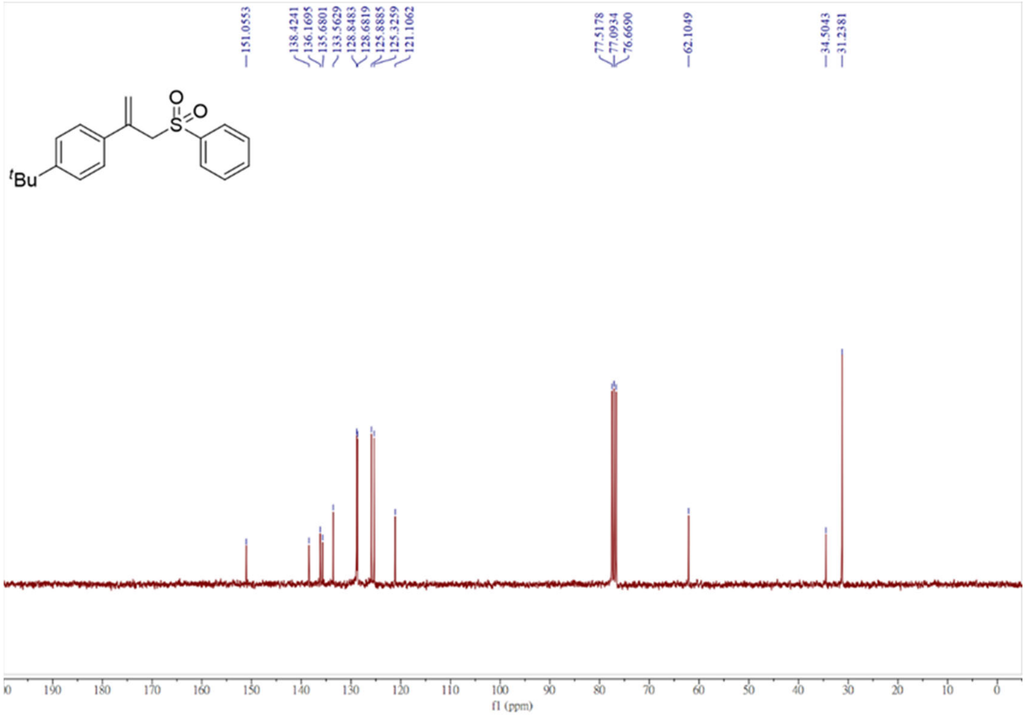

^{1}H NMR (300 MHz, $CDCl_3$) and $^{13}C\{^1H\}$ NMR (75 MHz, $CDCl_3$) spectra for 1-methoxy-4-(3-(phenylsulfonyl)prop-1-en-2-yl)benzene **(5ad)**

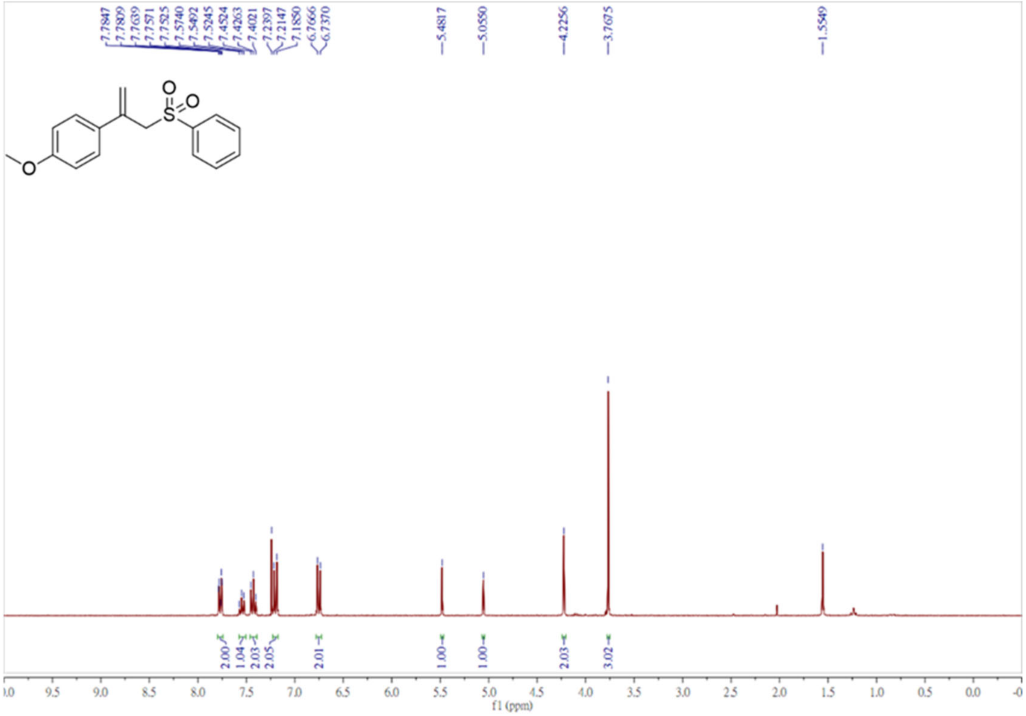

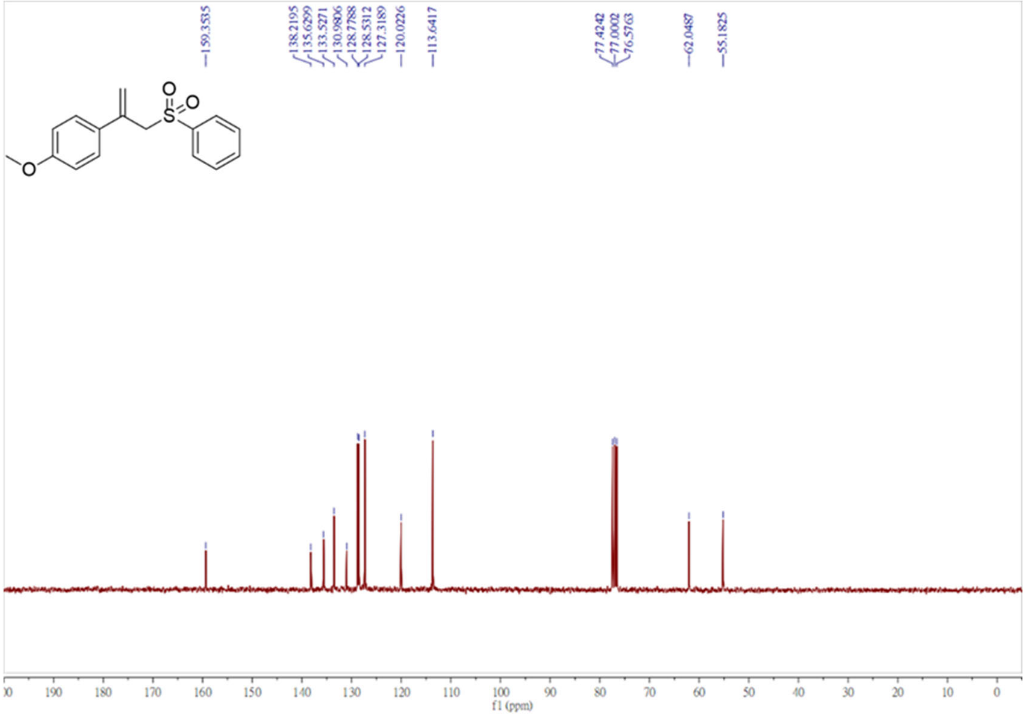

^{1}H NMR (300 MHz, $CDCl_3$) and ^{13}C{^{1}H} NMR (75 MHz, $CDCl_3$) spectra for 1-fluoro-4-(3-(phenylsulfonyl)prop-1-en-2-yl)benzene **(5ae)**

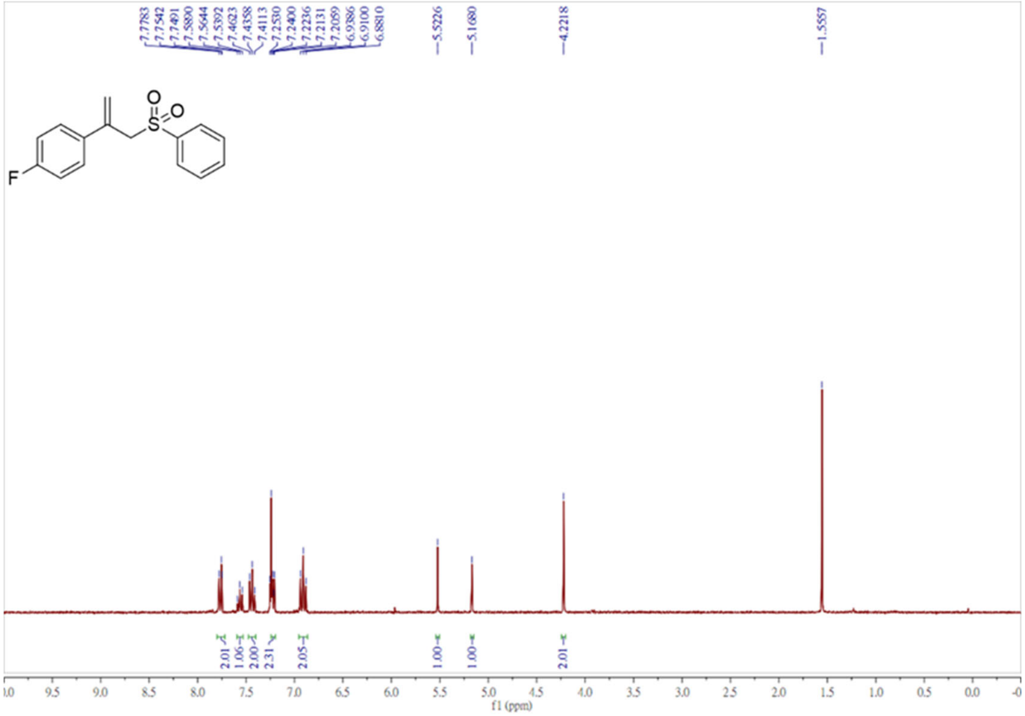

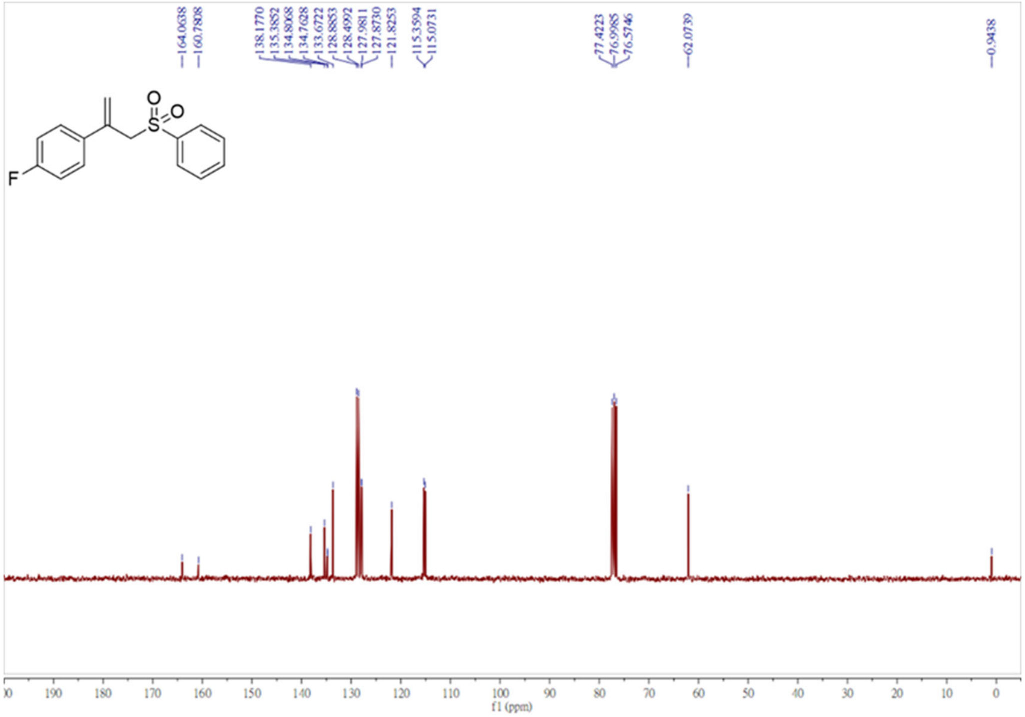

^{1}H NMR (300 MHz, $CDCl_3$) and ^{13}C{^{1}H} NMR (75 MHz, $CDCl_3$) spectra for 1-chloro-4-(3-(phenylsulfonyl)prop-1-en-2-yl)benzene **(5af)**

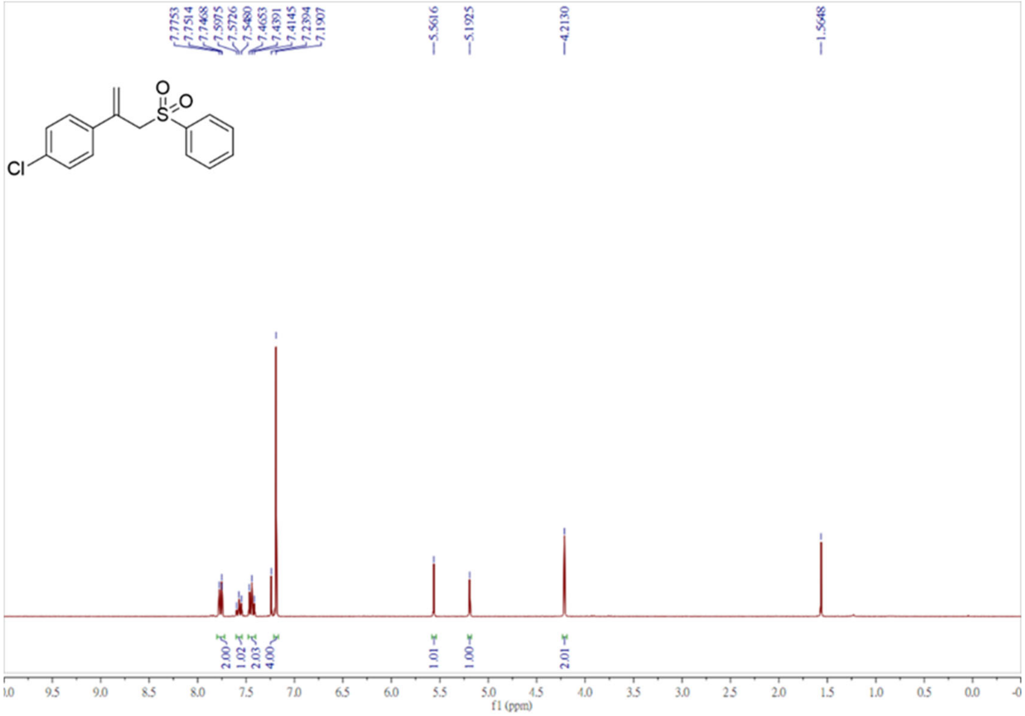

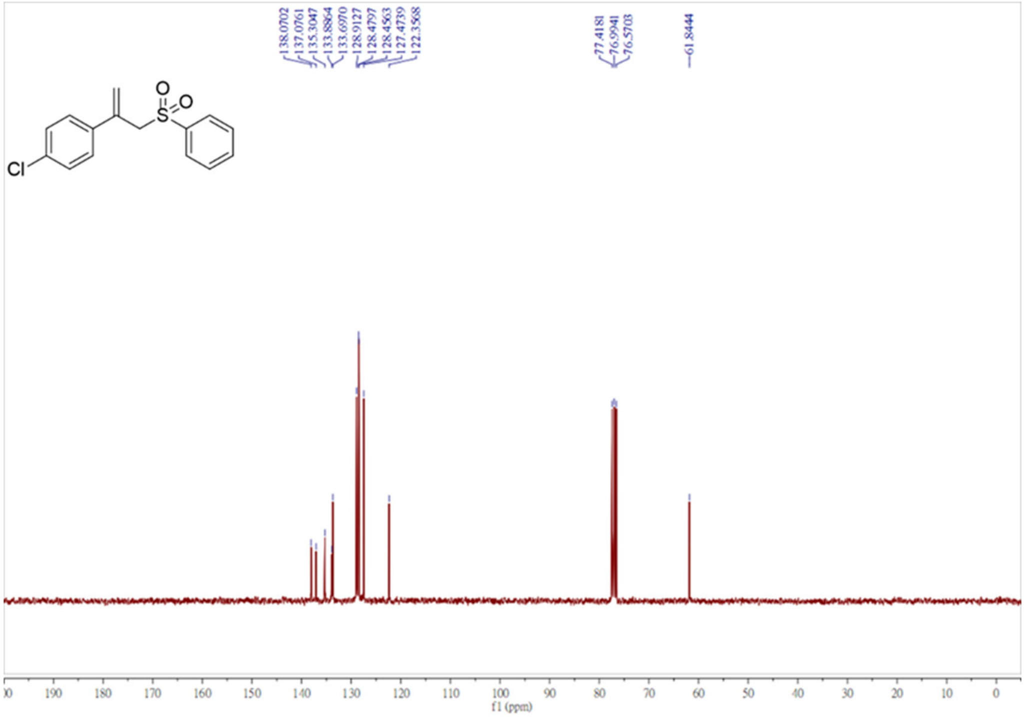

^{1}H NMR (400 MHz, $CDCl_3$) and ^{13}C{^{1}H} NMR (100 MHz, $CDCl_3$) spectra for 1-(4-(3-(phenylsulfonyl)prop-1-en-2-yl)phenyl)ethan-1-one **(5ag)**

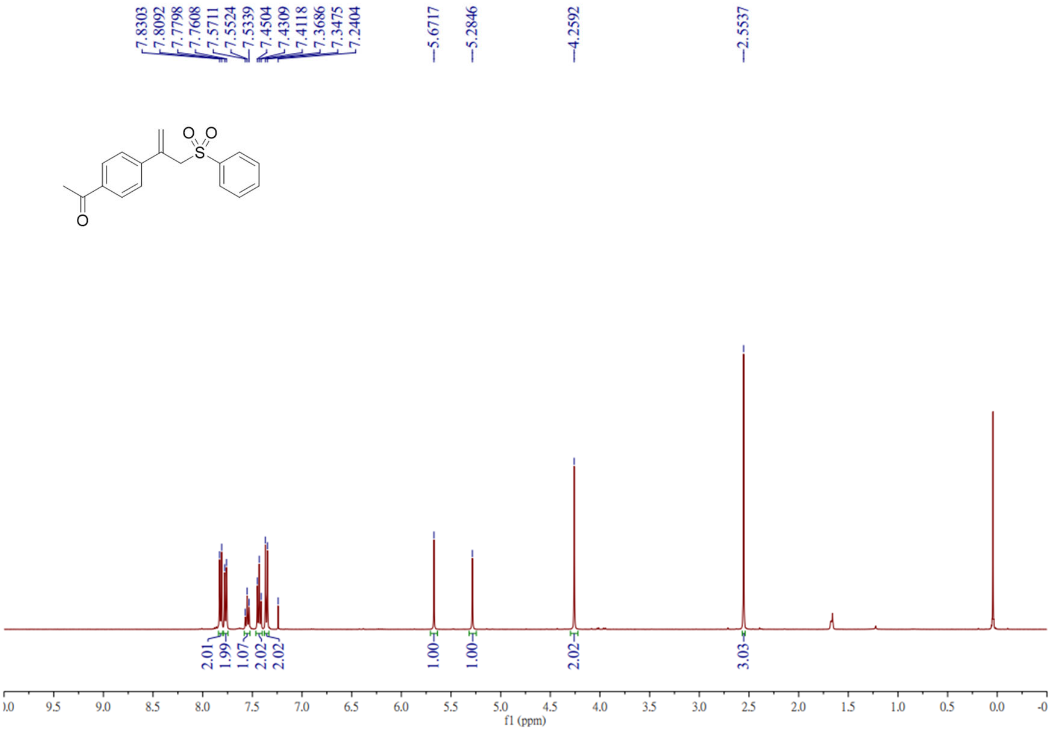

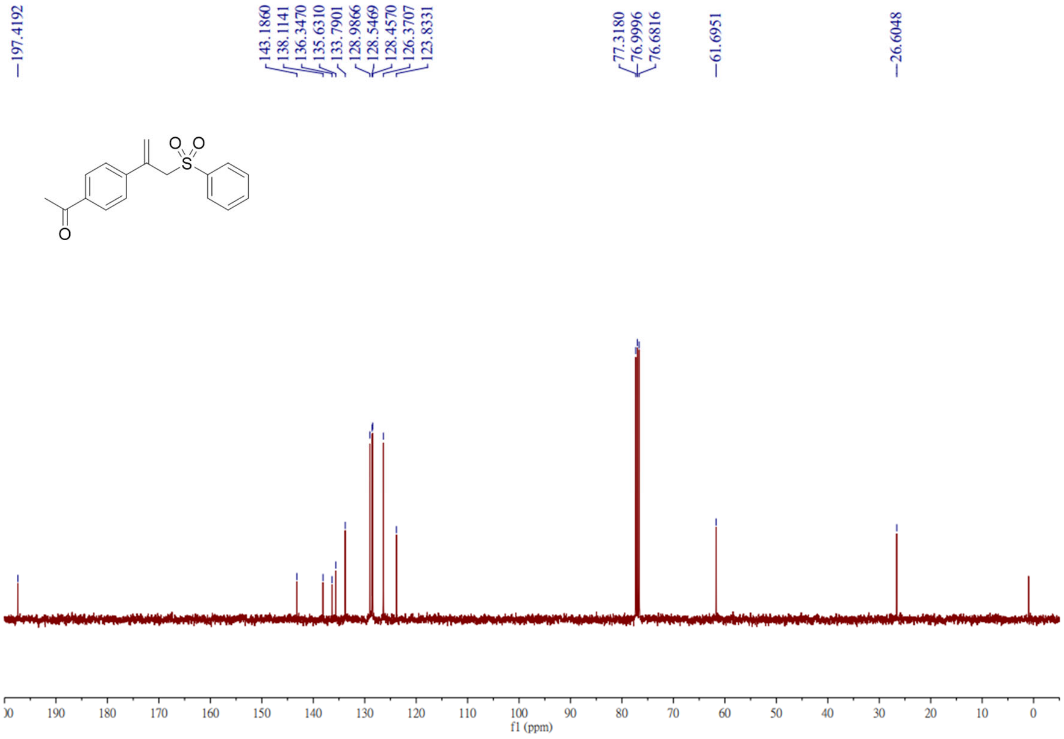

^{1}H NMR (400 MHz, $CDCl_3$) and $^{13}C\{^{1}H\}$ NMR (100 MHz, $CDCl_3$) spectra for 4-(3-(phenylsulfonyl)prop-1-en-2-yl)benzonitrile **(5ah)**

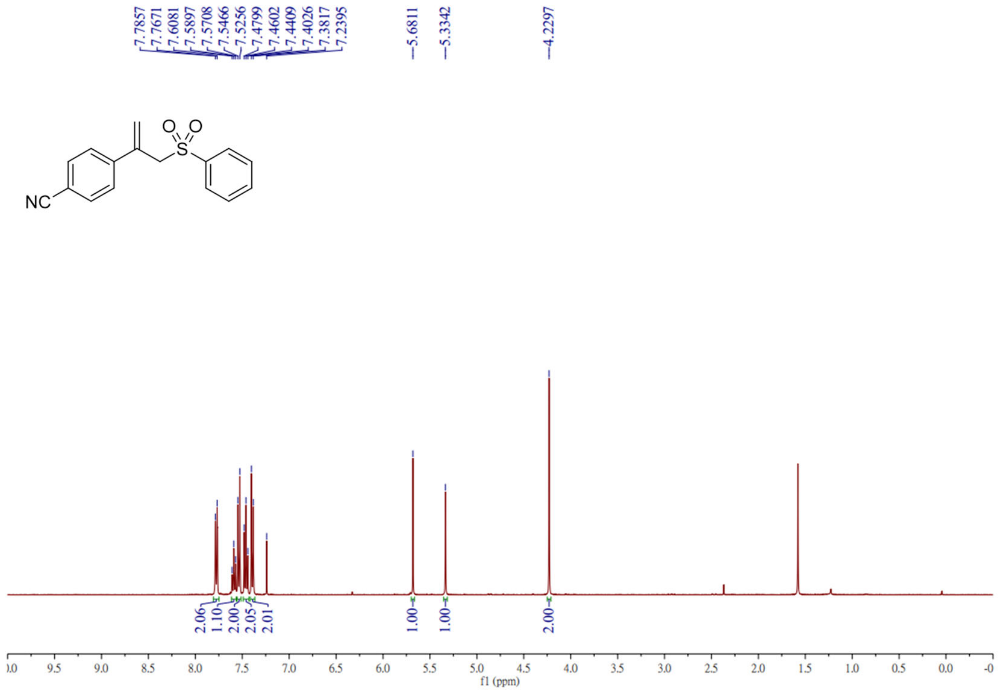

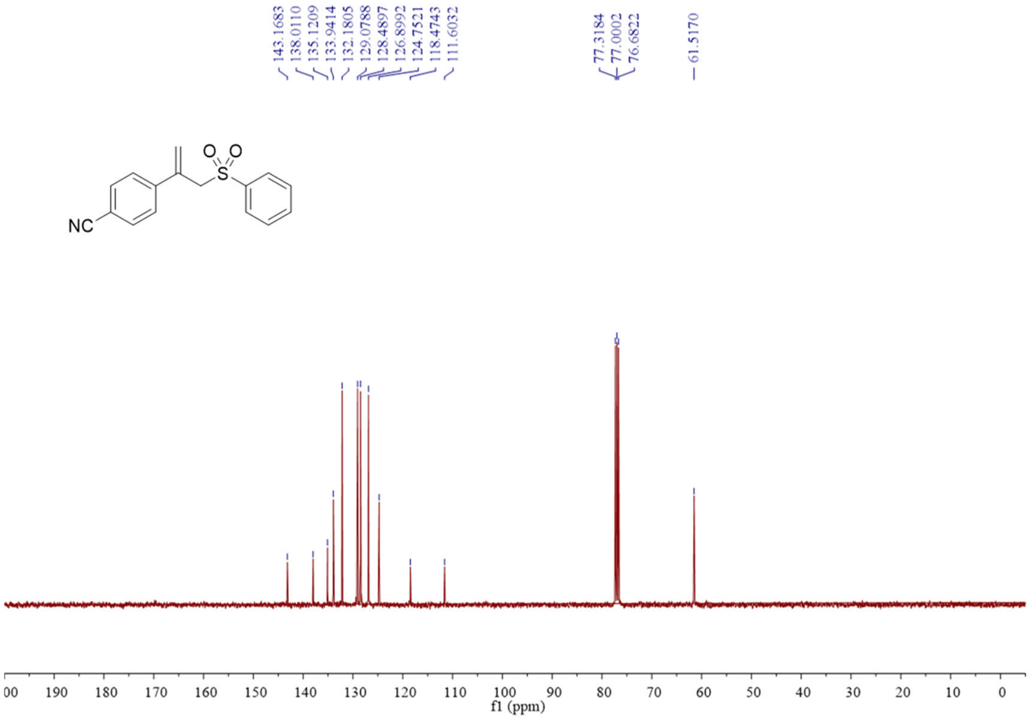

^1H NMR (300 MHz, $CDCl_3$) and $^{13}C\{^1H\}$ NMR (75 MHz, $CDCl_3$) spectra for 1-nitro-4-(3-(phenylsulfonyl)prop-1-en-2-yl)benzene **(5ai)**

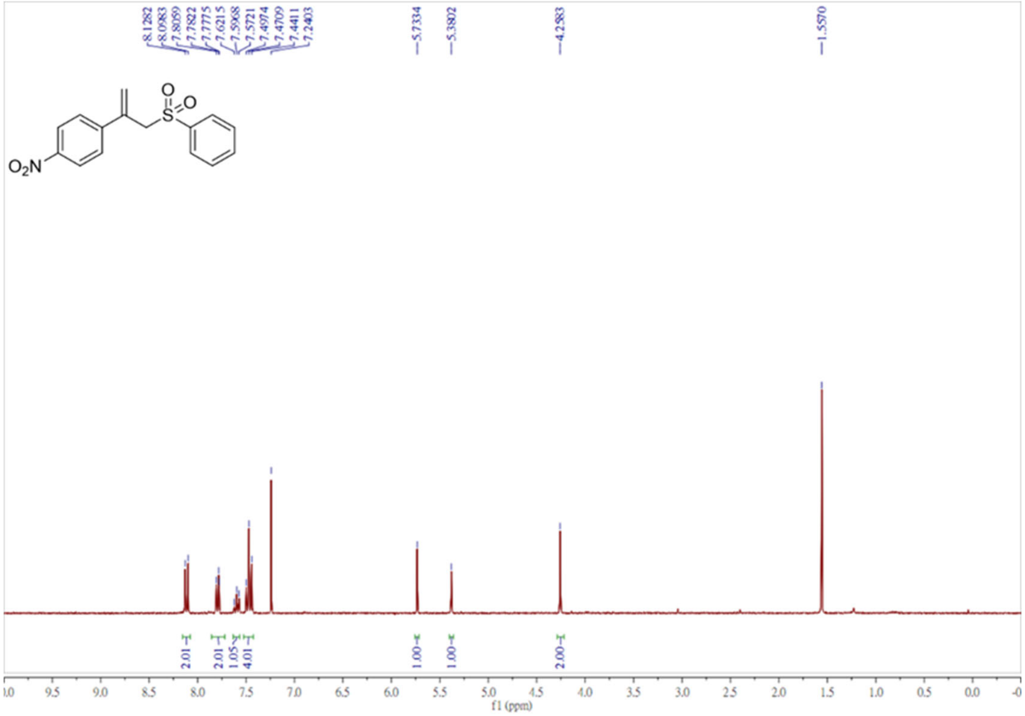

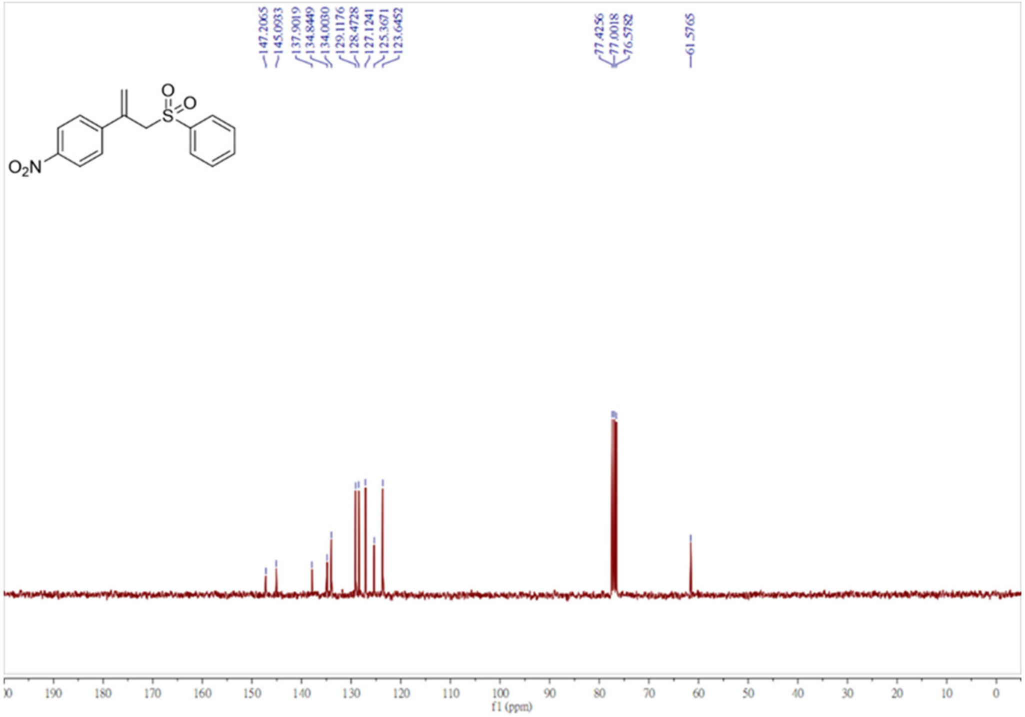

^{1}H NMR (300 MHz, $CDCl_3$) and $^{13}C\{^{1}H\}$ NMR (75 MHz, $CDCl_3$) spectra for *tert*-Butyl 4-(3-(phenylsulfonyl)prop-1-en-2-yl)benzoate **(5aj)**

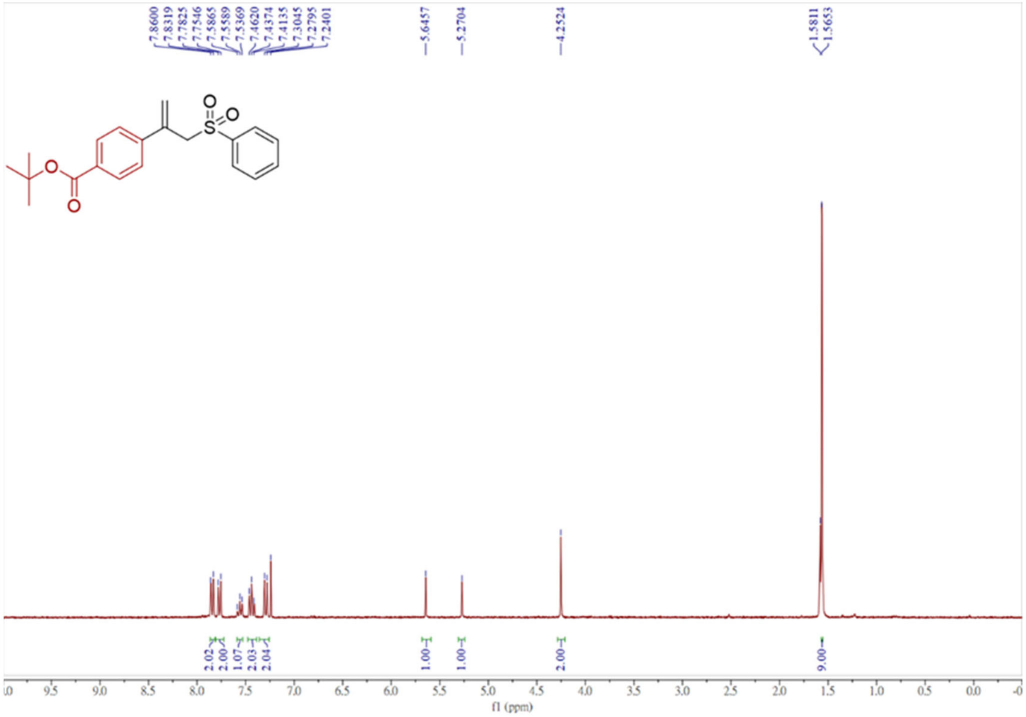

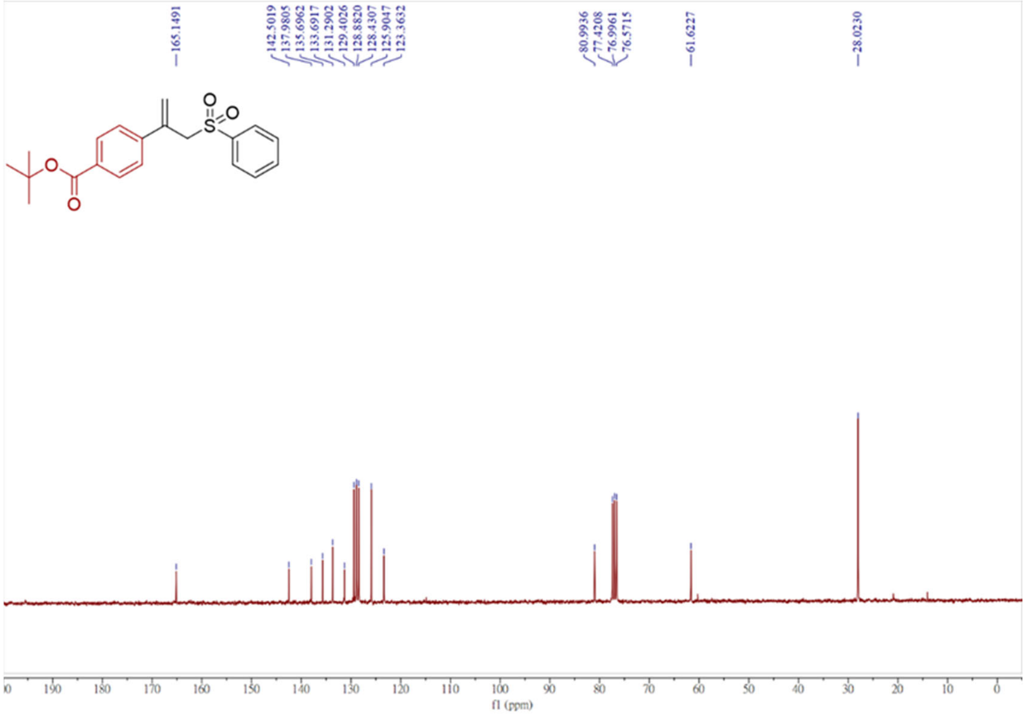

^{1}H NMR (300 MHz, CDCl$_3$) and ^{13}C{^{1}H} NMR (75 MHz, CDCl$_3$) spectra for *N*,*N*-dimethyl-3-(3-(phenylsulfonyl)prop-1-en-2-yl)aniline **(5ak)**

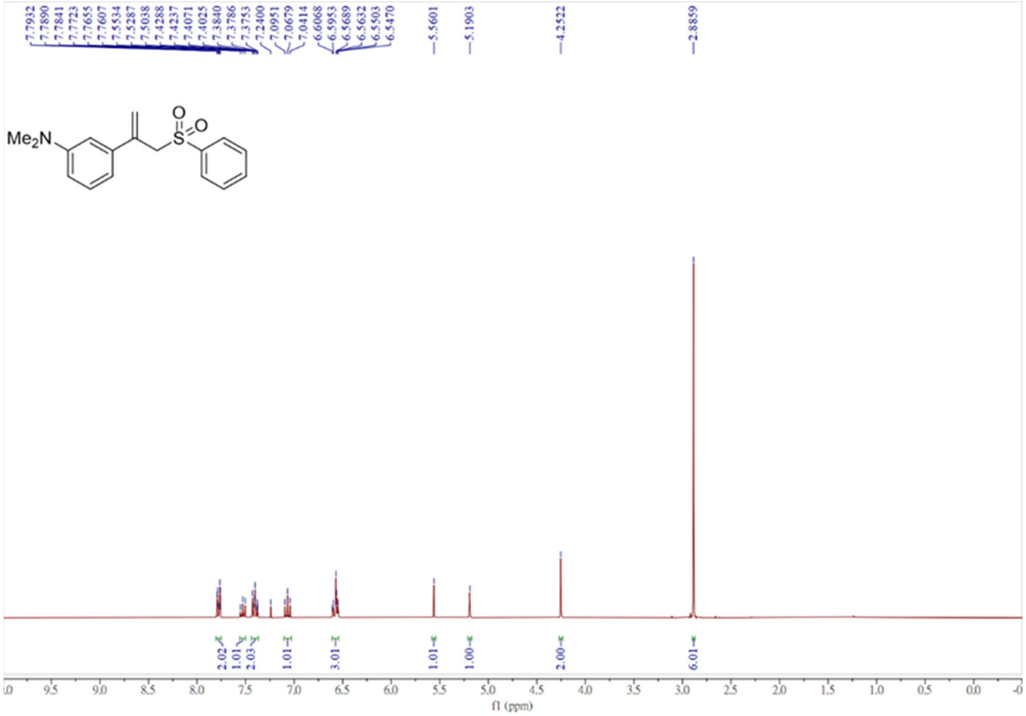

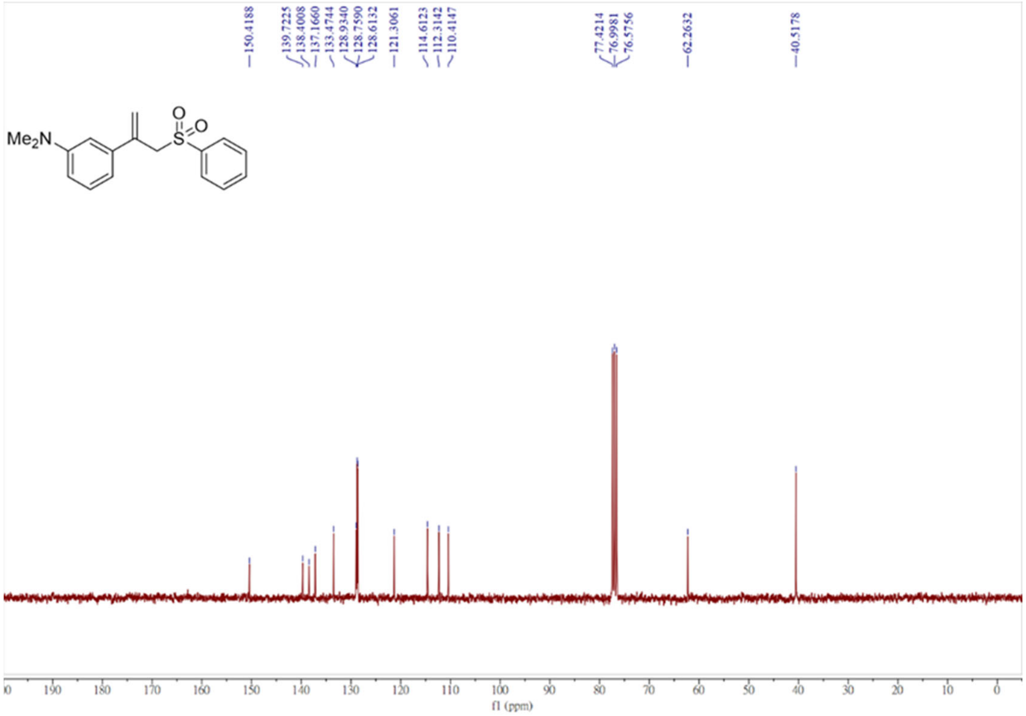

^{1}H NMR (300 MHz, $CDCl_3$) and ^{13}C{^{1}H} NMR (75 MHz, $CDCl_3$) spectra for 1-methoxy-3-(3-(phenylsulfonyl)prop-1-en-2-yl)benzene **(5al)**

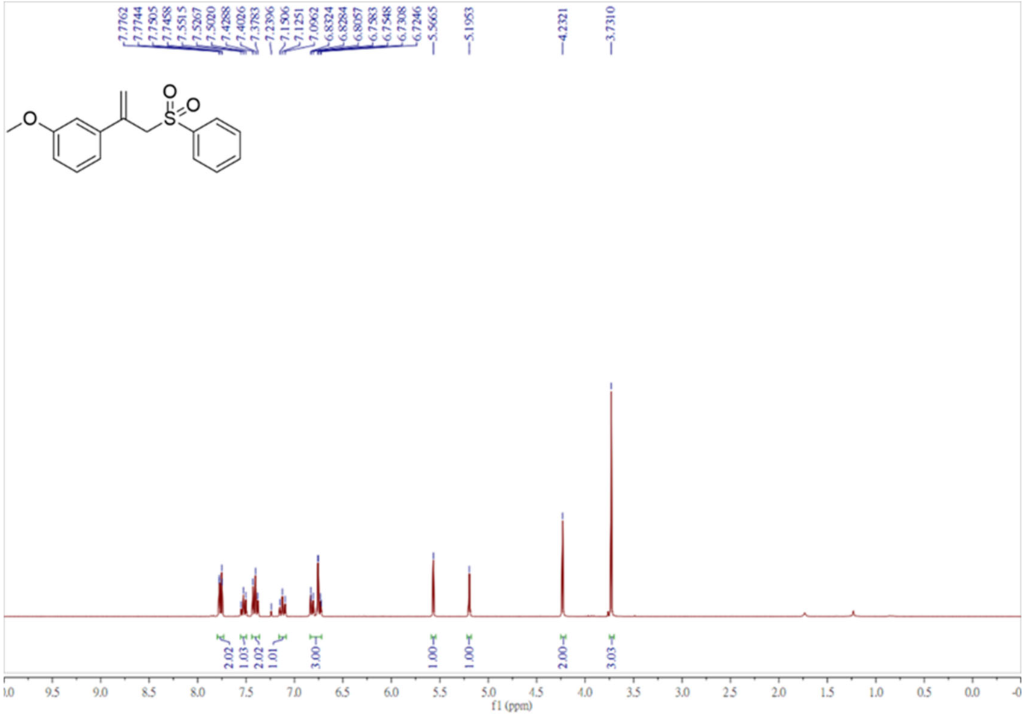

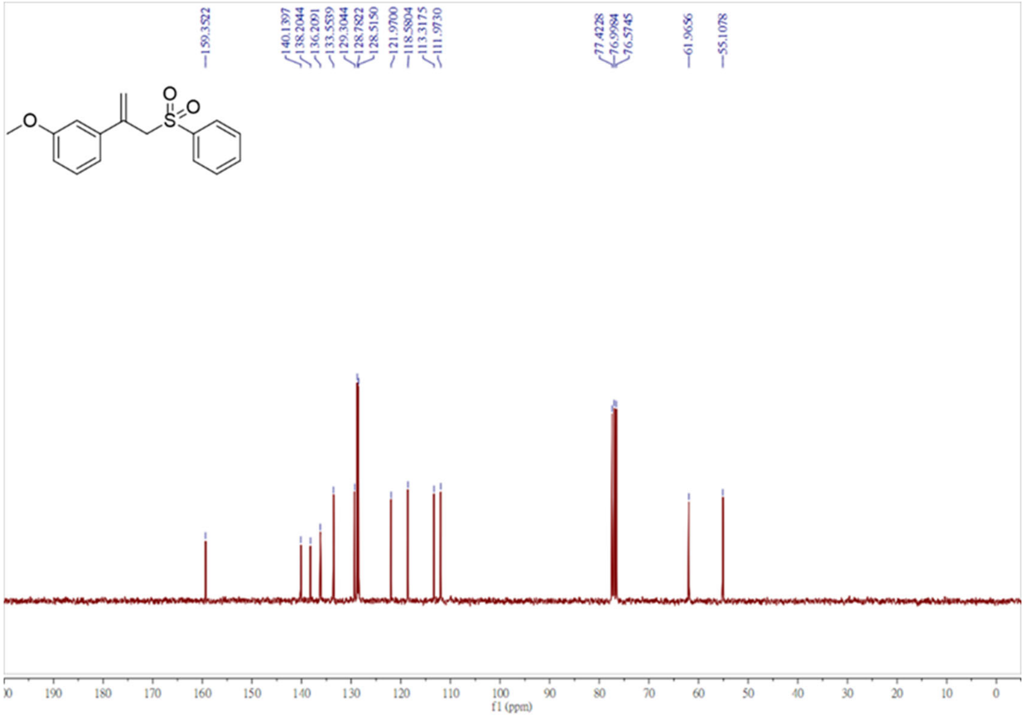

^{1}H NMR (300 MHz, $CDCl_3$) and $^{13}C\{^{1}H\}$ NMR (75 MHz, $CDCl_3$) spectra for 1,3-dimethyl-5-(3-(phenylsulfonyl)prop-1-en-2-yl)benzene **(5am)**

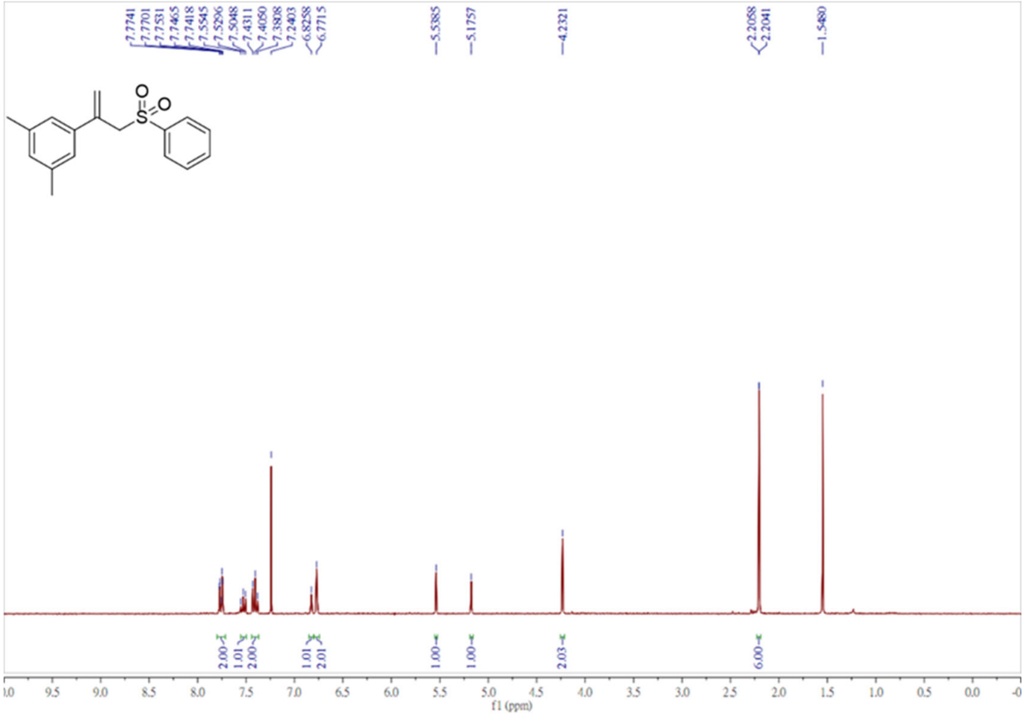

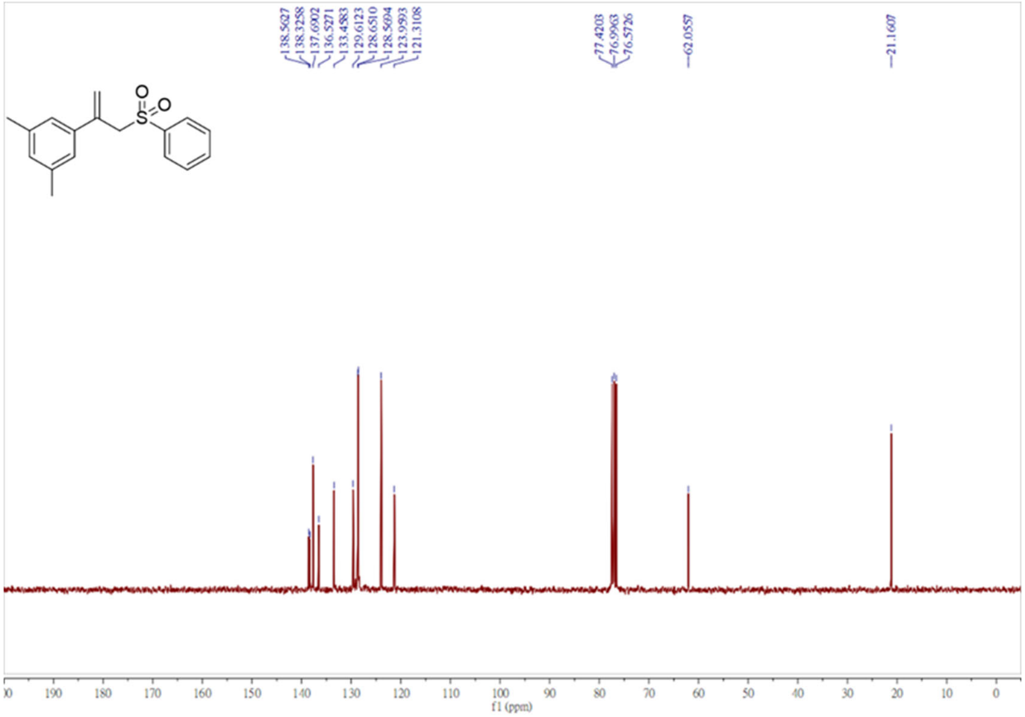

^{1}H NMR (300 MHz, $CDCl_3$) and $^{13}C\{^{1}H\}$ NMR (75 MHz, $CDCl_3$) spectra for 1,2-dimethoxy-4-(3-(phenylsulfonyl)prop-1-en-2-yl)benzene **(5an)**

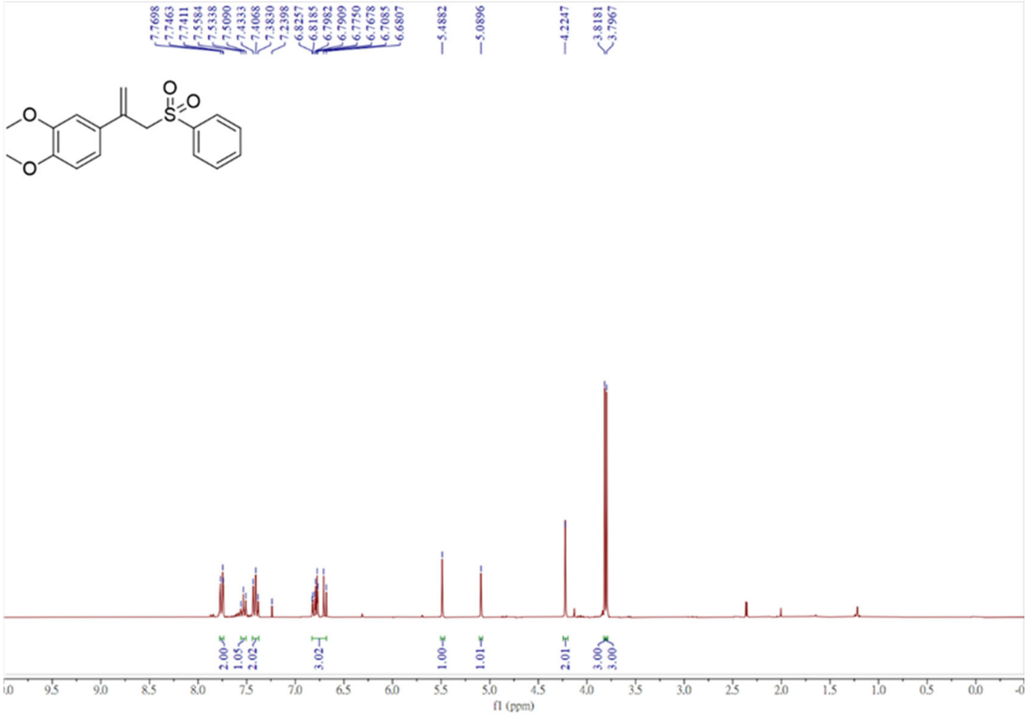

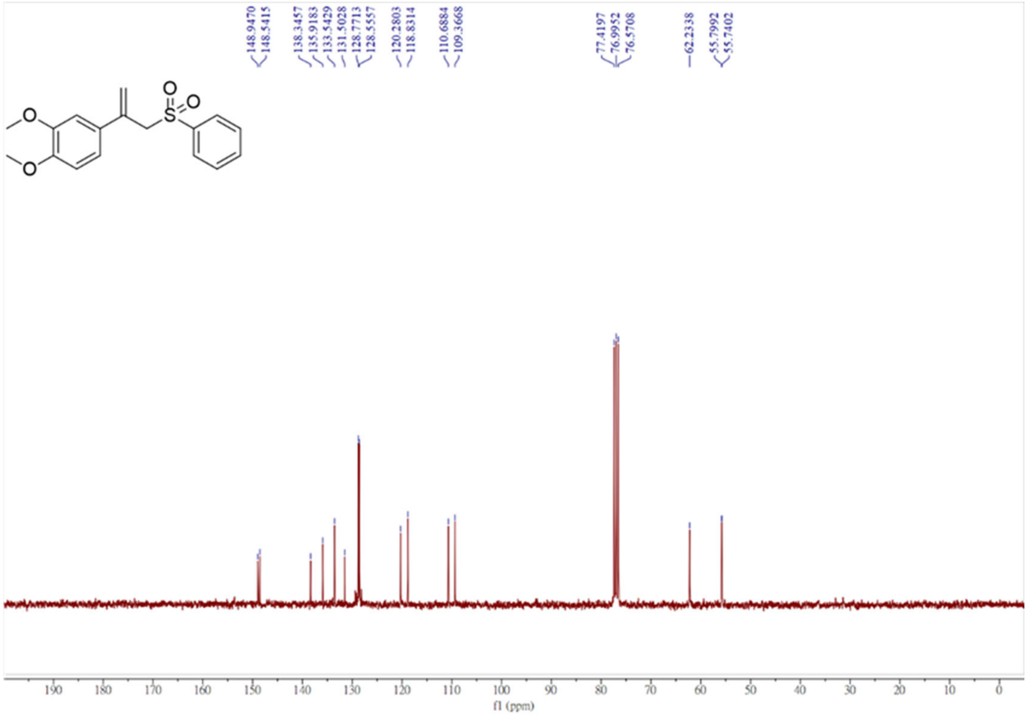

^{1}H NMR (300 MHz, $CDCl_3$) and $^{13}C\{^{1}H\}$ NMR (75 MHz, $CDCl_3$) spectra for 2-(3-(phenylsulfonyl)prop-1-en-2-yl)naphthalene **(5ao)**

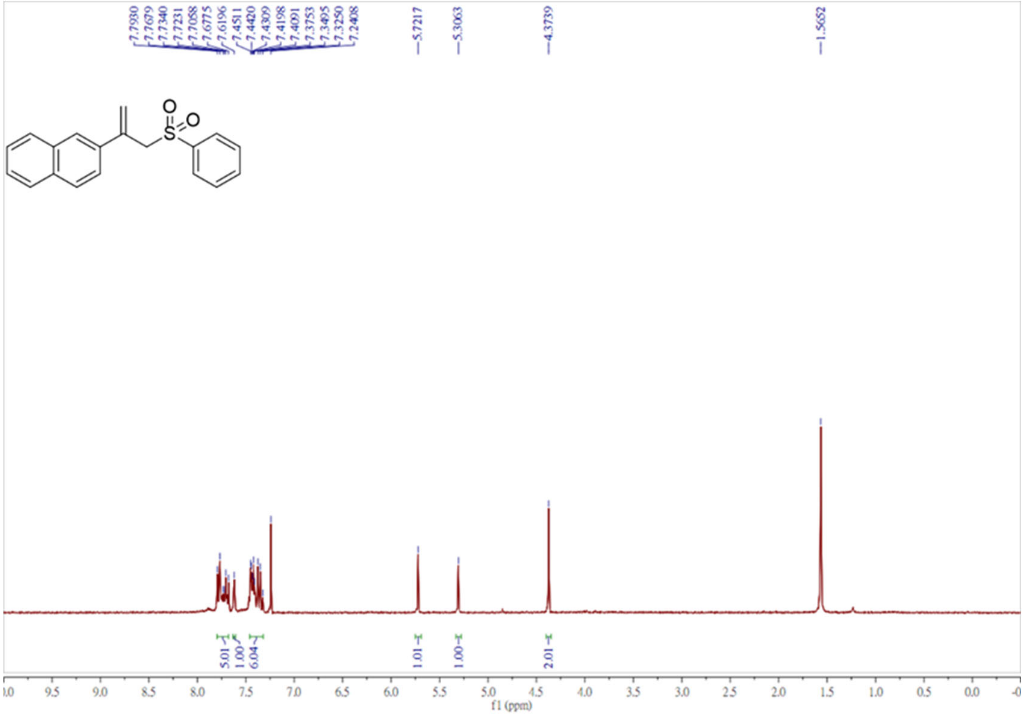

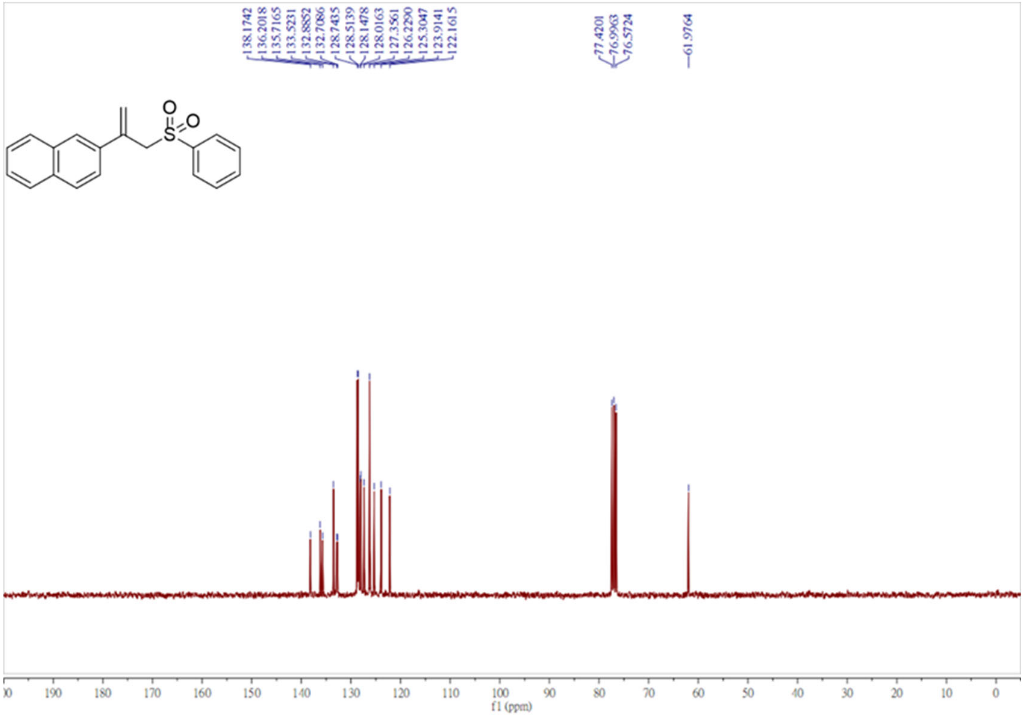

^{1}H NMR (300 MHz, $CDCl_3$) and $^{13}C\{^{1}H\}$ NMR (75 MHz, $CDCl_3$) spectra for 1-(3-(phenylsulfonyl)prop-1-en-2-yl)naphthalene **(5ap)**

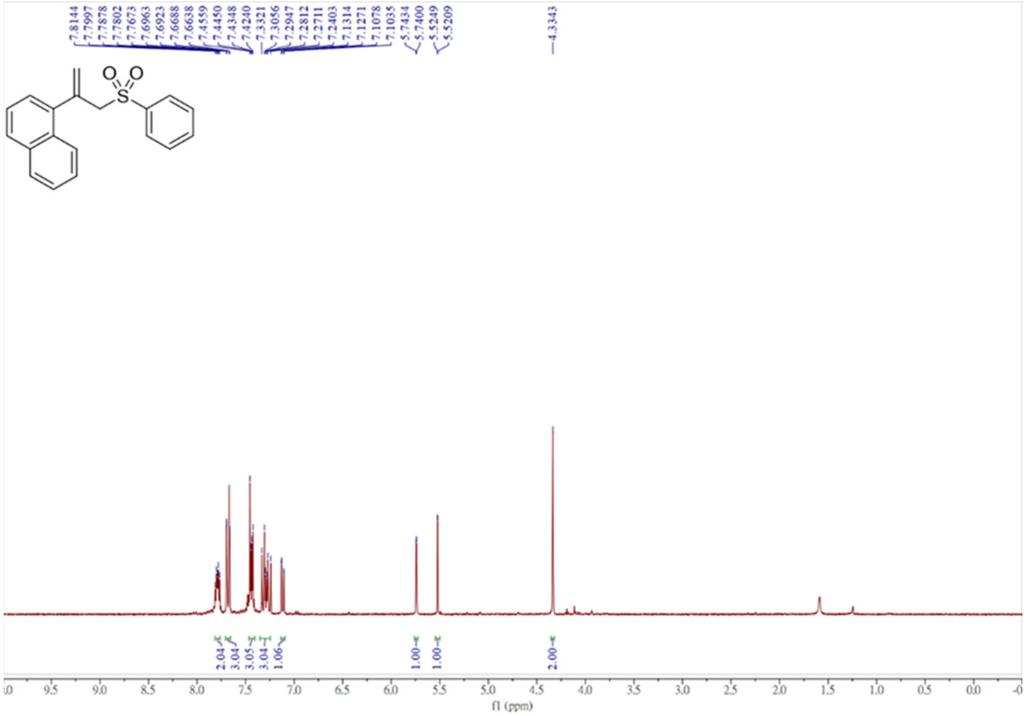

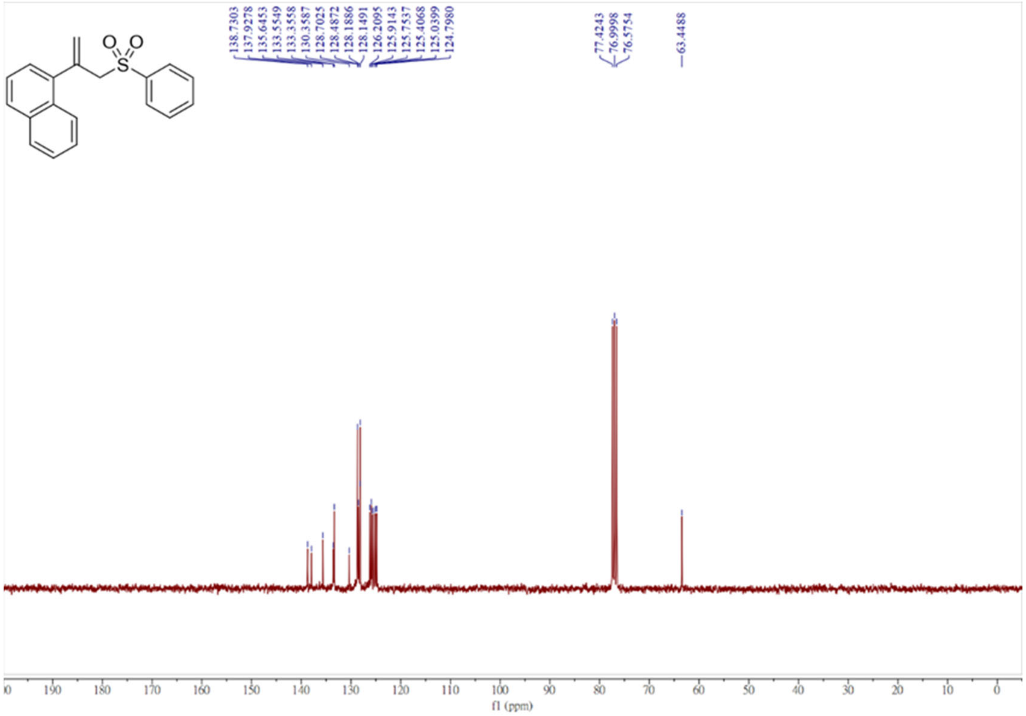

^1H NMR (300 MHz, $CDCl_3$) and $^{13}C\{^1H\}$ NMR (75 MHz, $CDCl_3$) spectra of 1-methyl-2-(3-(phenylsulfonyl)prop-1-en-2-yl)benzene **(5ar)**

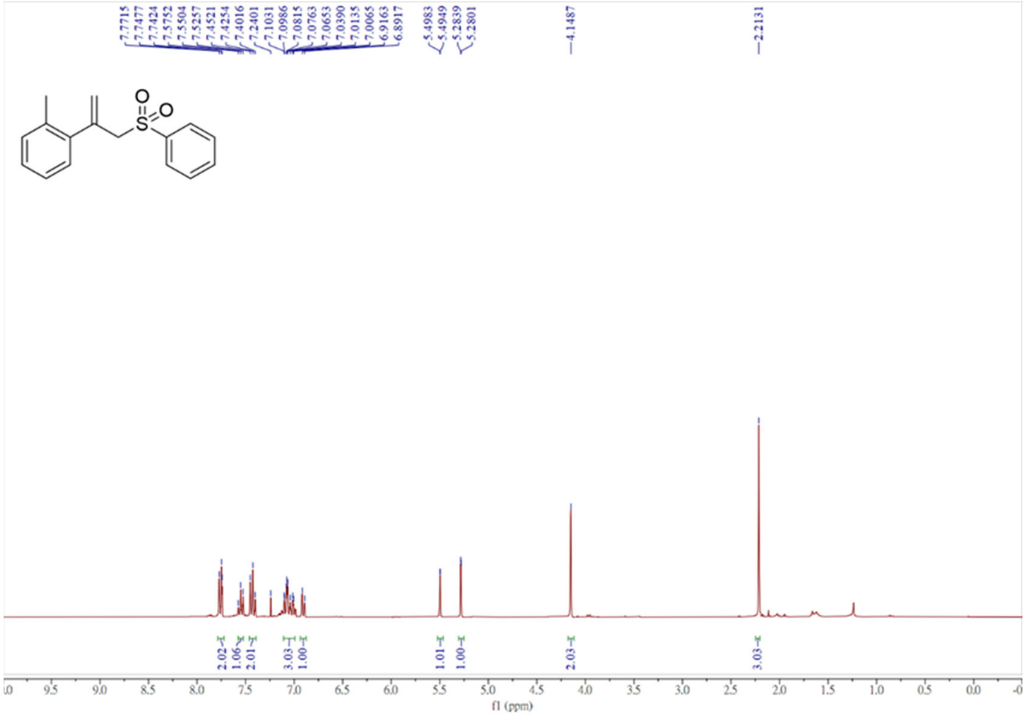

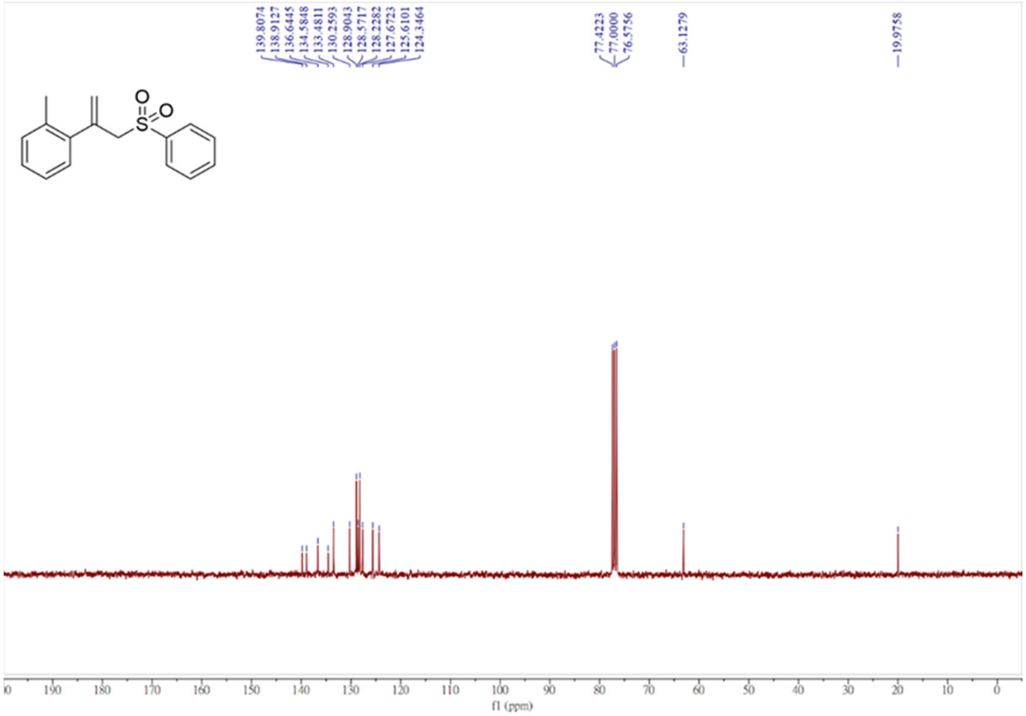

^{1}H NMR (300 MHz, CDCl3), ^{13}C{^{1}H} NMR (75 MHz, CDCl3), and ^{31}P{^{1}H} NMR (162 MHz, CDCl3) spectra for diethyl (2-chloroallyl)phosphonate **(6a)**

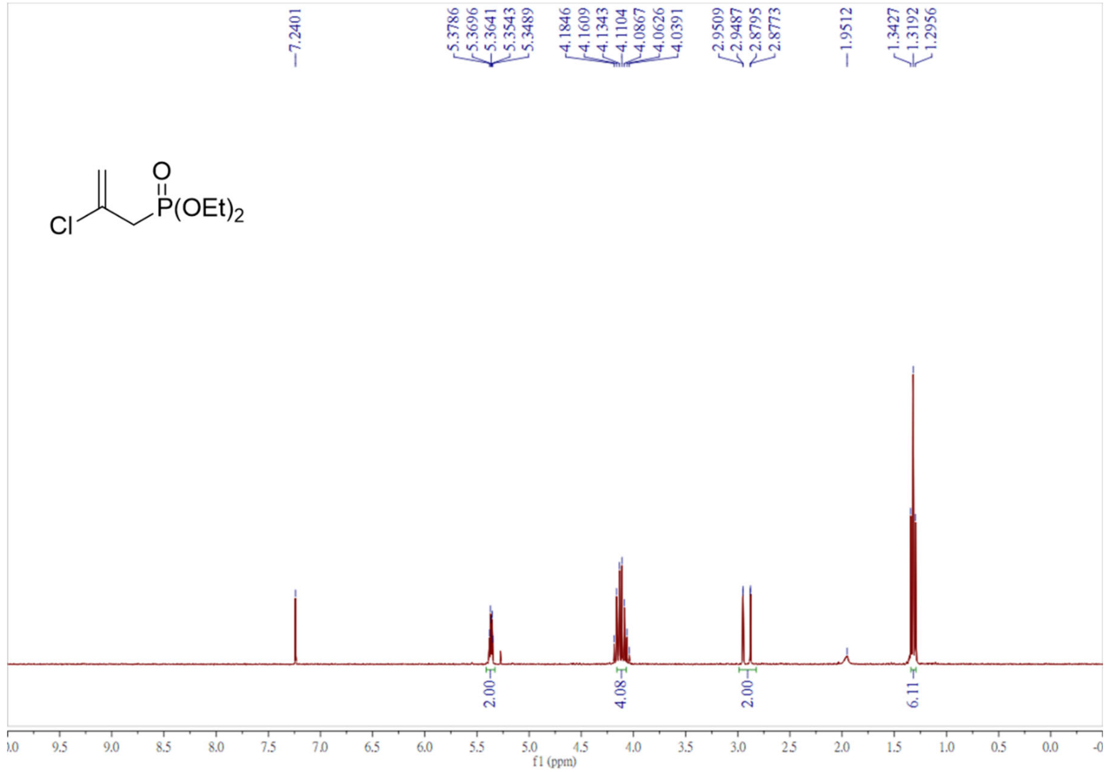

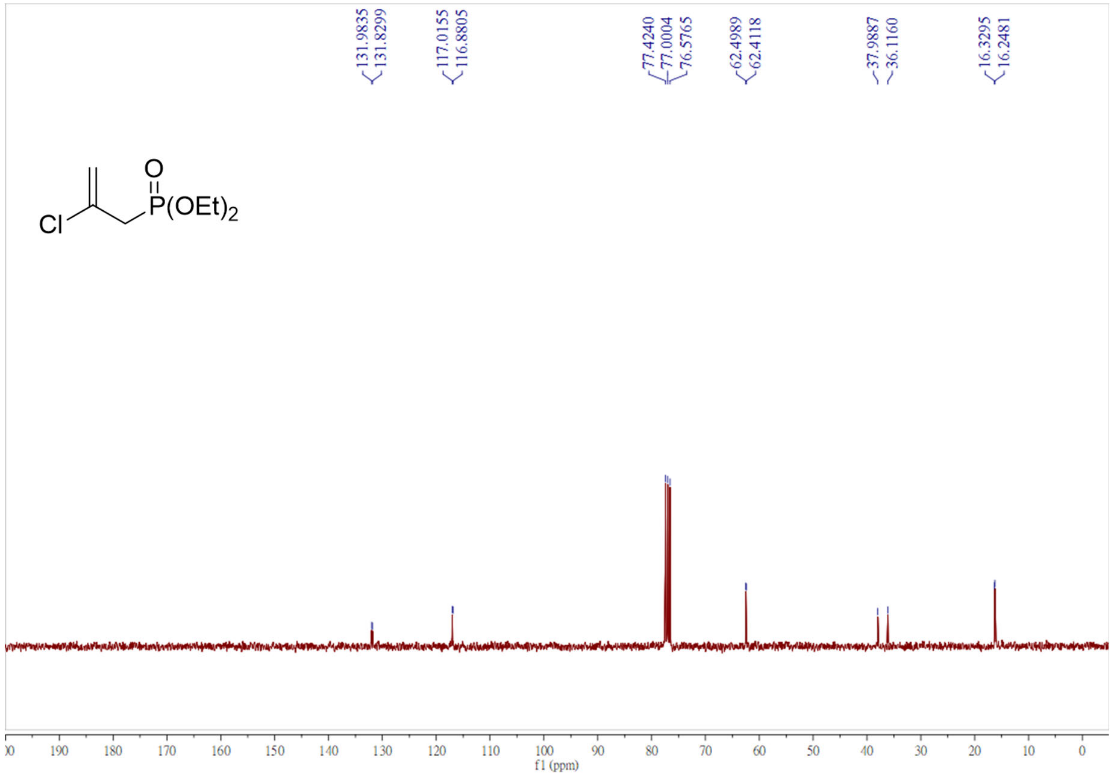

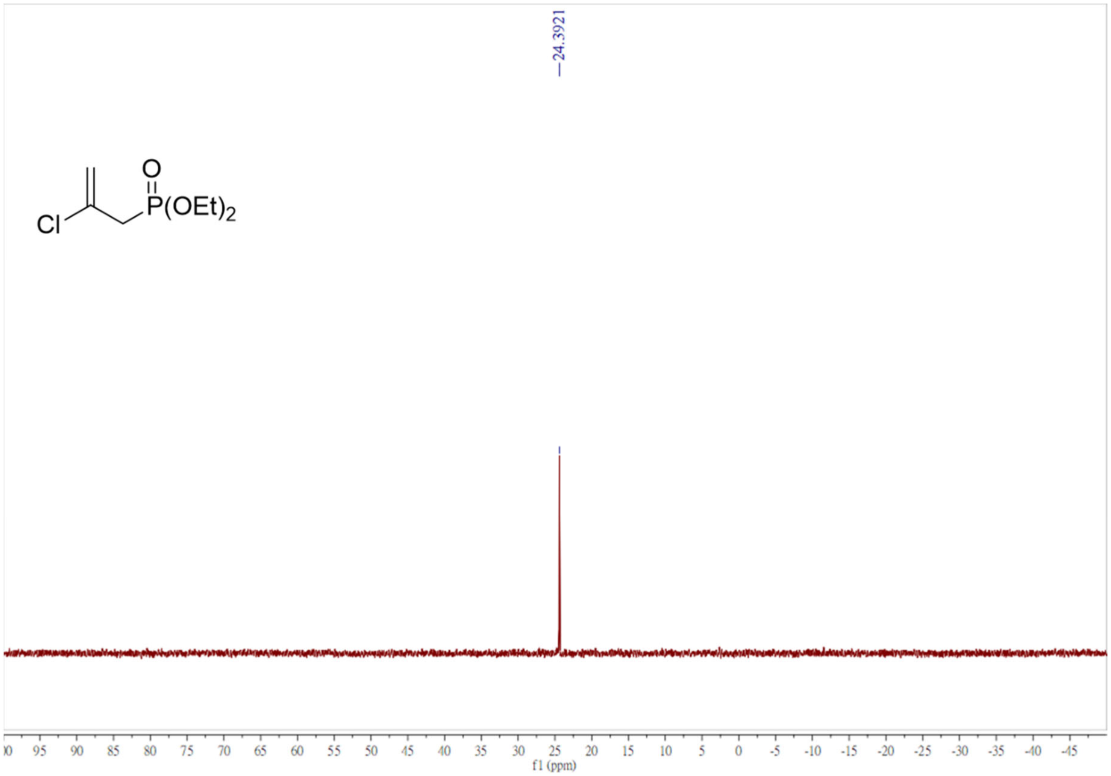

24.3921
Cl
P(OEt)2
O
f1 (ppm)

^{1}H NMR (300 MHz, $CDCl_3$) and $^{13}C\{^{1}H\}$ NMR (75 MHz, $CDCl_3$) spectra for ((2-chloroallyl)sulfonyl)benzene **(7a)**

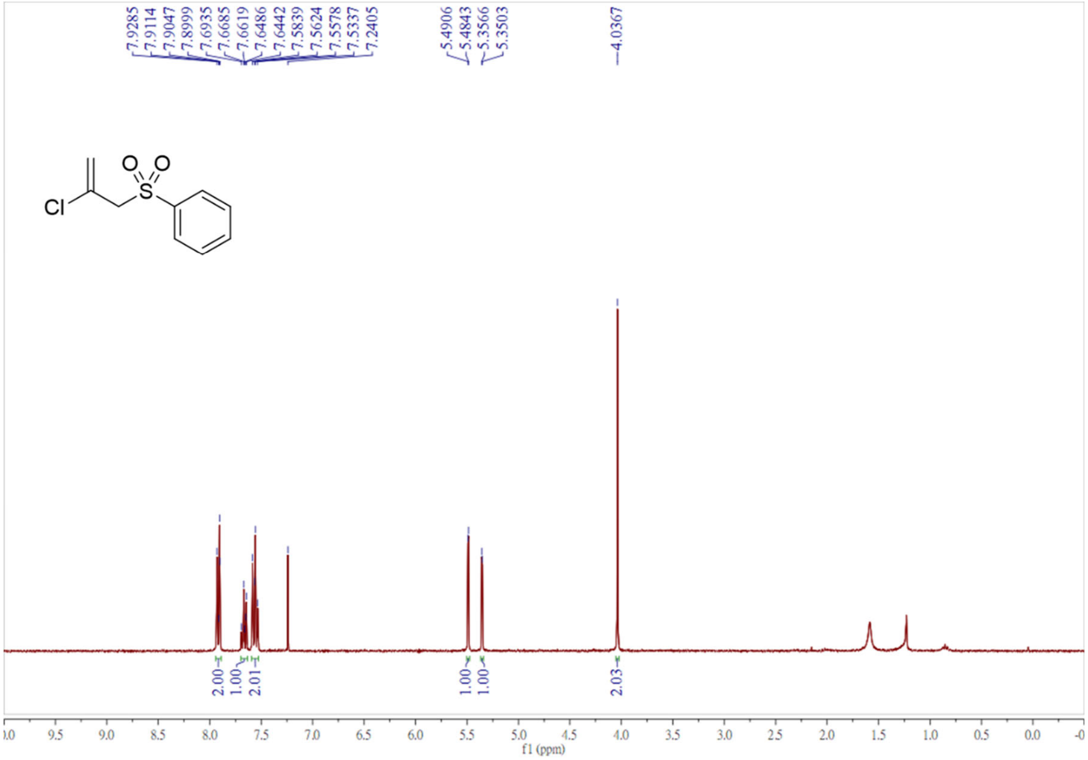

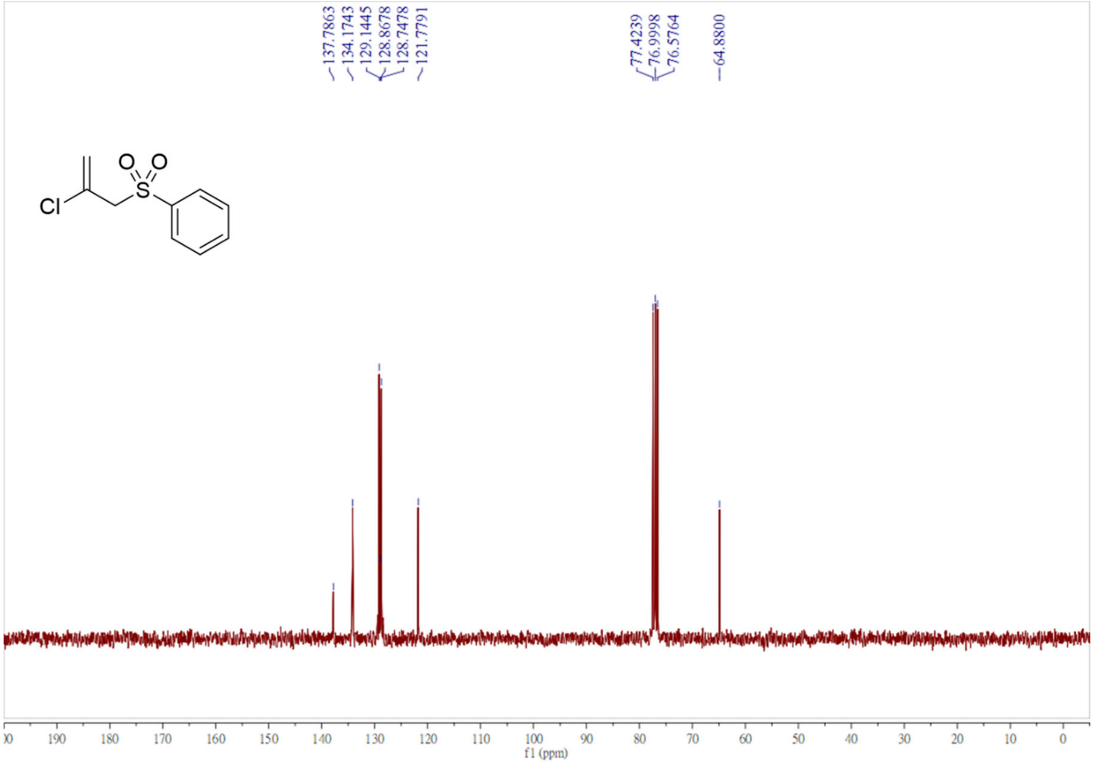

^{1}H NMR (300 MHz, $CDCl_3$), $^{13}C\{^{1}H\}$ NMR (75 MHz, $CDCl_3$), and $^{31}P\{^{1}H\}$ NMR (162 MHz, $CDCl_3$) spectra for diisopropyl (2-phenylallyl)phosphonate **(8)**

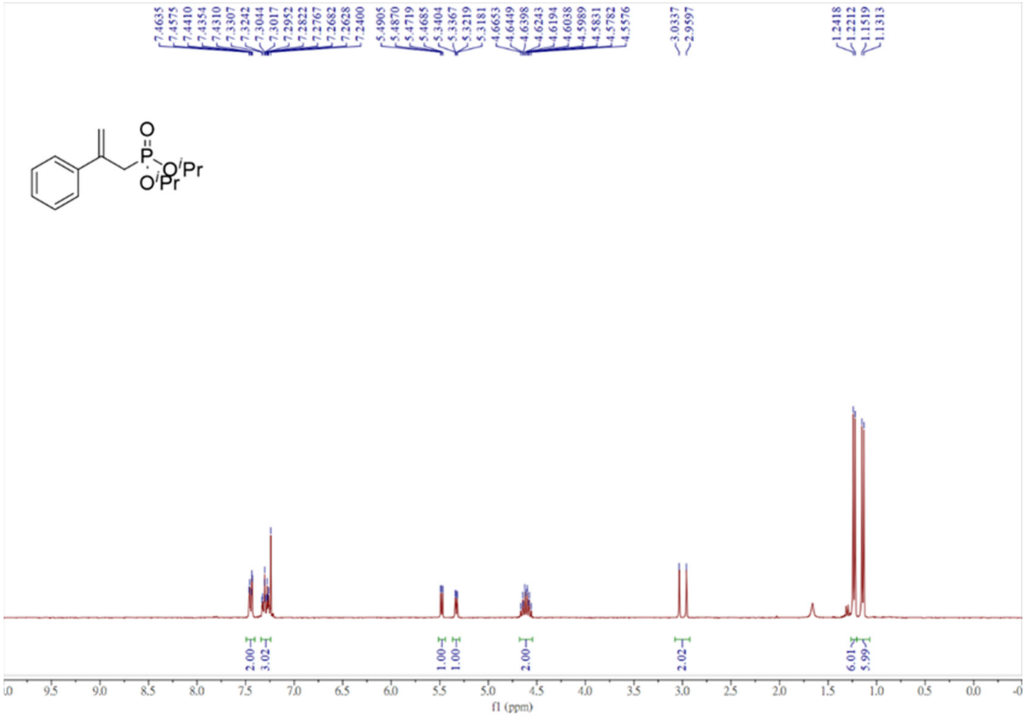

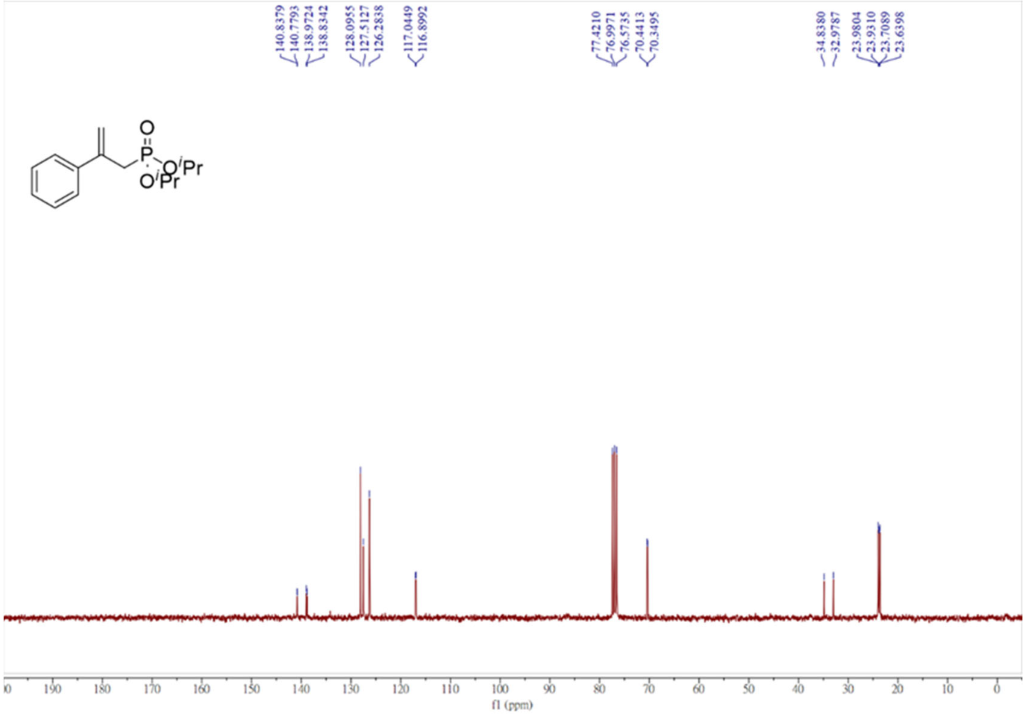

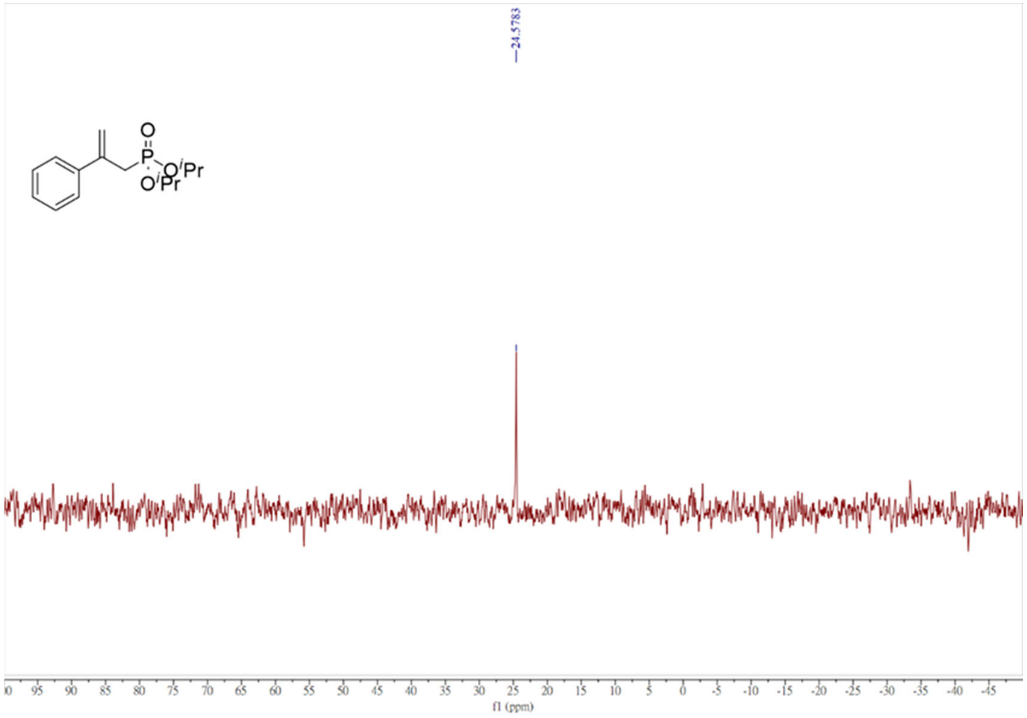

24.5783
O
P
O^{i}Pr
O^{i}Pr
f1 (ppm)

^1H NMR (300 MHz, $CDCl_3$) and $^{13}C\{^1H\}$ NMR (75 MHz, $CDCl_3$) spectra for 1-methyl-4-((2-phenylallyl)sulfonyl)benzene **(9)**

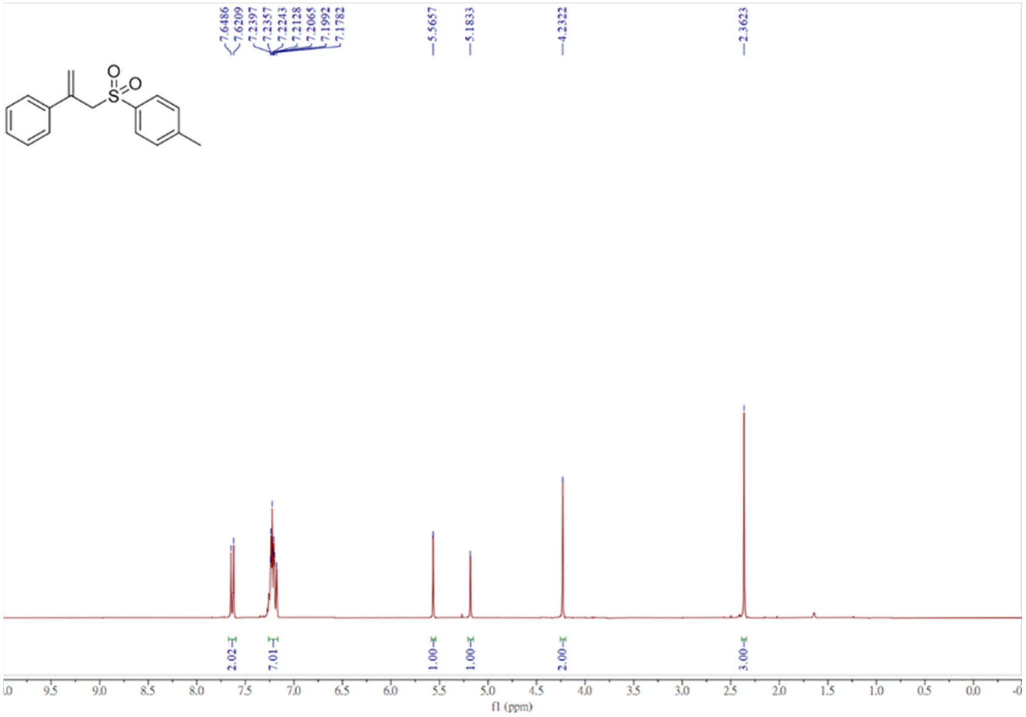

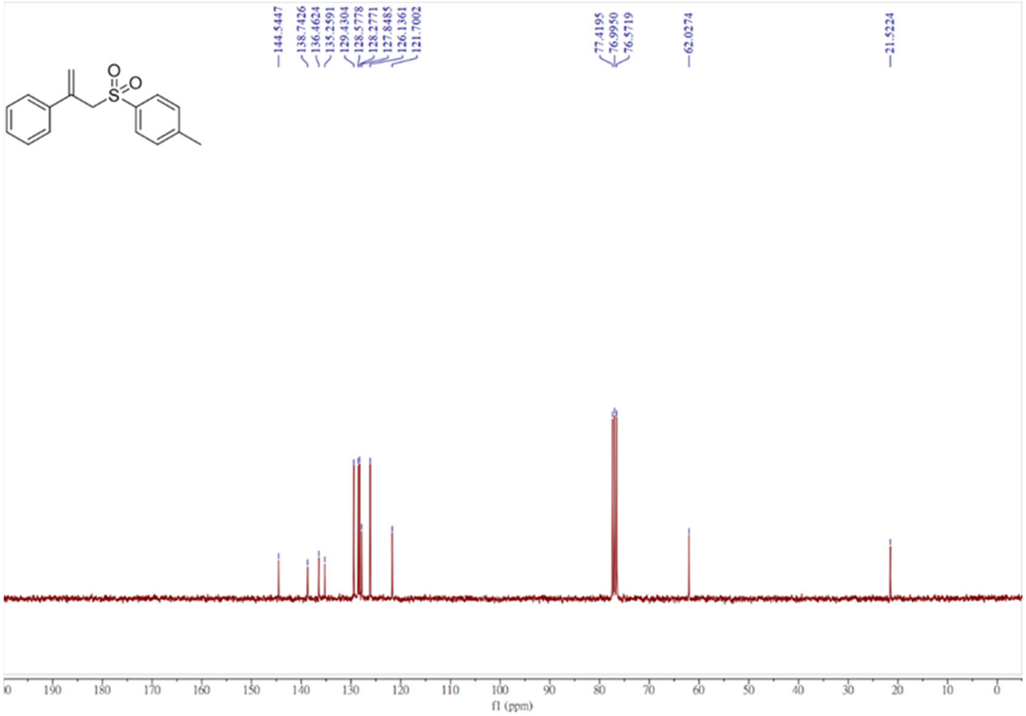

Supplement: Supplementary file 1 — jo3c02455_si_001.pdf [file jo3c02455_si_001.pdf]
